# Identification of a Class of Protein ADP-Ribosylating Sirtuins in Microbial Pathogens

**DOI:** 10.1016/j.molcel.2015.06.013

**Published:** 2015-07-16

**Authors:** Johannes Gregor Matthias Rack, Rosa Morra, Eva Barkauskaite, Rolf Kraehenbuehl, Antonio Ariza, Yue Qu, Mary Ortmayer, Orsolya Leidecker, David R. Cameron, Ivan Matic, Anton Y. Peleg, David Leys, Ana Traven, Ivan Ahel

**Affiliations:** 1 Sir William Dunn School of Pathology, University of Oxford, South Parks Road, Oxford OX1 3RE, UK; 2 Manchester Institute of Biotechnology, University of Manchester, 131 Princess Street, Manchester M1 7DN, UK; 3 Department of Microbiology, Monash University, Malvern East, VIC 3145, Australia; 4 Department of Infectious Diseases, The Alfred Hospital and Monash University, Malvern East, VIC 3145, Australia; 5 Department of Biochemistry and Molecular Biology, Monash University, Malvern East, VIC 3145, Australia; 6 Max Planck Institute for Biology of Ageing, Joseph-Stelzmann-Strasse 9b, Cologne 50931, Germany; 7 Cancer Research UK Manchester Institute, Wilmslow Road, Manchester M20 4BX, UK

## Abstract

Sirtuins are an ancient family of NAD^+^-dependent deacylases connected with the regulation of fundamental cellular processes including metabolic homeostasis and genome integrity. We show the existence of a hitherto unrecognized class of sirtuins, found predominantly in microbial pathogens. In contrast to earlier described classes, these sirtuins exhibit robust protein ADP-ribosylation activity. In our model organisms, *Staphylococcus aureus* and *Streptococcus pyogenes*, the activity is dependent on prior lipoylation of the target protein and can be reversed by a sirtuin-associated macrodomain protein. Together, our data describe a sirtuin-dependent reversible protein ADP-ribosylation system and establish a crosstalk between lipoylation and mono-ADP-ribosylation. We propose that these posttranslational modifications modulate microbial virulence by regulating the response to host-derived reactive oxygen species.

## Highlights

• A class of sirtuins (SirTMs) is identified in microbial pathogens
• SirTMs are linked to macrodomains and act as protein ADP-ribosyltransferases
• Protein ADP-ribosylation by SirTMs is strictly lipoylation dependent and reversible
• SirTMs modulate the response to oxidative stress

## Graphical Abstract

**Figure:**
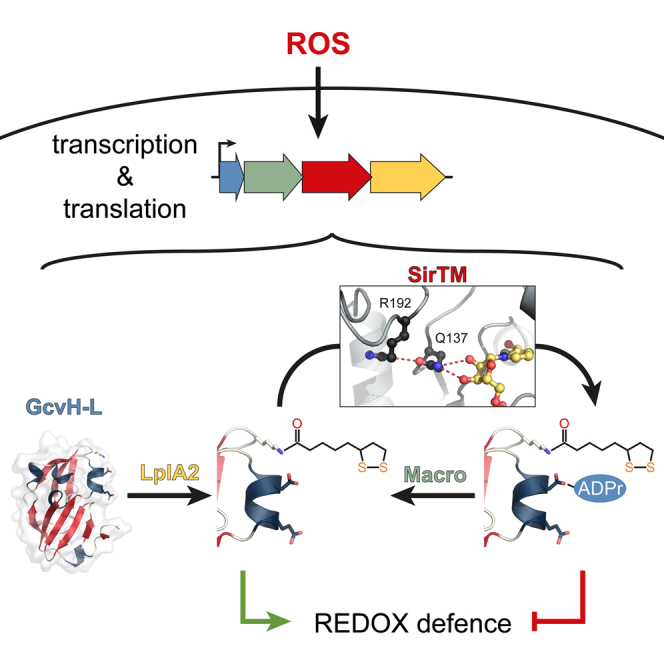

## Teaser

Oxidative stress has been recognized as a critical factor in human disease, aging, and the immune system function. Rack et al. report a structural and biochemical analysis of a sirtuin/macrodomain system modulating the oxidative stress response in pathogenic microorganisms via reversible protein ADP-ribosylation.

## Introduction

**Figure 1:**
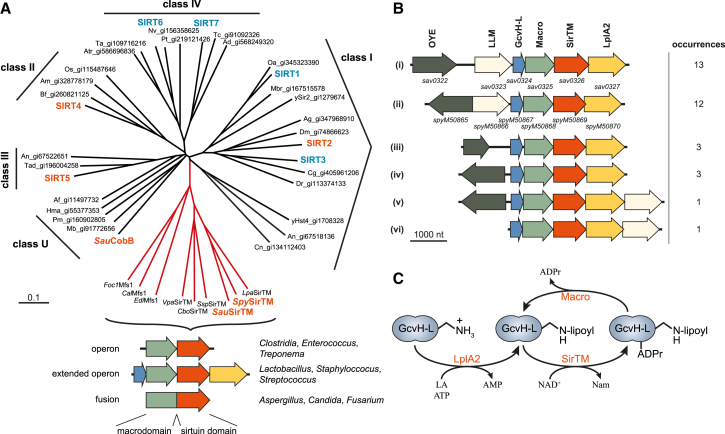
Macrodomain-Associated Sirtuins Form a Distinct Class within the Sirtuin Family (A) Unrooted phylogram illustrating the relationship between the known sirtuin classes (I–IV and U) and sirtuins found associated with macrodomains. The latter form a distinct class among the sirtuins (highlighted in red). Human sirtuins (SIRT1–7) are highlighted in blue and sirtuins used in this study in orange. Schematic genome arrangements of the macrodomain-linked sirtuins are given underneath the tree. Details regarding class M-specific motifs can be found in Figure S1 and Table S1. (B) Schematic overview and organization of the extended SirTM operons. In addition to the sirtuin/macrodomain, the operon encodes a glycine cleavage system H-like (GcvH-L) protein and lipoate protein ligase A (LplA2). The operon is commonly associated with the ORFs of homologs of the Old Yellow Enzyme (OYE) and bacterial luciferase-like monooxygenase (LLM) family (i, ii, and v). Less frequently, association with only one of the two ORFs can be observed (iii, iv, and vi). The encoded proteins are indicated on top of the schemes and the gene loci of *S. aureus* (*sav0322–sav0327*) and *S. pyogenes* (*spyM50865–spyM50870*) are indicated underneath the corresponding schemes. (C) Scheme of the functional relationship between the extended operon components.

Sirtuins are a diverse enzyme family of NAD^+^-dependent protein deacylases that control a variety of cellular processes including cell cycle progression, maintenance of genome integrity, and metabolic homeostasis (Asher and Schibler, 2011; Choi and Mostoslavsky, 2014). The overall structure of sirtuins is comprised of a highly conserved Rossmann fold and a more diverse zinc coordinating domain (Yuan and Marmorstein, 2012). The reaction mechanism is initialized by activation of NAD^+^, followed by a nucleophilic attack and release of nicotinamide. In the case of deacylation, a reactive imidate intermediate is formed that can undergo base-exchange with nicotinamide, thereby inhibiting reaction progression (reviewed in Sauve, 2010). Phylogenetically, the sirtuin family can be divided into five classes (I–IV and U, see Figure 1A) (Frye, 2000; Greiss and Gartner, 2009), and a correlation between sirtuin class and substrate preference was recently suggested (Dölle et al., 2013; He et al., 2012). For example, human SIRT1, a class I sirtuin, is most efficient at deacetylation, whereas SIRT5, belonging to class III, has highest activity toward succinylation (Du et al., 2011; Feldman et al., 2013). Although sirtuins appear to be primarily deacylases, several studies have suggested that some also possess protein ADP-ribosyltransferase activity (Haigis et al., 2006; Kowieski et al., 2008). Posttranslational ADP-ribosylation influences various cellular processes, such as transcription, chromatin organization, nitrogen fixation, and DNA repair, via modification of different acceptor proteins (Barkauskaite et al., 2013; Feijs et al., 2013; Nordlund and Högbom, 2013).

Macrodomains are evolutionary widespread ADP-ribose-binding domains (Till and Ladurner, 2009) that have the potential to reverse sirtuin reactions either by hydrolysis of ADP-ribosylated protein substrates (Barkauskaite et al., 2013), or by deacylating *O*-acyl-ADP-ribose (Chen et al., 2011; Peterson et al., 2011).

In this study, we report on the identification of a distinct class of sirtuins (SirTMs) found primarily in pathogenic microorganisms and show that these function as protein ADP-ribosyl transferases. Members of this sirtuin class are genetically linked to a specific subclass of macrodomain proteins, which reverse the sirtuin catalyzed ADP-ribosylation. Our structural and biochemical analysis suggest that SirTMs possess class-specific features that may explain the preference for protein ADP-ribosylation. Moreover, we show that in *Staphylococcus aureus* and *Streptococcus pyogenes* the sirtuin-mediated ADP-ribosylation is dependent on another posttranslational modification—lipoylation. We propose that a crosstalk between these two types of protein modifications is important for the response of microbial pathogens to oxidative stress, a potent host defense mechanism.

## Results

### Identification of a Distinct Class of Sirtuins

By analyzing available genomic data, we have identified a phylogenetically distinct class of microbial sirtuins (Figure 1A) (Chen et al., 2011 and this study). At the primary sequence level, members of this clade are characterized by a set of short sequence motifs, which are only distantly related to the intra- and interclass sirtuin motifs described by Frye (for details see Figure S1 and Table S1) (Frye, 2000). The most surprising difference is the replacement of the absolutely conserved HG motif containing the catalytic H residue by QG (Figure S1C). These microbial sirtuins are further characterized by their genetic linkage to a subclass of macrodomain proteins. Hence, we designate this class of macrodomain-linked sirtuins as “class M” and their members as “SirTMs.” In bacteria the genes for SirTMs and the associated macrodomains lie adjacent within the same operon. In fungi the two open reading frames (ORFs) are fused to encode a single macrodomain-SirTM polypeptide that we termed macrodomain-fused SirTM 1 (Mfs1). SirTMs are predominantly present in pathogenic organisms including strains in the bacterial *Clostridiaceae*, *Enterococcaceae*, *Lachnospiraceae*, *Spirochaetaceae*, and *Veillonellaceae* families and fungal *Aspergillus*, *Candida*, *Entamoeba*, *Fusarium*, and *Phytophthora* genera, among others (Figure 1A).

### Composition of the Extended SirTM Operon in *Lactobacillales* and *Staphylococcaceae*

Bacterial SirTMs and their macrodomain partners are present as either independent operons or part of extended operons. One type of extended operon can be found in *Lactobacillales* and *Staphylococcaceae,* where the SirTMs are flanked by the ORFs of a glycine cleavage system H-like (GcvH-L) protein and a lipoate-protein ligase homolog (LplA2) (Figure 1B). LplAs are key enzymes for scavenging of the organosulfur cofactor lipoate (an alternative to the de novo synthesis of lipoate) and therefore are essential for virulence of microbial pathogens (Spalding and Prigge, 2010). Canonical GcvH is a component of the multienzyme glycine cleavage system (GCS) involved in glycine detoxification and one-carbon metabolism (Spalding and Prigge, 2010), where it serves as a reaction intermediate shuttle via a conserved lipoylated lysine. The genome of *Staphylococcus aureus* contains both a canonical GcvH associated with the GCS operon and a non-canonical form present in the extended pathogen-specific operon (Table 1). *Streptococcus pyogenes* lacks the GCS system (Spalding and Prigge, 2010 and this study) and its genome encodes only the SirTM-associated form of GcvH. This suggests that GcvH-L supports a GCS-independent function.

In addition, the SirTM operon is associated with two uncharacterized oxidoreductases, one of which exhibits similarities to a flavin-utilizing bacterial luciferase-like monooxygenases (LLM; Pfam: PF00296) and the other to an Old Yellow Enzyme (OYE; Pfam: PF00724) type NADH:flavin oxidoreductase (Figure 1B).

### The LplA2/GcvH-L Pair: An Operon-Specific Lipoylation System

**Figure 2:**
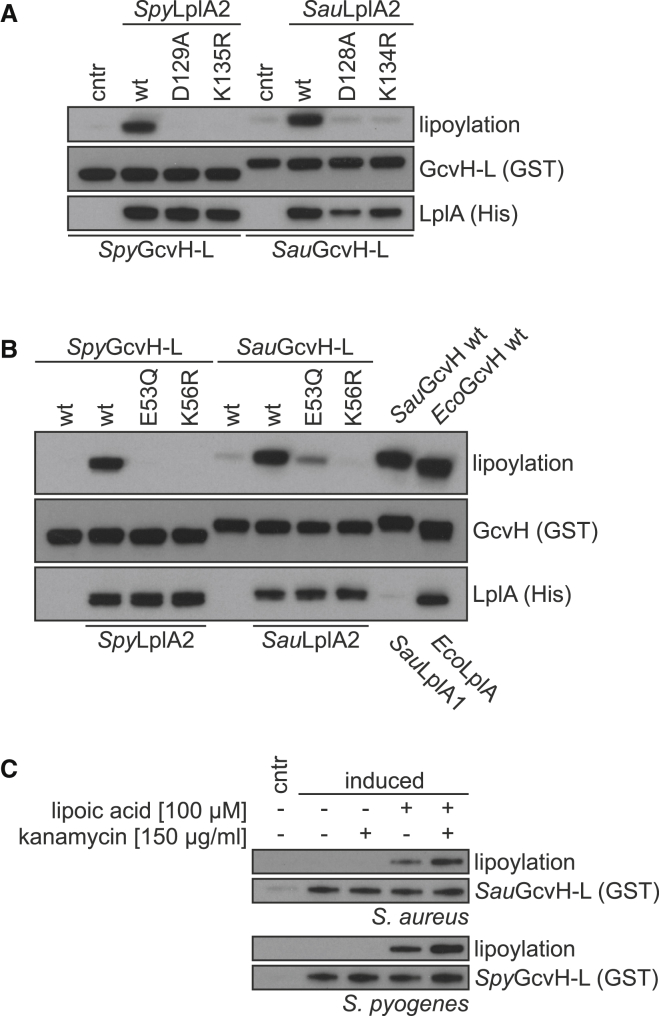
The LplA2/GcvH-L Pair Resembles Its Canonical Homologs (A) Wild-type, but not mutant LplA2, can lipoylate the GcvH-L protein. The mutations were chosen in analogy to the canonical LplA/GcvH pair of *E. coli* where they interfere both with the lipoate adenylation and the subsequent lipoate transfer (Fujiwara et al., 2010). (B) Mutations of lipoylation motif residues within GcvH-L impair the lipoate transfer reaction. Mutation of Lys56 interferes with lipoyl attachment, whereas Glu53 is important for recognition by LplA2 (Fujiwara et al., 1991, 2010). Control reactions were carried out using the canonical LplA/GcvH pairs of *S. aureus* (*Sau*GcvH, *Sau*LplA1) and *E. coli* (*Eco*GcvH, *Eco*LplA). (C) *Sau*GcvH-L and *Spy*GcvH-L were expressed in the presence and absence of lipoic acid supplementation. In addition, protein synthesis of some samples was interrupted by supplementation with kanamycin 1 hr prior to culture harvesting. The effect of the additives on GcvH-L lipoylation was assessed by immunoblot. For further characterization of the LplA2/GcvH-L pair, see Figure S2.

To explore the molecular and biological functions of the SirTMs, we performed a detailed biochemical characterization of the proteins from *S. pyogenes* (*Spy*SirTM) and *S. aureus* (*Sau*SirTM) as representatives of their respective families. Since these sirtuins are part of an operon encoding the LplA2/GcvH-L pair, we hypothesized that they catalyze delipoylation of GcvH-L. Therefore, we first assessed whether the operon-encoded LplA2 can lipoylate GcvH-L, as seen for the canonical LplA/GcvH pairs from *S. aureus* and *Escherichia coli* that served as positive controls. Indeed, both operonal LplA2s efficiently transferred lipoic acid (LA) onto GcvH-L as assessed by immunoblot (IB) using a specific anti-lipoylation antibody (Figures 2A, 2B, and S2B). Mutagenesis showed that the lipoyl-attachment site is Lys56 within the conserved Ex(2)Kx(10)G motif (Fujiwara et al., 1991; Spalding and Prigge, 2009). Since *Sau*GcvH-L appears to be weakly lipoylated in the absence of LplA2, we investigated whether the recombinant GcvH-L can be co-expressionally modified by the endogenous *E. coli* ligase. First, we performed lipoylation assays testing the ability of *Sau*LplA1, 2 or *Eco*LplA to modify the corresponding GcvH proteins (Figure S2C). While *Sau*LplA2 shows selectivity for GcvH-L, *Sau*LplA1 appears to be indiscriminative toward both *S. aureus* GcvHs. Both enzymes, however, exhibit negligible activity toward *Eco*GcvH. In contrast, *Eco*LplA readily modifies all three GcvHs. To test whether these results are transferable to the expression conditions, we supplemented cultures upon induction of protein expression with LA (Figure 2C). Indeed, this increased the co-expressional lipoylation of GcvH-L. The effect could further be enhanced via inhibition of protein synthesis by kanamycin (doubly treated GcvH-L is termed “in vivo lipoylated” in subsequent experiments).

Together these results suggest that LplA2 is a specific lipoate ligase for GcvH-L and that the LplA2/GcvH-L pair shares several characteristics with the LplA/GcvH pairs of the GCS.

### SirTMs Lack Protein Deacylase Activity

**Figure 3:**
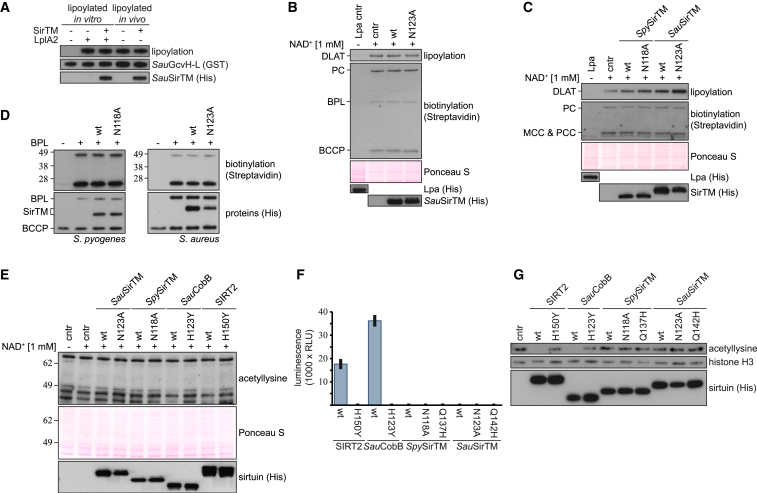
Class M Sirtuins Lack Deacylase Activity (A) Delipoylation assay performed with *Sau*SirTM on in vitro and in vivo lipoylated *Sau*GcvH-L. The in vitro lipoylation was carried out for 30 min prior to addition of SirTM and NAD^+^. Control samples with in vivo lipoylated *Sau*GcvH-L were treated as “in vitro” samples, however, without addition of *Sau*LplA2 and LA. (B) Delipoylation and debiotinylation assay performed on *S. aureus* cell extracts. For lipoylation only, dihydrolipoamide *S*-acetyltransferase (DLAT) could be detected, whereas BCCP, biotin protein ligase (BPL), and pyruvate carboxylase (PC) could be identified as biotinylated. For assays on *E. coli* cell lysates, see Figure S3A. (C) Delipoylation and debiotinylation assays were performed as in (B), but using human 293T cell extract. For lipoylation only DLAT could be detected, whereas PC, 3-methylcrotonyl-CoA carboxylase (MCC) and propionyl-CoA carboxylase (PCC) could be identified as biotinylated. (D) Debiotinylation assay using recombinant, in vitro modified BCCP as substrate. Free biotin was removed from the initial biotinylation reaction by passing it twice over a desalting column. (E) Deacetylation activity of SirTMs was tested on BL21(DE3)Δ*CobB* lysates. Human SIRT2 (isoform 2) and *Sau*CobB were used as positive controls. (F) Deacetylase activities of *Sau*SirTM and *Spy*SirTM against a p53-derived peptide were assessed using the SIRT-Glo assay (Promega). Data are background corrected means ± SD of triplicate measurements. (G) Deacetylase activity of sirtuins (compare to F) was tested against penta-acetylated histone H3 (modified residues: K4, K9, K14, K18, and K23).

Next, we tested whether *Sau*SirTM was able to reverse the lipoylation of *Sau*GcvH-L. We performed the assay with in vitro and in vivo lipoylated *Sau*GcvH-L and showed that *Sau*SirTM failed to delipoylate *Sau*GcvH-L (Figure 3A). On the other hand, incubation with lipoamidase (Lpa) from *Enterococcus faecalis* readily removed the lipoyl moiety (Figure S2D).

Since lipoate is a limiting factor for the growth of some pathogenic bacteria, we hypothesized that the SirTMs could act as scavenging enzymes by delipoylating other endogenous or host-derived proteins. To assess this, we performed delipoylation assays using total protein extracts from *S. aureus*, *E. coli*, and human 293T cells as sources of lipoylated protein substrates (Figures 3B, 3C, and S3A). However, we were unable to detect any delipoylation. Similarly, by using HRP-conjugated streptavidin to detect protein biotinylation in the same protein extracts, we did not observe any deacylation activity toward this evolutionary related modification either (Figures 3B, 3C, and S3A). To eliminate the possibility that extract components interfere with the debiotinylation activity of SirTMs, we performed debiotinylation assays with in vitro modified biotin carboxyl carrier proteins (BCCPs), the only biotinylated proteins shared between *S. aureus* and *S. pyogenes* (Table 1; Figure 3D). Again, SirTMs showed no activity against biotinyl-BCCP. These observations suggest that SirTMs do not participate in lipoyl or biotinyl scavenging/removal. This is further supported by our observation that LplA2 can utilize lipoamide, which mimics protein bound LA, as substrate for GcvH-L modification and therefore release of the free LA by scavenging appears not to be required for LplA2 function (Figure S2E).

We extended our deacylation analyses to two other prominent lysine modifications in bacteria—acetylation and succinylation—using IBs with modification-specific pan-antibodies as detection tools. To exclude interference from the endogenous bacterial sirtuin, CobB, we performed the deacetylation assays on the cell extracts of CobB-deficient *E. coli* (BL21(DE3)Δ*CobB*) (Figure 3E). Our results show that SirTMs are unable to remove acetylation from endogenous proteins, whereas the control enzymes *S. aureus* CobB (*Sau*CobB) and human SIRT2 show activity against some of the acetylated cellular proteins. We also performed two additional in vitro deacetylation assays using acetylated histones and p53-derived peptides, both considered to be generic sirtuin targets. Under the assay conditions, neither SirTM displayed any deacetylation activity toward acetylated p53 (Figure 3F) or penta-acetylated histone H3 (Figure 3G). In contrast, both control enzymes, SIRT2 and *Sau*CobB, showed robust deacetylation activity toward these targets. Desuccinylation activity was tested using a fluorescence succinyl-peptide assay as well as non-enzymatically succinylated histone octamers as substrates. Again, we could not detect any activity for the SirTMs against these targets (Figures S3B and S3C).

Taken together, these results suggest that the pathogenic SirTMs lack deacylation activity.

### Class M Sirtuins Are Lipoylation-Dependent ADP-Ribosyltransferases

**Figure 4:**
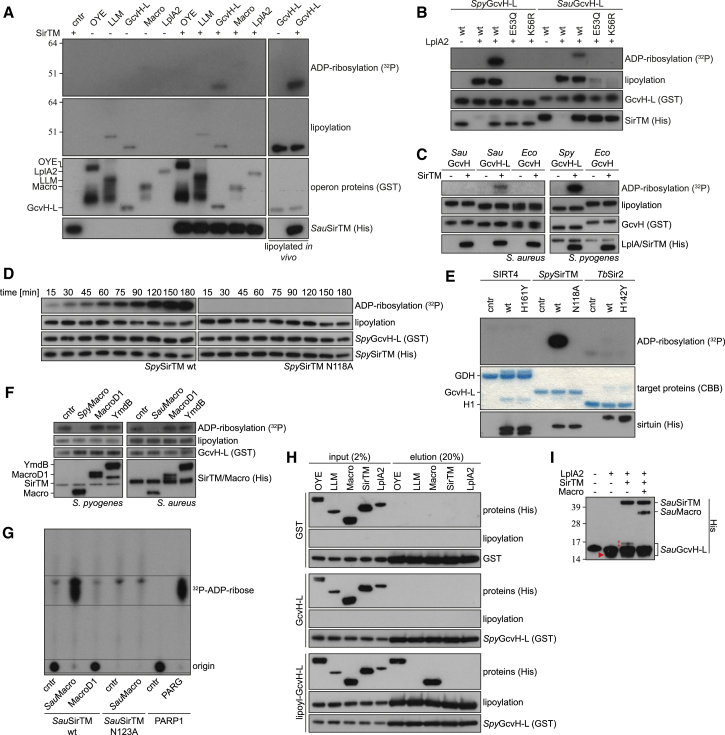
Crosstalk between Lipoylation and ADP-Ribosylation: The ADP-Ribosylation of GcvH-L by SirTM Is Lipoylation-Dependent (A) Mono-ADP-ribosylation assays of operon and associated proteins were performed with GST-tagged target proteins derived from *S. aureus* (indicated on top). Proteins were incubated in the presence and absence of *Sau*SirTM. In addition to the Apo-proteins, in vivo lipoylated GcvH-L was tested (right). To control for self-modification of *Sau*SirTM the enzyme was incubated in the absence of target proteins (cntr). The lipoylated protein observed in the LLM samples corresponds by apparent molecular mass to co-purified *E. coli* DLST. (B) MARylation assays were performed with WT and lipoylation-deficient mutants of GcvH-L. The lipoylation reaction was carried out for 30 min prior to addition of SirTM and NAD^+^. (C) Cross-MARylation assays performed using canonical GcvHs from *S. aureus* and *E. coli* as putative target proteins. (D) Wild-type, but not the catalytic mutant N118A, of *Spy*SirTM modifies lipoylated GcvH-L in a time-dependent manner. (E) Comparison of the ADP-ribosyl transferase activities of human SIRT4ΔMTS and *T. brucei Tm*Sir2 with *Spy*SirTM. The activities were assessed on previously described and herein identified substrates: glutamate dehydrogenase (GDH), histone H1 (H1), and GcvH-L for SIRT4, *Tm*Sir2 and *Spy*SirTM, respectively. Deacetylase activity of *Tm*Sir2 was controlled using a p53-derived peptide as substrate (Figure S3D). (F) ADP-ribosyl hydrolase assays were performed with the operon macrodomains (*Spy*Macro and *Sau*Macro). Control reactions were carried out with the closely related macrodomain proteins MacroD1 (human) and YmdB (*E. coli*) or in the absence of a macrodomain protein (cntr). (G) Identification of the Macro reaction product by TCL. Protein bound ^32^P-ADPr is immobile under the TLC condition (origin), whereas released ^32^P-ADPr co-migrates with the PARP1/PARG (human) control. (H) GST-pulldown assay using GST-fused *Sau*GcvH-L as bait. Prior to pull-down *Sau*GcvH-L was either lipoylated or Lpa treated to define the modification status. The molar ratio of proteins (GcvH-L/GST:operonal) was 1:1. Recombinant GST was used as negative control and binding was monitored by IB. (I) ADP-ribosylation band-shift assay performed with (de)modified *Sau*GcvH-L. *Sau*GcvH-L was incubated consecutively with LplA2, SirTM, and Macro under standard assay conditions (lipoylation 30 min, MARylation 1 hr, and de-MARylation 1 hr). Samples were taken after each reaction step and untreated *Sau*GcvH-L served as control. Under the electrophoretic condition, lipoylation leads to an increased migration (►), while MARylation results in a discrete retention (‡). MARylation was confirmed by its reversibility by Macro treatment. See also Figure S4.

While deacylation is the predominant activity of sirtuins (Du et al., 2009), several studies have reported that some sirtuins also exhibit ADP-ribosyltransferase activity (Haigis et al., 2006; Kowieski et al., 2008). Therefore, we decided to test whether *Sau*SirTM could ADP-ribosylate the purified operon proteins in the presence of ^32^P-labeled NAD^+^. We resolved the reaction products by SDS-PAGE and analyzed them by autoradiography and IB. Among the tested proteins, only *Sau*GcvH-L was modified by *Sau*SirTM (Figure 4A). Furthermore, we observed that prior lipoylation of *Sau*GcvH-L greatly stimulated ADP-ribosyl transfer (Figure 4A, right panel). We further confirmed the presence of ADP-ribosyl group on GcvH-L by the treatment with phosphodiesterase NUDT16 that can hydrolyze protein bound ADP-ribose (Palazzo et al., 2015) (Figure S4A).

### Crosstalk between Lipoylation and ADP-Ribosylation

To confirm the dependence of GcvH-L mono-ADP-ribosylation (MARylation) on lipoylation, we performed lipoylation assays using wild-type (WT) and lipoylation-deficient GcvH-L mutants followed by MARylation reactions using *Spy*SirTM and *Sau*SirTM (Figure 4B). Since only the lipoylated WT GcvH-L was MARylated we concluded that the reaction is dependent on prior lipoylation. To rule out that free LA stimulates the reaction, we performed a MARylation assay following incubation with LA and lipoamide (Figure S4B). MARylation was only observed following covalent linkage of lipoic acid to GcvH-L, thus strengthening the notion that the protein modification rather than free LA is the determining factor. To distinguish between an allosteric stimulation of ADP-ribosylation or a more direct involvement in the reaction, we supplemented MARylation reactions of unmodified GcvH-L with lipoylated peptides (Figure S4C). While lipoylated GcvH-L was readily modified, neither of the lipoylated peptides stimulated ADP-ribosylation activity toward the unmodified GcvH-L, thus suggesting that ADP-ribosylation can only occur as consequence of a prior lipoylation of the same GcvH-L molecule. Analysis of the peptides incubated in the presence of SirTM and NAD^+^ by thin layer chromatography (TLC) showed no incorporation of ADP-ribose (ADPr) into the peptides, hence excluding a competition reaction between peptide and unmodified GcvH-L (Figure S4D). To assess whether the SirTM-mediated ADP-ribosylation is specific for GcvH-Ls, we performed MARylation assays using the lipoylated canonical GcvHs from *S. aureus* and *E. coli* (Figure 4C) as well as biotinylated BCCP as substrates (Figure S4E). Strikingly, SirTMs show absolute selectivity for the lipoylated GcvH-L. Altogether, these data show that lipoylated, full-length GcvH-L is required as substrate in the ADP-ribosylation reaction.

To rule out that the observed ADP-ribosylation is attributed to an indirect non-enzymatic process or represents a weak side reaction, we performed a number of validation assays. First, we confirmed that ^32^P-NAD^+^ is stable under the assay condition, thus ruling out that hydrolyzed NAD products could cause non-enzymatic ADP-ribosylation (Figure S4F). We also performed a time course experiment comparing WT *Spy*SirTM with a catalytically inactive mutant (N118A) (Yuan and Marmorstein, 2012). While the WT enzyme transferred the radiolabel in a time-dependent manner, no transfer was observed for the catalytic mutant (Figure 4D). We could further confirm that a genuine catalytic activity was required for the ADPr transfer by performing the assays in presence of the general sirtuin inhibitors nicotinamide and Tenovin-6 (Figure S4G). While Tenovin-6 inhibited the GcvH-L MARylation, nicotinamide did not significantly affect the reaction. The latter finding is surprising and might indicate that the active site is protected from base-exchange and thus from inhibition by nicotinamide (Sauve, 2010).

Next, we compared the activity of *Spy*SirTM with human SIRT4 and *Trypanosoma brucei Tb*Sir2 that both have described activity as ADP-ribosyl transferases (Fahie et al., 2009; Haigis et al., 2006). While *Spy*SirTM robustly ADP-ribosylates GcvH-L, neither SIRT4 nor *Tb*Sir2 exhibits comparable activity on their described targets (glutamate dehydrogenase and histone H1, respectively) using the same enzyme and substrate concentrations (Figure 4E).

Together, these results provide evidence for a highly specific ADP-ribose transferase activity of SirTMs that depends on the prior lipoylation of the target protein GcvH-L.

### Operon Macrodomains Specifically Reverse SirTM-Mediated ADP-Ribosylation

As some macrodomain-containing proteins are known to hydrolyze protein ADP-ribosylation (Jankevicius et al., 2013; Slade et al., 2011; Sharifi et al., 2013), we assessed the ability of the operon-encoded macrodomains to remove the SirTM-mediated MARylation of the GcvH-L. Strikingly, the operon macrodomains (*Spy*Macro and *Sau*Macro) catalyzed efficiently the modification reversal, as indicated by the loss of radiolabel from GcvH-L, whereas the homologous protein de-MARylating macrodomains from human and *E. coli* (MacroD1 and YmdB, respectively) did not exhibit this activity (Figures 4F and S4H). We further characterized the reaction product of the Macro reaction by TLC. While protein bound ADPr is immobile, the Macro cleavage product co-migrates with ADPr released in the human PARP1/PARG control reaction (Figure 4G) (Slade et al., 2011). The activity of the operonal macrodomains is not absolutely dependent on the lipoylation present on GcvH-L, as revealed by testing the de-MARylation reactions using Lpa-delipoylated GcvH-L as a substrate (Figure S4I). However, GST-pull down assays showed that the macrodomain interacts with GcvH-L in a strictly lipoylation-dependent manner (Figure 4H), thus suggesting lipoylation-dependence in vivo.

Since GcvH-L ADP-ribosylation is part of a coupled reaction, we tested the labeling efficiency within the assays. To this end, all operon reactions were performed consecutively with samples taken after completion of each reaction and subsequently analyzed by electrophoretic gel shift. While lipoylation increased the electrophoretic mobility (Ali et al., 1990), MARylation led to a distinct retention (Figure 4I). We estimate that under our assay conditions, ∼50% of the GcvH-L protein is lipoylated and 5% of the initial substrate becomes ADP-ribosylated. Importantly, MARylation can be fully reversed by addition of *Sau*Macro.

Overall, we conclude that the operon-encoded Macro protein specifically reverses SirTM-dependent ADP-ribosylation and its reaction product is ADP-ribose as seen for other macrodomain proteins (Jankevicius et al., 2013; Sharifi et al., 2013). We propose that the pathogenic SirTM/Macro pair coevolved as an ON/OFF switch for the GcvH-L ADP-ribosylation.

### *Spy*SirTM Structure Reveals an Unexpected Catalytic Residue

**Figure 5:**
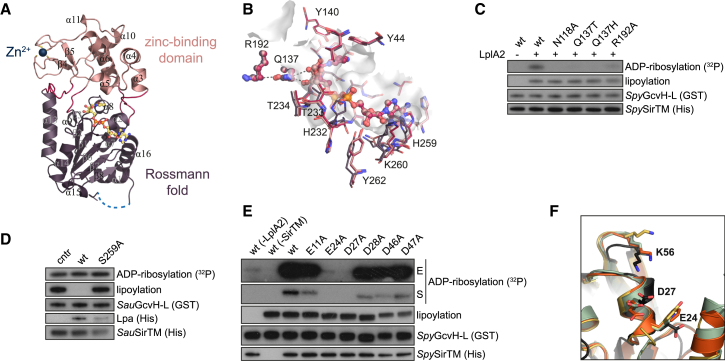
Structural Insights into ADP-Ribosylation by *Spy*SirTM (A) Ribbon representation of the overall structure of *Spy*SirTM in complex with NAD^+^ (yellow). The structure follows a typical sirtuin fold comprised of a Rossmann fold domain (purple) and a small bipartite zinc-coordinating domain (light red) tethered together by four loop regions (red). The zinc ion is indicated in blue and the unresolved loop region as a dotted line (light blue). (B) Comparison of the binding sites of SirTM Apo (purple) in complex with ADPr (light red) or NAD^+^ (red). The view corresponds to (A). (C) The activity of selected *Spy*SirTM mutants was tested in a MARylation assay. *Spy*SirTM WT in the presence and absence of GcvH-L lipoylation were used as controls. For detailed information about the residues see Figure S1 and Table S1. (D) Delipoylation assays were performed with in vivo lipoylated *Sau*GcvH-L (see also Figure 2C). The protein was MARylated with *Sau*SirTM and subsequently delipoylation was performed using WT or catalytically impaired (S259A) lipoamidase. (E) MARylation assays were performed using selected GcvH-L mutants. To distinguish the effects of E24A and D27A short (S) and extended (E) autoradiographic exposures are shown. (F) Comparison of residues involved in lipoyl attachment (K56) and MARylation (E24 and D27). *Spy*GcvH-L structure is shown in black and canonical GcvH of cattle (PDB: 3KLR), pea (PDB: 1DMX), and *M. tuberculosis* (PDB: 3HGB) in orange, green, and yellow, respectively. Residue numbers for GcvH-L are given. See also Figures S5 and S6.

To gain further insight into the SirTM catalyzed ADP-ribosylation, we determined the crystal structure of *Spy*SirTM, both in the ligand-free form as well as bound to ADPr or NAD^+^. The overall fold for these three structures is identical with a low root-mean-square deviation (RMSD) (0.29–0.31 Å). The models contain residues 3–292 (residues 7–10 are not visible in the electron density maps) and follow a typical sirtuin fold comprised of a large Rossmann fold and a small bipartite zinc coordinating domain (Figure 5A; Table 2). *Spy*SirTM is closest in structure to sirtuins from *Thermotoga maritima* (*Tm*Sir2, PDB: 2H4F) and *Saccharomyces cerevisiae* (HST2, PDB: 1SZC) (Figures S5A and S5B).

The ligand-*Spy*SirTM complexes reveal that the general mode of NAD^+^ binding to *Spy*SirTM is similar to that observed in other sirtuins (Yuan and Marmorstein, 2012) (Figures S5A and S5B). No major conformational changes are observed upon binding of either ADPr or NAD^+^, with the exception of slight readjustment of the loop region between β6 and α14 as well as following β7 (Figure 5B). This movement allows the highly conserved Asn258 to interact with the 2′- and 3′-OH groups of the adenine ribose, while the imidazole group of the relatively poorly conserved His259 rotates by ∼110° to allow stacking against the adenine moiety. In addition, the pyrophosphate is coordinated by the class-specific GVGx[NT]TP motif (residues 229–235), found in the β6–α14 loop.

The amide moiety of nicotinamide is bound by direct and water-mediated polar contacts with Ala34, Phe42, Ala119, and Asp120, thus positioning the nicotinamide and the distal ribose in a conformation similar to that observed for *Tm*Sir2 (Figure S5B). The conserved Asn118 coordinates a highly ordered water molecule, which was proposed to facilitate the initial hydrolysis of nicotinamide from NAD^+^ (Zhao et al., 2004). On the α-face of the ribose the 3′-OH group interacts with the absolutely conserved residue Gln137, which replaces the catalytic histidine found in all other sirtuin classes. Gln137 makes further contact with the N^δ^ atom of the similarly conserved residue Arg192 (Figure 5B). Together, these interactions appear to be necessary for the correct positioning of the nicotinamide ribose, and by extension, the GcvH-L substrate. To assess the importance of these residues, we performed MARylation assays with WT and mutant *Spy*SirTM. While WT *Spy*SirTM modified *Spy*GcvH-L, a mutation of Asn118, Gln137, or Arg192 dramatically decreased the catalytic activity (Figure 5C). Interestingly, substitution of Gln137 with the general base histidine, found in other sirtuins, also leads to a complete loss of activity, suggestive of a distinct catalytic mechanism for class M sirtuins.

### Relationship between MARylation and Lipoylation Sites on GcvH-L

To gain further insight into the requirements for the SirTM-mediated ADP-ribosylation, we characterized the ADPr attachment side on the GcvH-L. Since the lipoyl group contains a reactive 1,2-dithiolan moiety that, hypothetically, could be modified in a manner similar to cysteines, we incubated doubly modified *Sau*GcvH-L with WT and catalytically inactive Lpa to remove the lipoyl moiety (Figure 5D). While WT Lpa removed the lipoylation, *Sau*GcvH-L remained radiolabeled, thus indicating that the lipoyl moiety is not the ADPr attachment site.

Next, we tested whether Macro could remove ADP-ribosylation from acidic residues as shown for many of the characterized macrodomain enzymes using MARylated human PARP1 and PARP3 proteins as model substrates (Sharifi et al., 2013; Slade et al., 2011). In these assays, both operonal macrodomains showed comparable activity to human MacroD1 suggesting that they act on acidic residues (Figure S5C). Accordingly, we performed a mutagenesis analysis of highly conserved aspartates and glutamates within GcvH-L (Figures 5E, S6A, and S6B). All tested mutations showed robust lipoylation, but importantly E24A and D27A exhibited near and total loss of modification. Only extended exposure revealed slight ADP-ribosylation of E24A, whereas no ADPr incorporation could be detected for D27A. These findings suggest that Asp27 is the likely ADPr attachment side, but further indicates involvement of Glu24 in the ADP-ribosylation mechanism. It is worth noting, that the presence of an acidic residue in position of Asp27 is absolutely conserved in pathogenic GcvH-L proteins, but it is also common among canonical GcvH proteins. On the other hand, Glu24 is only present in GcvH-L homologs (Figures 5F, S5D, and S6B).

To better understand the spatial relationship between ADP-ribosylation and lipoylation attachment sites, we determined the crystal structure of *Spy*GcvH-L. The structure contains residues 1–110 and is comprised of a barrel-sandwich hybrid formed by two β sheets flanked by short α helices (Table 2). In comparison with previously solved H-proteins (RMSDs for all C^α^ atoms ranging from 0.575–1.14 Å), the most striking differences observed in *Spy*GcvH-L are (1) the position of the β4/β5 loop (residues 38–40), which diverges from the consensus path by ∼6 Å as a consequence of the presence of Arg72 in the β8 strand (a position usually occupied by Val or Ile); and (2) the absence of a C-terminal α helix (Figure S5D). Intriguingly, the missing α helix, which is held in place by electrostatic contacts between four conserved residues in the canonical GcvHs, would occlude residues implicated in ADP-ribose attachment in *Spy*GcvH-L (Glu24 and Asp27) (Figures S5E and S6B). The absence of the steric hindrance imposed by this α helix may be a prerequisite for the GcvH-L/SirTM interaction and thus could account for the high substrate specificity of SirTMs.

### SirTM Function Is Linked to Redox Response

**Figure 6:**
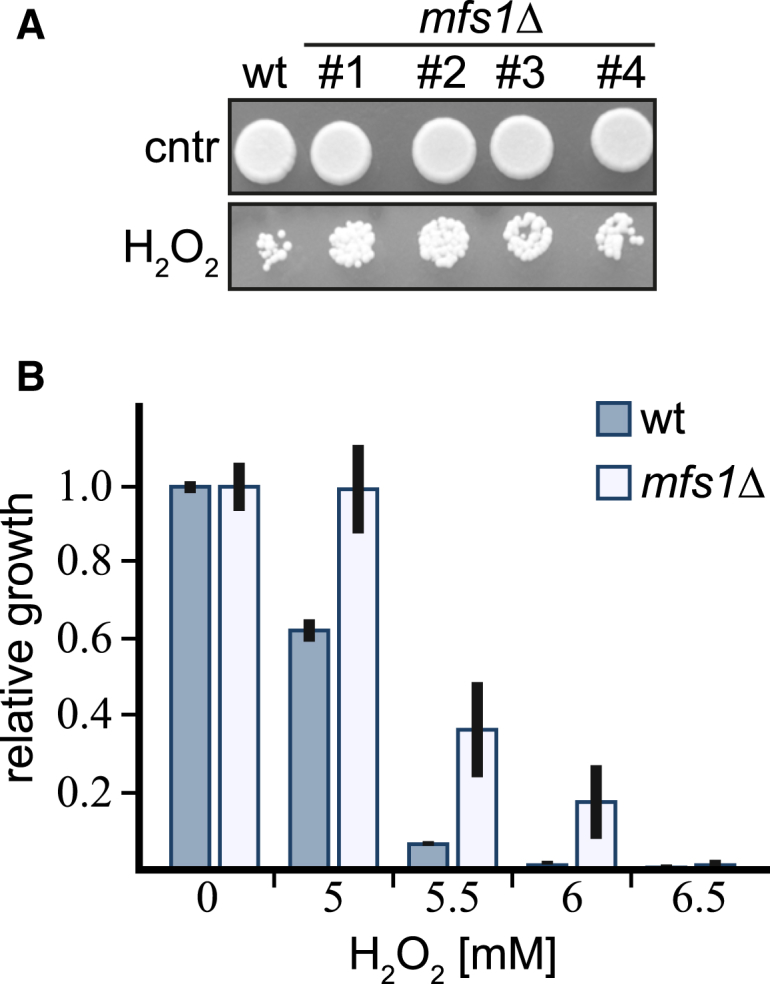
SirTM Function Is Linked to Oxidative Stress Responses (A) Wild-type *C. albicans* and *mfs1Δ* mutants were inoculated onto plates with or without 5 mM H_2_O_2_ and growth assayed after 2 days of incubation at 30°C. Four independent *mfs1* homozygous deletion clones (labeled #1–#4) were tested. Moderate protection from oxidative stress was observed for all four mutant clones. (B) The extent of growth inhibition by H_2_O_2_ was quantified in 96-well plate-based broth assays (see Supplemental Experimental Procedures). Following a 24 hr incubation at 37°C, percentage of growth was calculated relative to control (no H_2_O_2_). The experiment was performed independently twice, in biological duplicates (two independent colonies per strain). A similar trend was observed in both experiments. Shown are averages of growth inhibition ± SD for one of the experiments. See also Figure S7.

We observed that the operonal oxidoreductase *Spy*OYE shows robust, strictly lipoylation-dependent interaction with GcvH-L, as judged by in vitro pull-down assays (Figure 4H). The observation of a direct interaction with an oxidoreductase prompted us to further investigate whether SirTM activity could be linked to the oxidative stress response in vivo. For this, we utilized the pathogenic fungus *Candida albicans* as a model system and created a homozygous deletion strain of the gene encoding the SirTM homolog (*mfs1*, *orf19.2285*) (Figure S7). Upon inactivation of Mfs1 activity, fungal growth improved in the presence of high doses of hydrogen peroxide, as assayed on agar plates (Figure 6A). Improved growth was observed in four independent *mfs1Δ* clones (Figure 6A), and the same trend was observed when we analyzed growth in response to oxidative stress in suspension cultures (Figure 6B). This finding suggests that Mfs1 modulates the oxidative stress response in *C. albicans*.

## Discussion

In the present study, we have identified a distinct class of sirtuins (SirTMs). This class is highly conserved and occurs either as part of a bacterial operon or, in fungi, as a macrodomain-SirTM fusion enzyme.

### SirTMs: Not Deacylases, but ADP-Ribosyl Transferases

Our results show that SirTMs do not possess any appreciable deacylation activity against a number of different endogenous and host-derived substrates (Figures 3 and S3). In accordance with the functional analysis, our sequence and structural analysis revealed the absence of a catalytic histidine residue, crucial for the proposed deacylase mechanism and found in all other hitherto described sirtuins (Sauve, 2010; Yuan and Marmorstein, 2012) and that may explain the apparent absence of deacylation activity in SirTMs. On the other hand, our data show a robust MARylation activity by SirTMs that is highly specific for the GcvH-L, a protein encoded by the same operon. Furthermore, the SirTM-dependent ADP-ribosylation is specifically and efficiently reversed by the pathogenic macrodomain proteins. Collectively, our data argue for protein ADP-ribosyl transferase as the primary activity of SirTMs.

### Operon Proteins in the Response to Oxidative Stress

The data presented here strongly support the notion that ADP-ribosylation of GcvH-L is dependent on its prior lipoylation. The operon-encoded LplA2/GcvH-L pair is only distantly related to the canonical GCS, but nonetheless, the catalytic and the lipoate attachment residues are conserved in the proteins from pathogenic species. While *S. pyogenes* naturally lacks a canonical GCS, *S. aureus* possesses a complete GCS as well as the operon-encoded LplA2/GcvH-L pair (Spalding and Prigge, 2010 and this study). Interestingly, our analysis showed that the canonical *Sau*LplA1 can modify both GcvH proteins, while *Sau*LplA2 appears to have selectivity toward GcvH-L (Figure S2C). This may indicate that lipoylation of GcvH-L is the preferred target for LA attachment under conditions of operon activation.

Transcriptome and proteome analysis revealed that activation of this operon occurs under conditions of oxidative stress (Nobre and Saraiva, 2013; Palazzolo-Ballance et al., 2008; Surmann et al., 2014), consistent with an involvement in the oxidative stress response. This is further supported by several observations: (1) LA was shown to possess antioxidant properties in models of oxidative stress (Packer et al., 1995), (2) it was reported that *Mycobacterium tuberculosis* utilizes two components of the α-ketoacid dehydrogenase complex in a lipoyl-dependent defense against the oxidative immune response of the host (Bryk et al., 2002), and (3) the operon is genetically associated with the putative oxidoreductases LLM and OYE. Both enzymes are as yet uncharacterized. However, their families have been implicated in detoxification of ROS (Chaiyen et al., 2012; Odat et al., 2007), and we show that the oxidoreductase OYE directly interacts with lipoylated GcvH-L. These findings suggest that GcvH-L could act as carrier protein for the ROS scavenging lipoyl moiety and/or as a substrate for oxidoreductases. Evidence obtained from the fungal pathogen *C. albicans* in this study points toward an involvement of SirTMs in the oxidative stress response. Accordingly, in *C. albicans* the expression of *mfs1* is very low under standard growth conditions, and the gene is only expressed to a considerable degree upon oxidative stress either chemically induced (Enjalbert et al., 2009) or by exposure to human blood (Fradin et al., 2003). Collectively, these results are consistent with a primary cellular function of Mfs1 when cells encounter oxidative stress, such as upon host interactions. In line with a specific function in host-pathogen interactions, engulfment of *S. aureus* by host (human) cells led to an induction of the macrodomain encoded in the SirTM operon (Surmann et al., 2014).

Combining these observations together with the high prevalence of the SirTM operon in known pathogenic microorganisms and the importance of lipoate scavenging for microbial pathogenesis (Spalding and Prigge, 2010), we propose that the SirTM operon or SirTM-macrodomain fusion proteins in eukaryotic microbes modulate the response of microbial pathogens to oxidative stress and host-pathogen interactions.

### Crosstalk between Lipoylation and ADP-Ribosylation

Here, we present the direct evidence for a connection between protein lipoylation and ADP-ribosylation. Collectively our data suggest that lipoylation of GcvH-L is a prerequisite for its MARylation. Since ADP-ribosylation is a known modification influencing protein function and interactions (Barkauskaite et al., 2013), it is interesting to speculate that the MARylation state of GcvH-L might regulate the availability of the lipoyl moiety for redox reactions. This idea is supported by our finding that the ADPr attachment site is in close structural proximity to the lipoylation site. Moreover, the specific upregulation of the operonal macrodomain in *S. aureus* after internalization into human cells (Surmann et al., 2014) indicates that under these conditions de-MARylation of GcvH-L prevails. Collectively, these data suggest that the ADP-ribosylation inhibits the interaction of the oxidoreductase with GcvH-L when it is not required. In other words, ADP-ribosylation of GcvH-L might be acting to keep the response “off” under non-stress conditions.

## Experimental Procedures

The experimental procedures are described in detail in the Supplemental Information. These procedures include plasmid construction, protein expression and purification, structure determination, interaction and enzymatic activity assays, cell culture condition, as well as phylogenetic analysis. Below is a simplified description of the major experimental procedures. The amino acid sequences of the operon proteins can be accessed through UniProtKB: under Q99WQ2, P67343, Q99WQ0, and Q99WP9 (*S. aureus*) as well as A2REC0, A2REC1, A2REC2, and A2REC3 (*S. pygones*).

### Protein Expression and Purification

Recombinant proteins were expressed in Rosetta (DE3) cells and affinity purified either by Ni^2+^-NTA chromatography (QIAGEN) or using glutathione Sepharose 4B (GE Life Sciences). All proteins were dialyzed over night against protein buffer (50 mM TrisHCl [pH 8], 200 mM NaCl, 1 mM DTT) (see Supplemental Information for further details). Protein purity was assessed by SDS-PAGE (Figure S2A).

### Enzymatic Assays

Lipoylation reactions were carried out using 1 μM LplA and 2 μM target protein. Reactions were incubated for 30 min at 30°C before analysis by immunoblot or further processing. Control reactions were carried out in the absence of LplA.

Deacetylation of p53-derived peptide was tested with the SIRT-Glo assay kit (Promega) according to the manufacturer’s recommendations using 6 nM sirtuin per reaction. Deacetylation of synthetic penta-acetylated histone H3 (Active Motif) was carried out as described earlier (Rack et al., 2014).

Delipoylation of in vitro and in vivo lipoylated GcvH-L was carried out in delipoylation buffer (50 mM TrisHCl [pH 8], 200 mM NaCl, 10 mM MgCl_2_, 1 mM DTT) using 1 μM recombinant sirtuin, 1 μM GcvH1, and 1 mM NAD^+^. Reactions were incubated at 30°C for 2 hr.

ADP-ribosylation reactions were carried out using 1 μM sirtuin, 1 μM target protein, 2 μCi ^32^P-NAD^+^, and 5 μM unlabeled NAD^+^. Reactions were incubated for 60 min at 30°C before analysis by immunoblot or further processing. De-ADP-ribosylation assay was carried out using 1 μM radiolabeled GcvH-L and 1 μM macrodomain protein. The reactions were incubated for 1 hr at 30°C.

For further details regarding enzymatic assays, see Supplemental Information.

### Crystallization

Recombinant proteins were concentrated to a concentration of ∼14 mg/ml. Crystals were obtained by hanging-drop vapor diffusion at 293 K by mixing 120 nl protein solution with 30 nl seed stock and 150 nl precipitant. For the ADPr and NAD^+^ complexes, native crystals were soaked with 1 mM ligand in mother liquor for 20 hr and 10 min, respectively. The crystals were cryoprotected in a solution of mother liquor plus 20% ethylene glycol and vitrified by submersion in liquid nitrogen for data collection.

A single *Spy*GcvH-L crystal grew at 293K using the sitting-drop vapor diffusion. The crystal was cryoprotected in a solution of precipitant plus 15% PEG400 (see Supplemental Information for further details).

### X-Ray Data Collection, Processing, Structure Determination, Refinement, and Analysis

X-ray diffraction data were collected using synchrotron radiation (Table 2). The CCP4 software suite (Winn et al., 2011) was used to process all datasets, including integration, scaling, molecular replacement, density modification, automated model building, and structure refinement. COOT (Emsley and Cowtan, 2004) was used for manual model building and autoSHARP (Vonrhein et al., 2007) for locating the Se atoms in the Apo selenomethionine derivate as well as for refinement and initial phase calculation. The processing, phasing, and final refinement statistics are presented in Table 2 (see Supplemental Information for details).

All structural alignment were generated in COOT and figures prepared with PyMOL (Molecular Graphics System, Version 1.3 Schrödinger, LLC) using DSSP (Joosten et al., 2011) for secondary structure assignment.

## Author Contributions

J.G.M.R., R.M., R.K., and E.B. purified proteins and performed biochemical studies. E.B. and A.A. performed crystallography studies. Y.Q., D.C., R.M., A.Y.P., and A.T. performed in vivo studies. M.O., O.L., and I.M. performed supporting studies. J.G.M.R., D.L., and I.A. analyzed data. J.G.M.R. and I.A. wrote the manuscript.

## Figures and Tables

**Table 1:** Comparison between the Lipoylation and Biotinylation Systems of *S. aureus* and *S. pyogenes*

		*S. aureus*		*S. pyogenes*	
		Protein	Accession^a^	Protein	Accession^a^
Lipoylation^b^	Lipoate scavenging	LplA1	CAG42736	LplA1	CAM30328
		LplA2^c^	CAG42075	LplA2^c^	CAM30198
		LplA3	CAG43266	–	–
		LplA4	CAG42323	–	–
	Lipoyl-carrier proteins	–	–	AcoC	CAM30333
		–	–	AcoL	CAM30332
		DBT	CAG43237	–	–
		DLAT	CAG42804	–	–
		DLST	CAG43130	–	–
		GcvH	CAG42548	–	–
		GcvH-L^c^	CAG42072	GcvH-L^c^	CAM30195
Biotinylation^d^	Biotin scavenging	BPL	BAB57618	BPL	CAM30079
	Biotin-carrier proteins	BCCP (ACC)	BAB57689	BCCP (ACC)	CAM29699
		–	–	BCCP (OAD)	CAM30229
		PC	BAB57276	–	–

ACC, acetyl-CoA carboxylase; AcoC, dihydrolipoamide acetyltransferase (AoDH-E2); AcoL, dihydrolipoamide dehydrogenase (AoDH-E3); BCCP, biotin carboxyl carrier protein; BPL, biotin protein ligase; DBT, dihydrolipoamide branched chain transacylase E2 (BCDH-E2); DLAT, dihydrolipoamide *S*-acetyltransferase (PDH-E2); DLST, dihydrolipoamide *S*-succinyltransferase (KDH-E2); OAD, oxaloacetate dehydrogenase; PC, pyruvate carboxylase.

a GenBank accession numbers.

b Based on Spalding and Prigge (2010).

c Operon-encoded proteins.

d Based on Fall (1979).

**Table 2:** Crystallographic Data Collection and Refinement

	Value(s)^a^ for			
	*Spy*SirTM			*Spy*GcvH-L
	Apo	ADPr Complex	NAD^+^ Complex	Apo
**Data Collection Statistics**				

Wavelength (Å)/beamline	0.92000/I04-1	0.97625/I03	0.97625/I03	0.92000/I04-1
Detector	Pilatus 2M	Pilatus3 6M	Pilatus3 6M	Pilatus 2M
Space group	*P*1	*P*1	*P*1	*P*3_1_ 2 1

**Unit Cell**				

a (Å)	34.16	33.94	33.88	64.12
b (Å)	41.51	41.52	41.48	64.12
c (Å)	51.23	51.25	51.26	73.04
α (°)	99.87	99.76	99.55	90.00
β (°)	94.63	93.40	93.61	90.00
γ (°)	90.77	92.66	92.98	120.00
Content of asymmetric unit	1	1	1	1

**Resolution (Å)**	**34.81–1.54**	**29.35–1.90**	**40.82–2.03**	**55.53–1.50**

	(1.58–1.54)	(1.94–1.90)	(2.08–2.03)	(1.53–1.50)
R_sym_ (%)^b^	5.9 (54.8)	7.4 (32.6)	14.6 (60.1)	3.9 (57.0)
I/σ(I)	10.6 (2.0)	8.0 (4.5)	4.8 (1.9)	27.1 (2.9)
Redundancy	3.8 (3.8)	2.4 (2.2)	2.3 (2.3)	8.5 (6.2)
Completeness (%)	88.3 (82.8)	92.1 (88.9)	97.2 (95.2)	99.3 (93.0)
Number of unique reflections	36,095 (2,501)	19,940 (1,296)	17,229 (1,263)	28,124 (1,293)

**Refinement Statistics**				

R_cryst_ (%)^c^	15.0	16.3	17.6	15.4
R_free_ (%)^d^	20.0	21.6	23.3	17.1
RMSD bond length (Å)	0.016	0.011	0.008	0.021
RMSD bond angle (°)	1.42	1.45	1.18	1.55

**Average B Factor (Å**^**2**^**)**				

Protein	17.9	13.8	24.6	22.0
Water	32.1	24.6	32.6	39.6
Zn^2+^	18.9	16.9	25.5	
ADPr	–	14.8	–	
NAD^+^	–	–	24.5	
EDO/GLY/ALA/1PE	38.1/50.7/–/–	35.2/39.8/43.8/–	45.4/51.9/–/–	–/–/–/46.8

a Data for the highest resolution shell are given in parentheses.

b R_sym_ = Σ|/− </> |/Σ/, where / is measured density for reflections with indices *hkl*.

c R_cryst_ = Σ||Fobs| − |Fcalc||/Σ|Fobs|.

d R_free_ has the same formula as R_cryst_, except that calculation was made with the structure factors from the test set.

## Accession Numbers

Atomic coordinates and structure factors have been deposited in the Protein Data Bank under ID codes PDB: 5A35, 5A3A, 5A3B, 5A3C.

## Supplemental Information

Document S1. Supplemental Experimental Procedures, Figures S1–S7, and Tables S1 and S2

Document S2. Article plus Supplemental Information
