# Supplementary material for: Identification of a Class of Protein ADP-Ribosylating Sirtuins in Microbial Pathogens
Source: Mol Cell. 2015 Jul 16;59(2):309–20. doi: 10.1016/j.molcel.2015.06.013 (PMC4518038; doi:10.1016/j.molcel.2015.06.013)
Supplement: Document S1. Supplemental Experimental Procedures, Figures S1–S7, and Tables S1 and S2 [file mmc1.pdf]

Molecular Cell, Volume *59*

Supplemental Information

Identification of a Class of Protein ADP-Ribosylating Sirtuins in Microbial Pathogens

Johannes Gregor Matthias Rack, Rosa Morra, Eva Barkauskaite, Rolf Kraehenbuehl, Antonio Ariza, Yue Qu, Mary Ortmayer, Orsolya Leidecker, David R. Cameron, Ivan Matic, Anton Y. Peleg, David Leys, Ana Traven, and Ivan Ahel

Supplemental Figures and Figure Legends

Figure S1. Distinctive features of class M sirtuins (related to Figure 1).

(A) Multiple sequence alignment generated with representative sequences for class M sirtuins. Conservation of sequences is given in grey scale with a cut-off of 30%. The secondary structure elements of *Spy*SirTM are given on top of the alignment. Note that helices α5 and α15 are broken due to gaps in the alignment (dotted lines). Sequence motifs characterizing the novel sirtuin class are given underneath the alignment indicated as follows: Cofactor binding motif GAGxSAx(2)Gx(2)Y, purple triangles; nicotinamide ribose loop T[ST]N[VA]D, blue circles; catalytic residues QG and R, red asterisks; first part of zinc finger Cx(3)C, grey-blue diamonds and β6-α14 loop GVGx[NT]TP, light-red squares (Table S1).

(B) Ribbon-surface representation of *Spy*SirTM bound to NAD^+ (yellow). The sequence motifs representative for the novel sirtuin class are highlighted in different colors (see (A) for coloring description). The position of the unresolved residues is indicated by a dashed line.

(C) Multiple sequence alignment comparing the novel class M sirtuins with the other sirtuin classes: class I (HST2 and SIRT1), class II (SIRT4), class III (SIRT5), class IV (*Atr*Sir2L1 and SIRT7) and class U (*Sau*CobB, *Tb*Sir2 and *Tm*Sir2) (compare also Figure 1A). Secondary structure elements of *Spy*SirTM (yellow) and HST2 (green) are given on top of the alignment and broken elements due to alignment gaps are indicated by dotted lines. Sequence motifs for class M sirtuins are indicated underneath the alignment as in (A). For sequence accession numbers see Table S2.

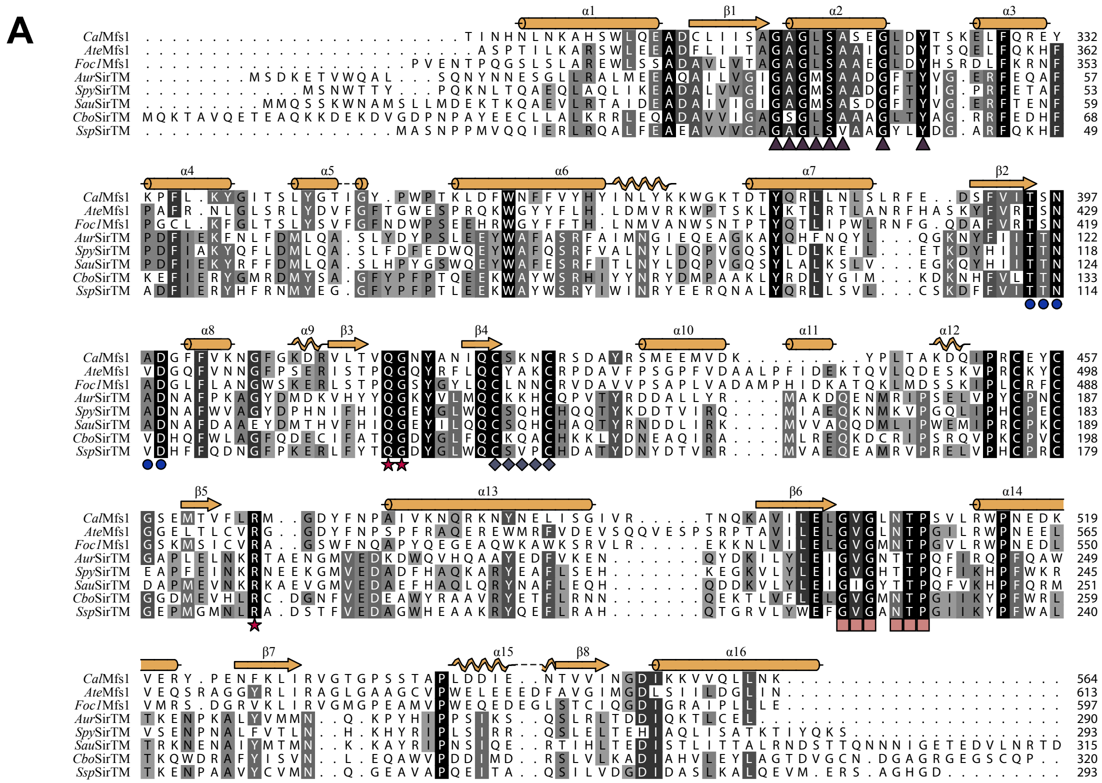
A
CalMfs1
AteMfs1
Foc1Mfs1
AurSirTM
SpySirTM
SauSirTM
CboSirTM
SspSirTM
α1 β1 α2 α3
α4 α5 α6 α7 β2
α8 α9 β3 β4 α10 α11 α12
β5 α13 β6 α14
β7 α15 β8 α16

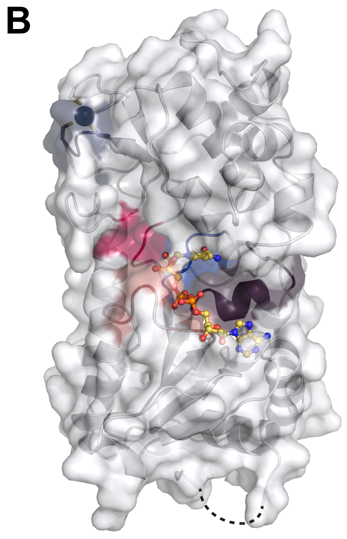
B

Figure S1

C

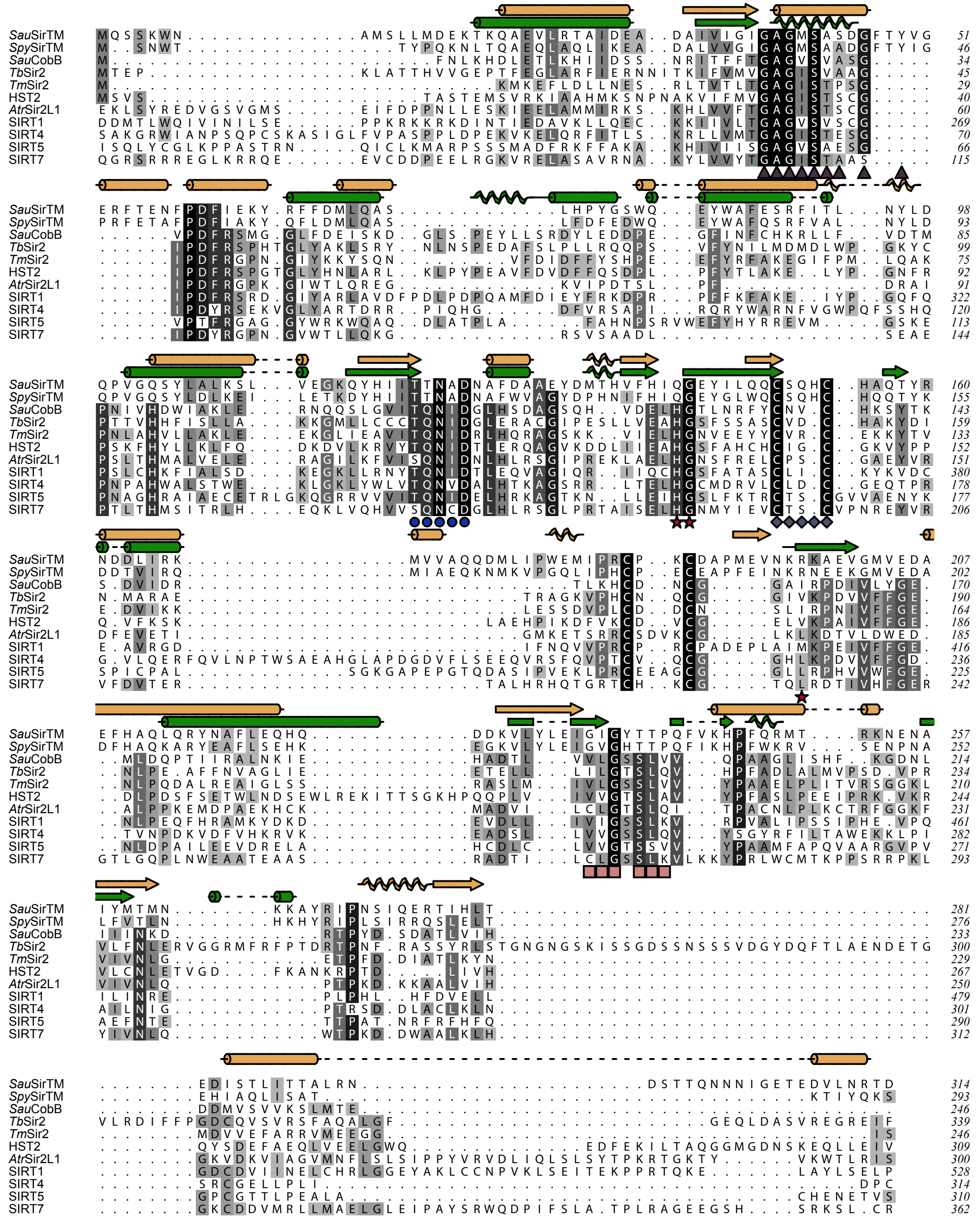
SauSirTM MQSSKWN..............AMSLLMDEKTKQAEVLRTAIDEA..DAIVIGIGAGMSASDGFTYVG 51
SpySirTM M..SNWT...............TYPQKNLTQAEQLAQLIKEA..DALVVGIGAGMSAADGFTYIG 46
SauCobB M.......................FNLKHDLETLKHIIDSS..NRITFFTGAGVSVASG.... 34
TbSir2 MTEP..............KLATTHVVGEPTFEGLARFIERNNITKIFVMVGAGISVAAG.... 45
TmSir2 M..........................KMKEFLDLLNES..RLTVTLTGAGISTPSG.... 29
HST2 MSVS................TASTEMSVRKIAAHMKSNPNAKVIFMVGAGISTSCG.... 40
AtrSir2L1 EKLSYREDVGSVGMS......EIFDPPNLLESKIEELAMMIRKS..KHLVVFTGAGISTSCG.... 60
SIRT1 DDMTLWQIVINILSE......PPKRKKRKDINTIEDAVKLLQEC..KKIIVLTGAGVSVSCG.... 269
SIRT4 SAKGRWIANPSQPCSKASIGLFVPASPPLDPEKVKELQRFITLS..KRLLVMTGAGISTESG.... 70
SIRT5 ISQLYCGLKPPASTRN.....QICLKMARPSSSMADFRKFFAKA..KHIVIISGAGVSAESG.... 66
SIRT7 QGRSRRREGLKRRQE......EVCDDPEELRGKVRELASAVRNA..KYLVVYTGAGISTAAS.... 115
SauSirTM ERFTENFPDFIEKY.RFFDMLQAS..............LHPYGSWQ...EYWAFESRFITL...NYLD 98
SpySirTM PRFETAFPDFIAKY.QFLDMLQAS..............LFDFEDWQ...EYWAFQSRFVAL...NYLD 93
SauCobB ......VPDFRSMG.GLFDEISKD..GLS.PEYLLSRDYLEDDPE...GFINFCHKRLLF....VDTM 85
TbSir2 ......IPDFRSPHTGLYAKLSRY.NLNSPEDAFSLPLLRQQPS...VFYNILMDMDLWP..GKYC 99
TmSir2 ......IPDFRGPN.GIYKKYSQN.........VFDIDFFYSHPE...EFYRFAKEGIFPM...LQAK 75
HST2 ......IPDFRSPGTGLYHNLARL.KLPYPEAVFDVDFFQSDPL...PFYTLAKE..LYP..GNFR 92
AtrSir2L1 ......IPDFRGPK.GIWTLQREG...........KVIPDTSL...PF..........DRAI 91
SIRT1 ......IPDFRSRD.GIYARLAVDFPDLPDPQAMFDIEYFRKDPR...PFFKFAKE..IYP..GQFQ 322
SIRT4 ......IPDYRSEKVGLYARTDRR.PIQHG.....DFVRSAPI...RQRYWARNFVGWPQFSSHQ 120
SIRT5 ......VPTFRGAG.GYWRKWQAQ.DLATPLA.....FAHNPSRVWEFYHYRREVM.....GSKE 113
SIRT7 ......IPDYRGPN.GVWTLLQKG...........RSVSAADL......................SEAE 144
SauSirTM QPVGQSYLALKSL...VEGKQYHIITTNADNAFDAAEYDMTHVFHIQGEYILQQCSQHC..HAQTYR 160
SpySirTM QPVGQSYLDLKEI...LETKDYHIITTNADNAFWVAGYDPHNIFHIQGEYGLWQCSQHC..HQQTYK 155
SauCobB PNIVHDWIAKLE....RNQQSLGVITQNIDGLHSDAGSQH..VDELHGTLNRFYCNV.C..HKSYTK 143
TbSir2 PTTVHHFISLLA....KKGMLLCCCTQNIDGLERACGIPESLLVEAHGSFSSASCVD.C..HAKYDI 159
TmSir2 PNLAHVLLAKLE....EKGLIEAVITQNIDRLHQRAGSKK..VIELHGNVEEYYCVR.C..EKKYTV 133
HST2 PSKFHYLLKLFQ....DKDVLKRVYTQNIDTLERQAGVKDDLIIEAHGSFAHCHCIG.C..GKVYPP 152
AtrSir2L1 PSLTHMALVELE....RAGILKFVISQNIDNLHLRSGIPREKLAELHGNSFRELCPS.C..GAEYVR 151
SIRT1 PSLCHKFIALSD....KEGKLLRNYTQNIDTLEQVAGIQR..IIQCHGSFATASCLI.C..KYKVDC 380
SIRT4 PNPAHWALSTWE....KLGKLYWLVTQNVDALHTKAGSRR..LTELHGCMDRVLCLD.C..GEQTPR 178
SIRT5 PNAGHRAIAECETRLGKQGRRVVVITQNIDELHRKAGTKN..LLEIHGSLFKTRCTS.CGVVAENYK 177
SIRT7 PTLTHMSITRLH....EQKLVQHVVSQNCDGLHLRSGLPRTAISELHGNMYIEVCTS.CVPNREYVR 206
SauSirTM NDDLIRK..............MVVAQQDMLIPWEMIPRCP..KCDAPMEVNKRKAEVGMVEDA 207
SpySirTM DDTVIRQ..............MIAEQKNMKVPGQLIPHCP..ECEAPFEINKRNEEKGMVEDA 202
SauCobB S.DVIDR.........................TLKHCD..NCG....GAIRPDIVLYGE 170
TbSir2 N.MARAE...................TRAGKVPHCN..QCG....GIVKPDVVFFGE 190
TmSir2 E.DVIKK.....................LESSDVPLCD..DCN....SLIRPNIVFFGE 164
HST2 Q.VFKSK................LAEHPIKDFVKCD..VCG....ELVKPAIVFFGE 186
AtrSir2L1 DFEVETI..................GMKETSRRCSDVKCG....LKLKDTVLDWED 185
SIRT1 E.AVRGD.....................IFNQVVPRCP..RCPADEPLAIMKPEIVFFGE 416
SIRT4 G.VLQERFQVLNPTWSAEAHGLAPDGDVFLSEEQVRSFQVPTCV..QCG....GHLKPDVVFFGD 236
SIRT5 SPICPAL..........SGKGAPEPGTQDASIPVEKLPRCEEAGCG....GLLRPHVVWFGE 225
SIRT7 VFDVTER...................TALHRHQTGRTCH..KCG....TQLRDTIVHFGER 242
SauSirTM EFHAQLQRYNAFLEQHQ.........DDKVLYLEIGIGYTTPQFVKHPFQRMT....RKNENA 257
SpySirTM DFHAQKARYEAFLSEHK.........EGKVLYLEIGVGHTTPQFIKHPFWKRV....SENPNA 252
SauCobB ..MLDQPTIIRALNKIE.........HADTL...VVLGSSLVV...QPAAGLISHF..KGDNL 214
TbSir2 ..NLPE.AFFNVAGLIE.........ETELL...LILGTSLQV...HPFADLALMVPSD.VPR 234
TmSir2 ..NLPQDALREAIGLSS.........RASLM...IVLGSSLVV...YPAAELPLITVRSGGKL 210
HST2 ..DLPDSFSETWLNDSEWLREKITTSGKHPQQPLV...IVVGTSLAV...YPFASLPEEIPRK.VKR 244
AtrSir2L1 ..ALPPKEMDPAEKHCK.........MADVI...LCLGTSLQI...TPACNLPLKCTRFGGKF 231
SIRT1 ..NLPEQFHRAMKYDKD.........EVDLL...IVIGSSLKV...RPVALIPSSIPHE.VPQ 461
SIRT4 ..TVNPDKVDFVHKRVK.........EADSL...LVVGSSLQV...YSGYRFILTAWEKKLPI 282
SIRT5 ..NLDPAILEEVDRELA.........HCDLC...LVVGTSSVV...YPAAMFAPQVAARGVPV 271
SIRT7 GTLGQPLNWEAATEAAS.........RADTI...LCLGSSLKVLKKYPRLWCMTKPPSRRPKL 293
SauSirTM IYMTMN.......KKAYRIPNSIQERTIHLT.................. 281
SpySirTM LFVTLN.......HKHYRIPLSIRRQSLELT.................. 276
SauCobB IIINKD..........RTPYD.SDATLVIH.................. 233
TbSir2 VLFNLERVGGRMFRFPTDRTPNF.RASSYRLSTGNGNGSKISSGDSSNSSSVDGYDQFTLAENDETG 300
TmSir2 VIVNLG..........ETPFD.DIATLKYN.................. 229
HST2 VLCNLETVGD....FKANKRPTD.....LIVH.................. 267
AtrSir2L1 VIVNLQ..........PTPKD.KKAALVIH.................. 250
SIRT1 ILINRE..........PLPHL..HFDVELL.................. 479
SIRT4 AILNIG..........PTRSD.DLACLKLN.................. 301
SIRT5 AEFNTE..........TTPAT.NRFRFHFQ.................. 290
SIRT7 YIVNLQ..........WTPKD.DWAALKLH.................. 312
SauSirTM ........EDISTLITTALRN..............DSTTQNNNIGETEDVLNRTD 314
SpySirTM ....EHIAQLISAT...........................KTIYQKS 293
SauCobB ........DDMVSVVKSLMTE........................ 246
TbSir2 VLRDIFFPGDCQVSVRSFAQALGF...........GEQLDASVREGREIF 339
TmSir2 ........MDVVEFARRVMEEGG....................IS 246
HST2 ........QYSDEFAEQLVEELGWQ.......EDFEKILTAQGGMGDNSKEQLLEIV 309
AtrSir2L1 ........GKVDKVIAGVMNFLSLSIPPYVRVDLIQLSLSYTPKRTGKTY......VKWTLRIS 300
SIRT1 ........GDCDVIINELCHRLGGEYAKLCCNPVKLSEITEKPPRTQKE.....LAYLSELP 528
SIRT4 ....SRCGELLPLI.........................DPC 314
SIRT5 ........GPCGTTLPEALA.................CHENETVS 310
SIRT7 ........GKCDDVMRLLMAELGLEIPAYSRWQDPIFSLA.TPLRAGEEGSH...SRKSL.CR 362

Figure S1C

Figure S2. Specificity of GcvH-L lipoylation (related to Figure 2).

(A) Coomassie stained SDS-PAGE visualizing the purity of the operonal wild type proteins (4 µg protein per lane) after single affinity purification as used in biochemical assays. Mutant proteins were co-purified and of comparable purity.

(B) The lipoylation assay was performed with GST-tagged operon and associated proteins (indicated on top) derived from *S. aureus*. Proteins were incubated in the presence or absence of lipoic acid and the lipoylation status analyzed by IB. Note, lipoylated protein band in the LLM samples correspond in size to co-purified *E. coli* dihydrolipoamide *S*-succinyltransferase (DLST).

(C) Specificity of the lipoylation reactions of *S. aureus* LplA1, 2 and *Eco*LplA was assets using the canonical GcvHs and GcvH-L as substrates. Control reactions were carried out in the absence of LplA.

(D) *In vivo* lipoylated *Sau*GcvH-L (see Figure 2C) was incubated in the absence or presence of Lpa and the lipoylation status analyzed by IB.

(E) The ability of *S. aureus* LplA2 to utilize lipoic acid and lipoamide as substrates was tested by performing lipoylation reactions with increasing amounts of substrates (0.24, 2.4, 24 and 240 µM, respectively). Control reaction were carried out in the absence of LplA2 and substrate incubation was analyzed by IB.

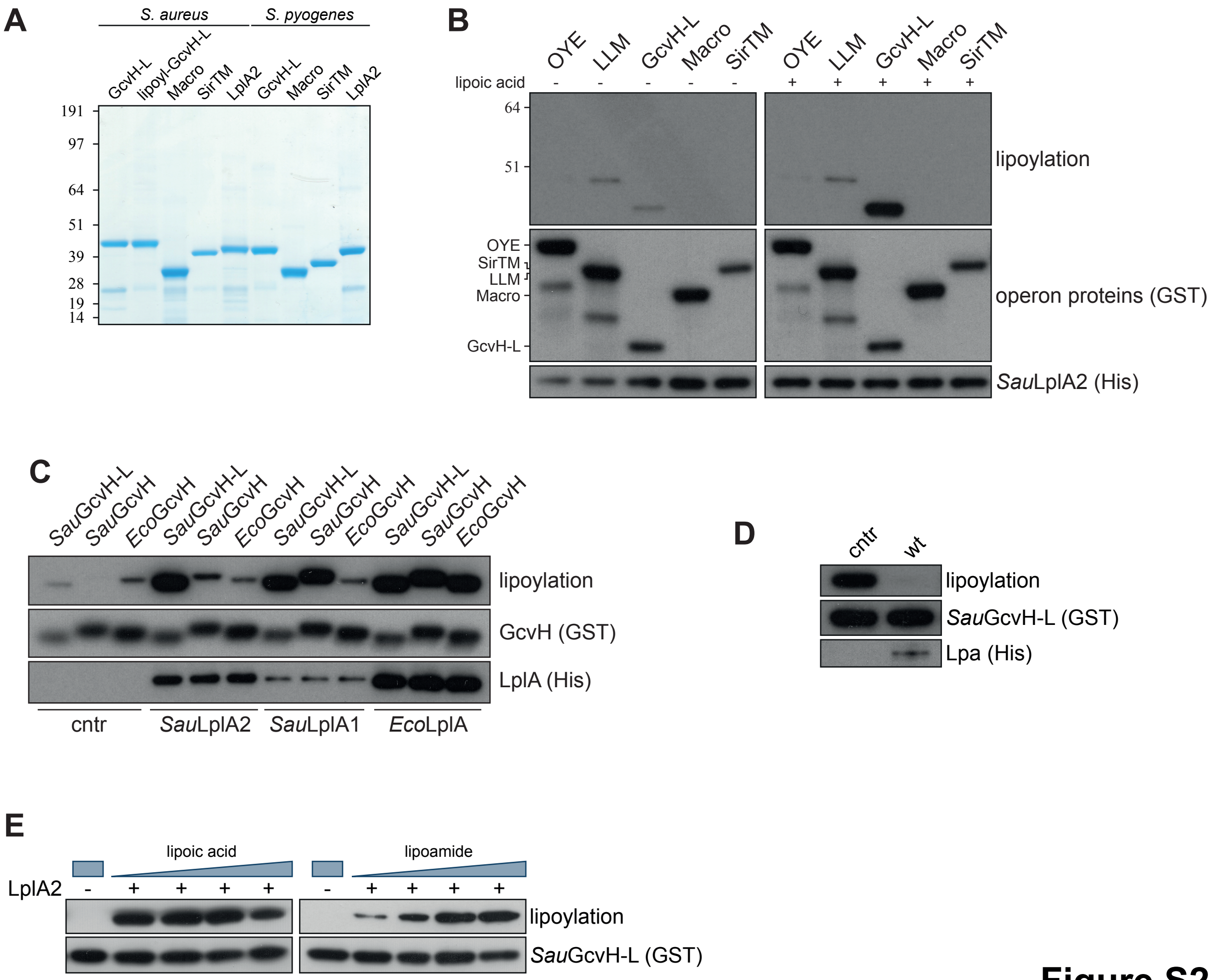

A
S. aureus
S. pyogenes
GcvH-L
lipoyl-GcvH-L
Macro
SirTM
LplA2
GcvH-L
Macro
SirTM
LplA2
191
97
64
51
39
28
19
14
B
OYE
LLM
GcvH-L
Macro
SirTM
lipoic acid
64
51
lipoylation
OYE
SirTM
LLM
Macro
GcvH-L
operon proteins (GST)
SauLplA2 (His)
C
SauGcvH-L
SauGcvH
EcoGcvH
lipoylation
GcvH (GST)
LplA (His)
cntr
SauLplA2
SauLplA1
EcoLplA
D
cntr
wt
lipoylation
SauGcvH-L (GST)
Lpa (His)
E
lipoic acid
lipoamide
LplA2
lipoylation
SauGcvH-L (GST)

Figure S2

Figure S3. SirTMs lack deacylase activity (related to Figure 3 and 4).

(A) De-lipoylation and -biotinylation assay performed on *E. coli* BL21(DE3)Δ*CobB* cell extracts. For lipoylation modified DLAT and DLST could be detected, whereas BCCP could be identified as biotinylated.

(B) Desuccinylating activities of *Sau*SirTM and *Spy*SirTM against a succinyl-peptide were assessed using a fluorogenic SIRT5 assay (BPS Bioscience). Nicotinamide inhibition of SIRT5 and catalytic mutants were used as controls as indicated. Data are background corrected means ± SD of triplicate measurements.

(C) Desuccinylation assay performed with non-enzymatically succinylated histone octamers.

(D) Deacetylase activity of *Tb*Sir2 against p53 derived peptide was assets using the SIRT-Glo assay (Promega). A catalytic inactive mutant (H142Y) and nicotinamide inhibition were used as controls as indicated. Data are background corrected means ± SD of triplicate measurements.

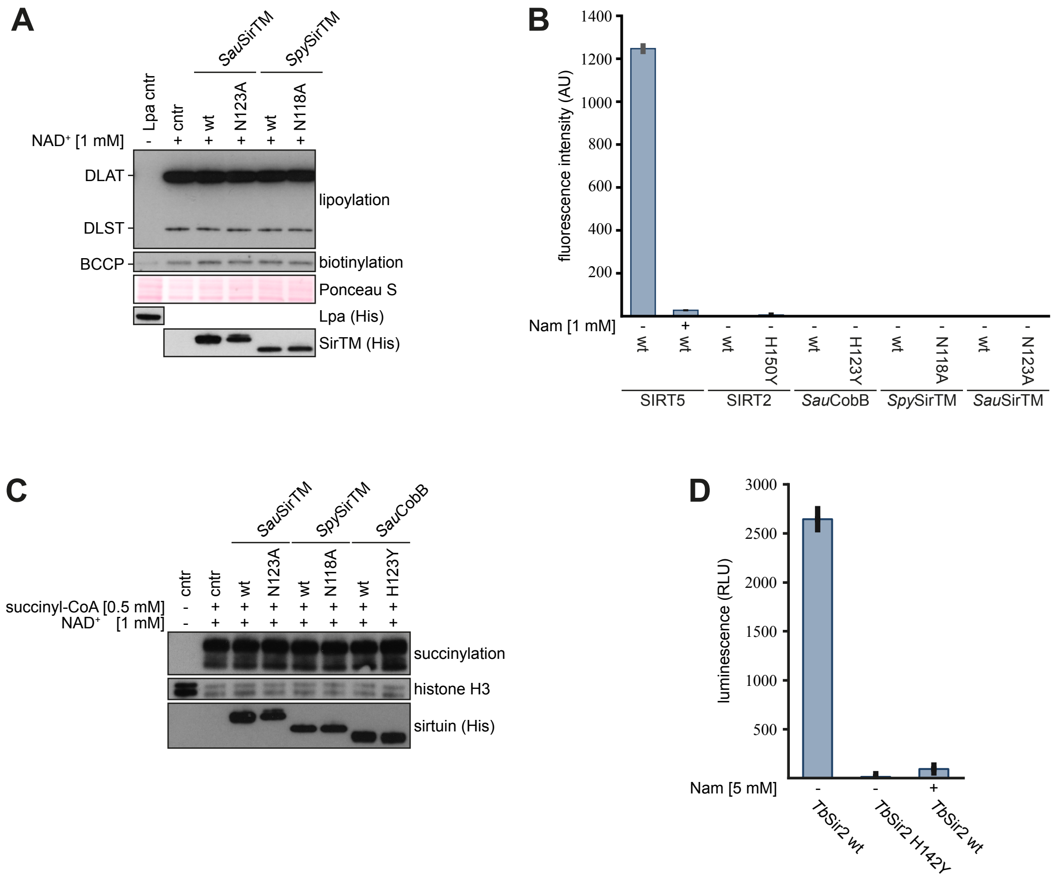

A
SauSirTM
SpySirTM
Lpa cntr
cntr
wt
N123A
wt
N118A
NAD⁺ [1 mM]
DLAT
DLST
lipoylation
BCCP
biotinylation
Ponceau S
Lpa (His)
SirTM (His)
B
fluorescence intensity (AU)
1400
1200
1000
800
600
400
200
Nam [1 mM]
wt
H150Y
H123Y
N118A
N123A
SIRT5
SIRT2
SauCobB
SpySirTM
SauSirTM
C
SauCobB
H123Y
succinyl-CoA [0.5 mM]
NAD⁺ [1 mM]
succinylation
histone H3
sirtuin (His)
D
luminescence (RLU)
3000
2500
2000
1500
1000
500
Nam [5 mM]
TbSir2 wt
TbSir2 H142Y

Figure S3

Figure S4. The specific ADP-ribosylation of GcvH-L (related to Figure 4).

(A) ADP-ribosylation of GcvH-L can be reversed by treatment with the human phosphodiesterase NUDT16.

(B) MARylation assay performed in the presence of free lipoic acid or lipoamide. Control reactions were carried out in the absence of substrate as well as in the presence of lipoic acid and LplA2.

(C) MARylation assay performed with *Spy*SirTM and unmodified *Spy*GcvH-L in the presence of unmodified, lipoylated and acetylated peptides with sequences derived from *Spy*GcvH-L and histone H3. Control reaction was carried out in the presence of lipoylated *Spy*GcvH-L and *Spy*LplA2.

(D) MARylation of the peptides utilized in (C) was assessed by TLC. No retention of radiolabel at the origin was observed, which would be expected in case the peptide substrate incorporated the ADPr (compare also Figure 4G). The enzyme present in the assay is indicated on top and the peptide underneath the panel.

(E) MARylation assay performed with unmodified and biotinylated BCCP.

(F) *S. aureus* SirTM and Macro were tested for NAD^+ hydrolase activity by incubation of ^{32}P-NAD^+ with the enzymes under standard MARylation assay conditions (compare Materials and Methods). Human MacroD1 was used as unrelated control. Samples were resolved by TLC and analyzed by autoradiography.

(G) Inhibition susceptibility assay was carried out using the general sirtuin inhibitors nicotinamide and Tenovin-6. *In vivo* lipoylated *Sau*GcvH-L, *Sau*SirTM and inhibitor in indicated concentration were incubated for 10 min prior to the addition of NAD^+. Control reactions were carried out in the absence of the inhibitor (cntr).

(H) *In vivo* lipoylated *Sau*GcvH-L was MARylated using *Sau*SirTM. 1 µM of labeled *Sau*GcvH-L was incubated with increasing amounts (0.001, 0.01, 0.05, 0.25, 1, 25 and 10 µM) of either *Sau*Macro (upper panel) or human MacroD1 (lower panel). Control reactions were carried out in the absence of a macrodomain protein (cntr).

(I) The GcvH-L lipoylation-dependence of the operonal macrodomain was tested by removing the lipoylation from lipooylated and MARylated GcvH-L via Lpa prior to carrying out the de-MARylation reaction with Macro, MacroD1 or YmdB. Reactions not treated with Lpa in the absence or presence of Macro were used as controls.

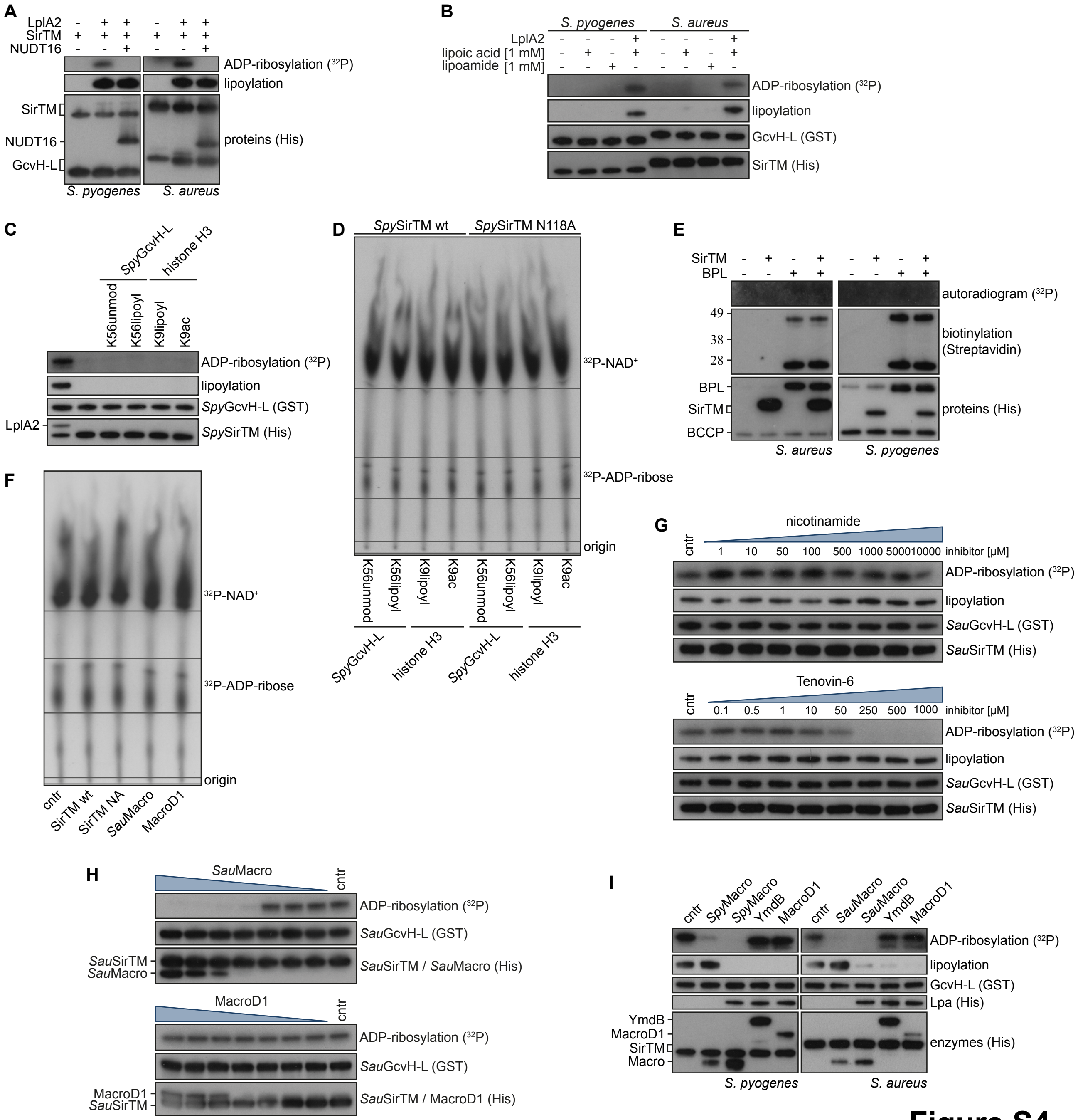
A
LplA2
SirTM
NUDT16
ADP-ribosylation (32P)
lipoylation
SirTM
NUDT16
GcvH-L
proteins (His)
S. pyogenes
S. aureus
B
S. pyogenes
S. aureus
LplA2
lipoic acid [1 mM]
lipoamide [1 mM]
ADP-ribosylation (32P)
lipoylation
GcvH-L (GST)
SirTM (His)
C
SpyGcvH-L
histone H3
K56unmod
K56lipoyl
K9lipoyl
K9ac
ADP-ribosylation (32P)
lipoylation
SpyGcvH-L (GST)
LplA2
SpySirTM (His)
D
SpySirTM wt
SpySirTM N118A
32P-NAD+
32P-ADP-ribose
origin
K56unmod
K56lipoyl
K9lipoyl
K9ac
SpyGcvH-L
histone H3
E
SirTM
BPL
autoradiogram (32P)
49
38
28
biotinylation (Streptavidin)
BPL
SirTM
BCCP
proteins (His)
S. aureus
S. pyogenes
F
32P-NAD+
32P-ADP-ribose
origin
cntr
SirTM wt
SirTM NA
SauMacro
MacroD1
G
nicotinamide
cntr
1
10
50
100
500
1000
5000
10000
inhibitor [µM]
ADP-ribosylation (32P)
lipoylation
SauGcvH-L (GST)
SauSirTM (His)
Tenovin-6
cntr
0.1
0.5
1
10
50
250
500
1000
inhibitor [µM]
ADP-ribosylation (32P)
lipoylation
SauGcvH-L (GST)
SauSirTM (His)
H
SauMacro
cntr
ADP-ribosylation (32P)
SauGcvH-L (GST)
SauSirTM
SauMacro
SauSirTM / SauMacro (His)
MacroD1
cntr
ADP-ribosylation (32P)
SauGcvH-L (GST)
MacroD1
SauSirTM
SauSirTM / MacroD1 (His)
I
cntr
SpyMacro
SpyMacro
YmdB
MacroD1
cntr
SauMacro
SauMacro
YmdB
MacroD1
ADP-ribosylation (32P)
lipoylation
GcvH-L (GST)
Lpa (His)
YmdB
MacroD1
SirTM
Macro
enzymes (His)
S. pyogenes
S. aureus

Figure S4

Figure S5. Structural comparison of *Spy*SirTM and *Spy*GcvH-L with close structural homologues (related to Figure 5).

(A) Ribbon representation of a structural overlay of *Spy*SirTM (orange) with yeast HST2 (green, PDB: 1SZC) and *T. maritima Tm*Sir2 (blue, PDB: 2H4F). The structures of *Spy*SirTM and *Tm*Sir2 contain NAD^+ (yellow and dark blue, respectively) and HST2 carba-NAD^+ (dark green).

(B) Comparison of the NAD^+ coordination of *Spy*SirTM and *Tm*Sir2. Side chain of residues involved in the coordination or catalysis are indicated as sticks. The coloration is as in (A).

(C) De-MARylation assays performed with the operon encoded macrodomains using human MacroD1 as control and MARylated human PARP1 E988Q and PARP3 as substrates.

(D) Ribbon representation of a structural overlay of *Spy*GcvH-L (black) with canonical GcvHs from bovine (*Bta*GcvH, orange, PDB: 3KLR), pea (*Psa*GcvH, green, PDB: 1DMX) and *M. tuberculosis* (*Mtu*GcvH, yellow, PDB: 3HGB). The side chains of the lipoyl attachment site (K56) and residues involved in ADP-ribosylation (E24 and D27) are indicated in the left panel, whereas the right panel shows the displacement of the β4/β5 loop (arrow heads) due to the presence of Arg72 in GcvH-L.

(E) Comparison of residues involved in tethering the C-terminal α-helix, which is absent in GcvH-L, to the core structure. The coloration is as in (D) and helix α1, which contains the residues involved in ADP-ribose attachment (GcvH-L, highlighted), is shown in dark blue. Electrostatic interaction are given as dotted lines. Note, the α-helix/core structure interaction minimally consists of a structural motif composed of four conserved residues ([YF], H, [ST] and Y) that is extended by non-conserved interactions in the cases of the bovine and pea GcvHs. Residue numbers correspond to endogenous full-length proteins as shown in the alignment in Figure S6B.

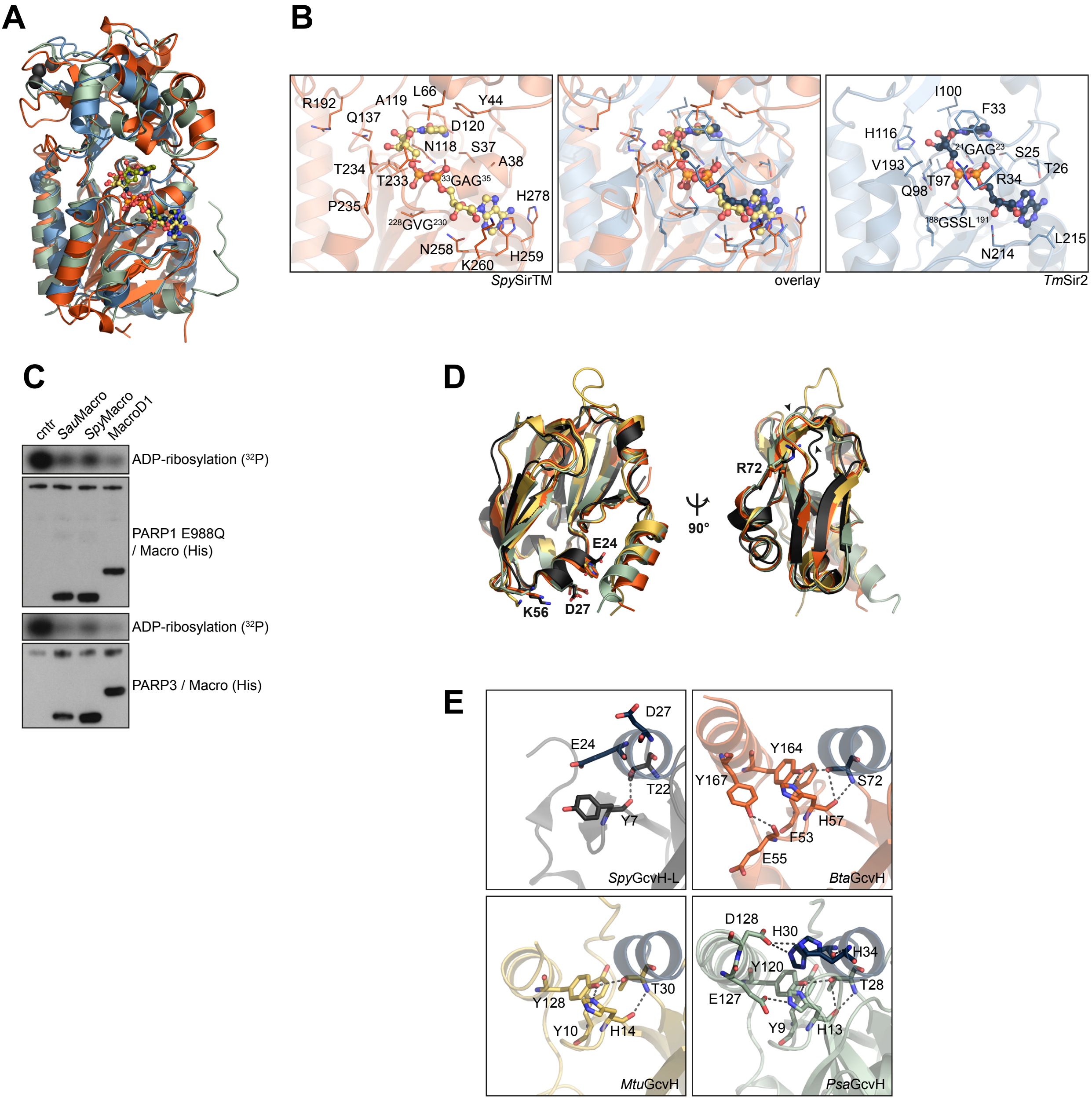
A
B
R192
A119
L66
Y44
Q137
D120
N118
S37
A38
T234
T233
^{33}GAG^{35}
H278
P235
^{228}GVG^{230}
N258
H259
K260
*Spy*SirTM
overlay
I100
F33
H116
^{21}GAG^{23}
S25
V193
T26
T97
R34
Q98
^{188}GSSL^{191}
L215
N214
*Tm*Sir2
C
cntr
SauMacro
SpyMacro
MacroD1
ADP-ribosylation (^{32}P)
PARP1 E988Q / Macro (His)
ADP-ribosylation (^{32}P)
PARP3 / Macro (His)
D
R72
E24
90°
K56
D27
E
D27
E24
T22
Y7
*Spy*GcvH-L
Y164
Y167
S72
H57
F53
E55
*Bta*GcvH
T30
Y128
Y10
H14
*Mtu*GcvH
D128
H30
H34
Y120
T28
E127
Y9
H13
*Psa*GcvH

Figure S5

Figure S6. Comparison of GcvH-Ls amongst each other and in comparison to canonical GcvHs (related to Figure 5).

(A) Multiple sequence alignment of representative GcvH-L proteins. The secondary structure elements of *Spy*GcvH-L are indicated on top of the alignment and the lipoyl attachment site underneath (yellow diamond).

(B) Multiple sequence alignment comparing *Sau*GcvH-L and *Spy*GcvH-L with canonical GcvH proteins (including bacterial, archaeal, fungal, plant and animal representatives). Secondary structure elements of *Spy*GcvH-L (orange) and *Mtu*GcvH (blue) are indicated on top of the alignment and the lipoyl attachment side underneath (yellow diamond). Note, sequences from higher organism contain an N-terminal mitochondrial targeting sequence (*Psa*-, *Cga*-, *Foc1*- and *Bta*GcvH). For sequence accession numbers see Table S2.

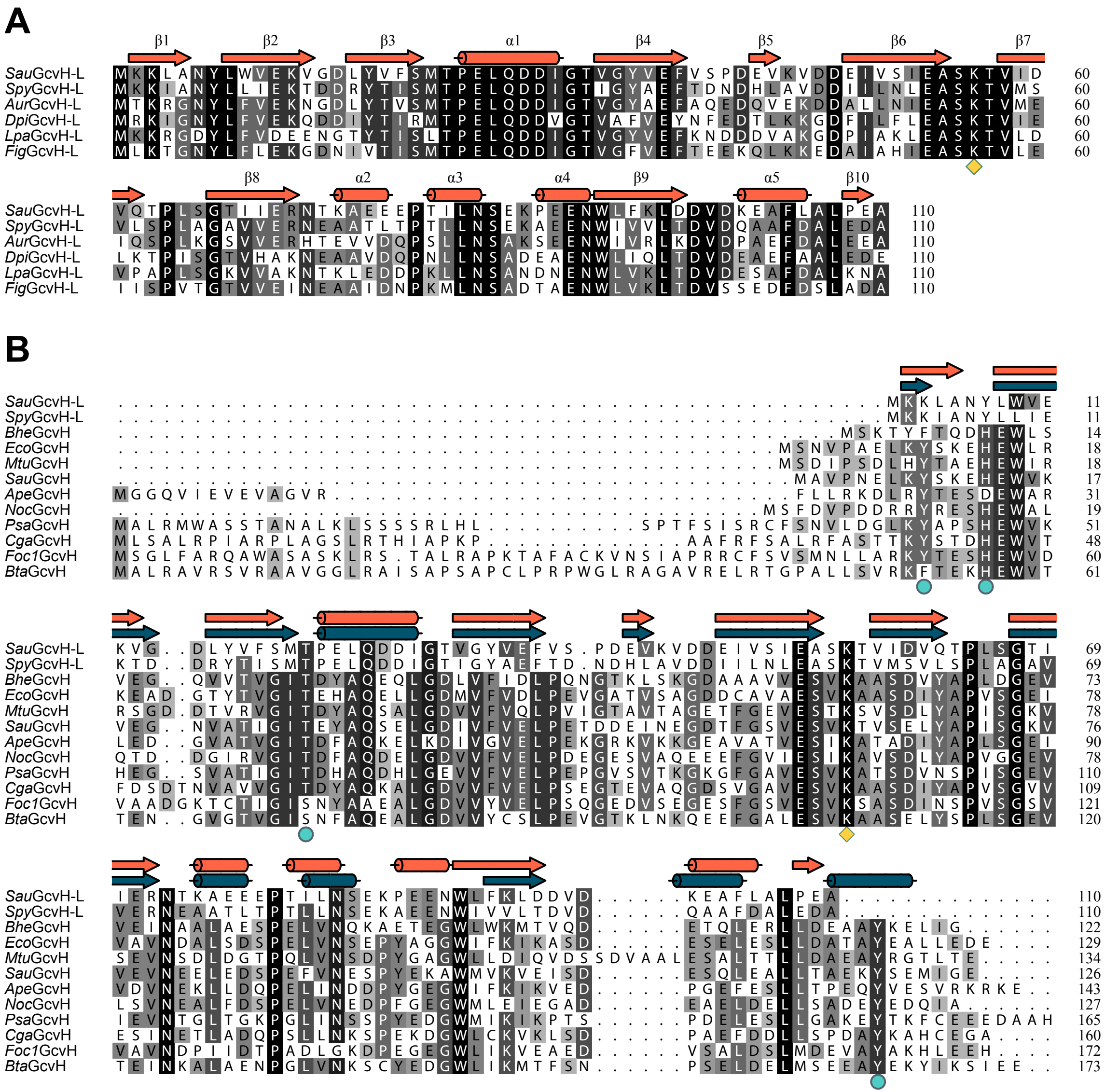

Figure S6

Figure S7. *C. albicans mfs1Δ* mutant construction and confirmation (related to Figure 6).

(A) Schematic representation of the genomic locus of wild type *C. albicans* or *mfs1Δ* (*orf19.2285*) mutant clones. The position of the Southern blot probe and restriction sites used for clone verification are shown.

(B) Genomic DNA from wild type *C. albicans* or *mfs1Δ* mutant clones was isolated and digested with ClaI and NcoI (see (A) for positions). The Southern blot assay was performed using the NEBlot Phototope Kit and the Phototope-Star Detection Kit, as per manufacturer's instructions.

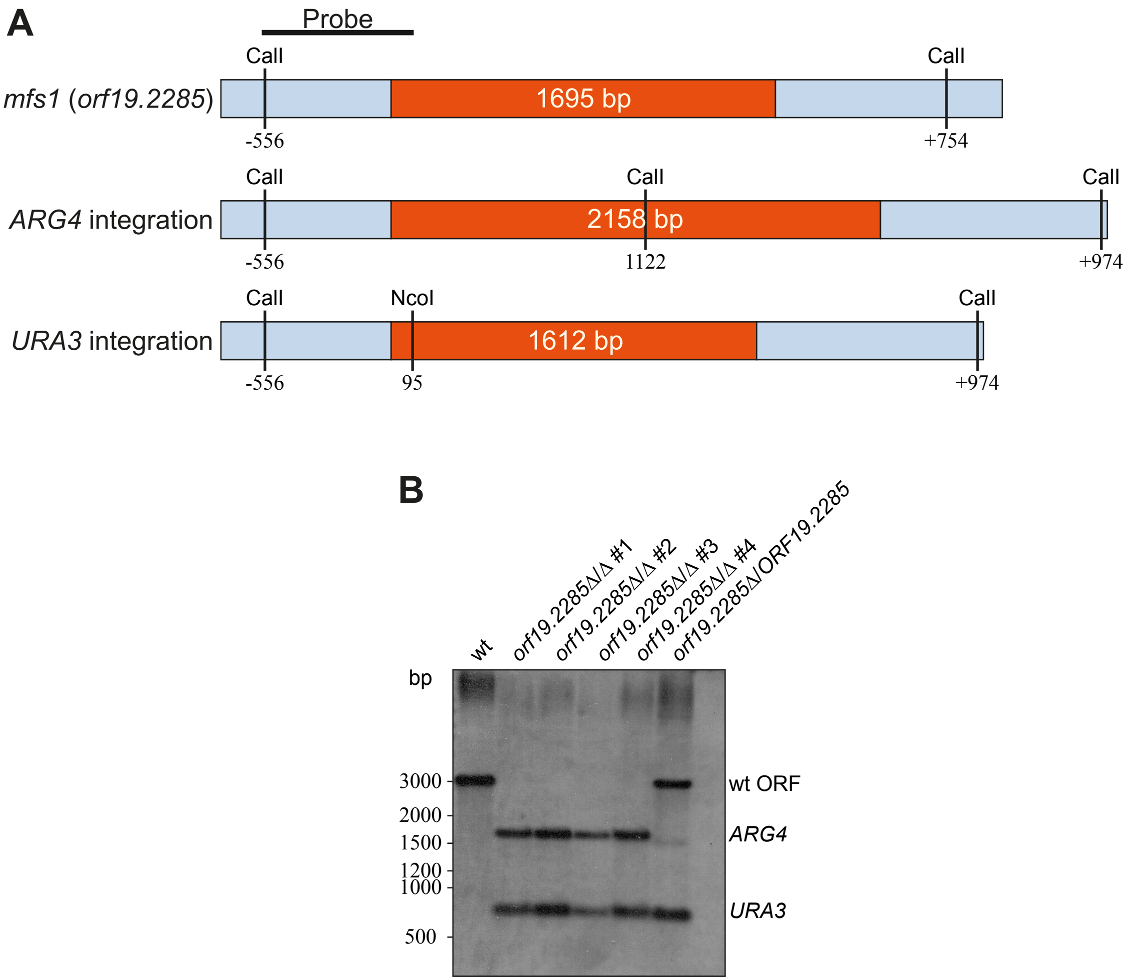

A
Probe
Call
Call
mfs1 (orf19.2285)
1695 bp
-556
+754
Call
Call
Call
ARG4 integration
2158 bp
-556
1122
+974
Call
NcoI
Call
URA3 integration
1612 bp
-556
95
+974
B
wt
orf19.2285Δ/Δ #1
orf19.2285Δ/Δ #2
orf19.2285Δ/Δ #3
orf19.2285Δ/Δ #4
orf19.2285Δ/ORF19.2285
bp
3000
2000
1500
1200
1000
500
wt ORF
ARG4
URA3

Figure S7

Supplemental Tables

Table S1. Proposed interactions for class M defining residues of *Spy*SirTM (related to Figure 1 and 5).

**Motif**	**Residues in *Spy*SirTM**	**Role**
Cx(3)C[a]	Cys145 – Cys149	Part of Zn^{2+} coordinating motif present in the small variant domain.
GVGx[NT]TP	Gl229 – Pro235	The GVGx[NT]TP motif is located on the loop connecting the Rossmann fold β6 sheet with α14 helix and forms H-bonds between the backbone nitrogen and α- and β-phosphate groups of the NAD^+ substrate.
GAGxSAx(2)Gx(2)Y	Gly33 – Tyr44	Part of the GAGxSAx(2)Gx(2)Y motif are located on the loop connecting β1 sheet with α2 helix. The side chains form both hydrogen bonds with the pyrophosphates and VdW interactions with the nicotinamide moiety of NAD^+.
T[ST]N[VA]D	Thr116-Asp120	Present on the loop between the β2 strand and the α8 helix. Forms VdW interactions with the nicotinamide ribose moiety and water mediated H-bond with the β-phosphate group.
QG	Gln137 + Gly138	The glutamine is involved in the catalytic mechanism.
R	Arg192	Presumed catalytic. Forms hydrogen bonds with the Gln137 residue either polarizing it for catalysis or involved in target recognition.
NH	Asn258 + His259	Present on the loop connecting the Rossmann fold β7 and β8. Asn258 forms H-bonds with the 3'-OH and 2'-OH groups of the adenine ribose and the His259 imidazole ring forms stacking interactions against the adenine moiety upon binding of NAD^+.

(a) The spacing between the first two cysteine residues coordinating the zinc ion is specific for class M sirtuins. All other classes contain a Cx(2)C motif.

Table S2. Accession numbers of sequences used in the generation of multiple sequence alignments (related to Figure S1 and S6).

**Abbreviation**	**Organism**	**accession**[a]
Comparison of SirTMs		
*Cal*Mfs1	*Candida albicans*	238883620
*Ate*Mfs1	*Aspergillus terreus*	115388759
*Foc1*Mfs1	*Fusarium oxysporum f. sp. cubense* RACE 1	477523926
*Aur*SirTM	*Aerococcus urinae*	326651405
*Spy*SirTM	*Streptococcus pyogenes*	14246094
*Sau*SirTM	*Staphylococcus aureus*	134271959
*Cbo*SirTM	*Clostridium bolteae*	524018386
*Ssp*SirTM	*Selenomonas sputigena*	330839837
Comparison of sirtuin classes		
*Sau*SirTM	*Staphylococcus aureus*	134271959
*Spy*SirTM	*Streptococcus pyogenes*	14246094
*Sau*CobB	*Staphylococcus aureus*	88196109
*Tb*Sir2	*Trypanosoma brucei*	74830204
*Tm*Sir2	*Thermotoga maritima*	38257895
HST2	*Saccharomyces cerevisiae*	1708326
*Atr*Sir2L1	*Amborella trichopoda*	586696836
SIRT1	*Homo sapiens*	7657575
SIRT4	*Homo sapiens*	38258657
SIRT5	*Homo sapiens*	38258652
SIRT7	*Homo sapiens*	38258650
Comparison of GcvH-Ls		
*Sau*GcvH-L	*Staphylococcus aureus*	14246092
*Spy*GcvH-L	*Streptococcus aureus*	134271957
*Aur*GcvH-L	*Aerococcus urinae*	326650593
*Dpi*GcvH-L	*Dolosigranulum pigrum*	374563578
*Lpa*GcvH-l	*Lactobacillus parafarraginis*	363716801
*Fig*GcvH-L	*Facklamia ignava*	405579715
Comparison of GcvH-Ls and canonical GcvHs		
*Sau*GcvH-L	*Staphylococcus aureus*	14246092
*Spy*GcvH-L	*Streptococcus pyogenes*	134271957
*Bhe*GcvH	*Bartonella henselae*	654326147
*Eco*GcvH	*Escherichia coli*	253978883
*Mtu*GcvH	*Mycobacterium tuberculosis*	395138596
*Sau*GcvH	*Staphylococcus aureus*	14246602
*Ape*GcvH	*Aeropyrum pernix*	499164536
*Noc*GcvH	*Natronococcus occultus*	505134954
*Psa*GcvH	*Pisum sativum*	121080
*Cga*GcvH	*Cryptococcus gattii*	757372505
*Foc1*GcvH	*Fusarium oxysporum f. sp. cubense* RACE 1	477512885
*Bta*GcvH	*Bos taurus*	27807309

(a) GenBank accession numbers

Supplemental Experimental Procedures

Sample analysis and antibodies

Reactions for analysis were stopped by adding LDS sample buffer and incubation for 5 min at 90°C. Subsequently the samples were resolved by SDS-PAGE and either vacuum dried for autoradiography or transferred onto a PVDF membrane. Immunoblot analyses were carried out using primary and secondary antibodies as indicated. Proteins were detected by enhanced chemiluminescence (Pierce).

Antibodies used in this study: Mouse monoclonal hexahistindine antibody (Clonetech), rabbit polyclonal lipoic acid antibody (Calbiochem), rabbit polyclonal histone H3 antibody (Millipore), rabbit acetylated lysine antibody (Cell Signaling Technology) and rabbit polyclonal succinyllysine antibody (PTM Biolabs), horseradish peroxidase-conjugated goat polyclonal GST antibody (Abcam), streptavidin (Pierce), secondary swine anti-rabbit (Dako) and secondary goat anti-mouse antibodies (Dako).

Plasmid construction

The construction of the human MacroD1, PARG, PARP1 E988Q and SIRT2, *E. coli* YmdB, *E. faecalis* Lpa and *T. brucei Tb*Sir2 expression vectors were described previously (Barkauskaite et al., 2013, Chen et al., 2011, Fahie et al., 2009, Rack et al., 2014, Slade et al., 2011, Spalding and Prigge, 2009). The coding sequences of all constructs from *S. aureus* and *S. pyogenes* were amplified from genomic DNA (*S. aureus*, ATCC 700699D-5; *S. pyogenes*, kind gift of Christoph Tang). The pET9H$_3$ expression vector was constructed by adaptor based cloning of 5' – GGATCCACGC GTTAACCGGT ACCAACCAAC CATGGGTCCC TGTGGTAACA CTTCCAGGCC ACTACCGTGA TGATGGTGGT GATGGTGCAT into the parental pET9d (Novagen) vector via the NcoI/BamHI restriction sides and thereby introducing a hepta-histidine tag, a HRV3C cleavage site and a small multiple cloning site into the vector. The underlined sequences mark the restored BamHI and altered NcoI side respectively. For GST-affinity purification the coding sequences of *Eco*GcvH, *Sau*GcvH and -L, *Spy*GcvH-L, *Sau*SirTM and *Sau*LplA2 were transferred into pDEST15 and the sequences of *Sau*OYE, *Sau*LLM and *Sau*Macro into pDEST24 via gateway cloning (Life Technology) using pDONR221 as donor vector. GST was expressed from the empty pGEX-4T1

vector (GE Healthcare). For nickel-affinity purification the coding sequences of *Eco*LplA, *Sau*LplA1 and 2, *Spy*LplA2 and *Spy*OYE were transferred into pDEST17, and the sequence of *Sau*CobB was transferred into pDEST42 via gateway cloning. The coding sequences of human SIRT4ΔMTS (removing residues 1 – 28 which encode the MTS (Haigis et al., 2006) and adding a C-terminal GSG linker and hepta-histidine tag) was introduced into pET9d, the sequences of *Sau*BCCP and *Spy*BCCP were introduced into pET9H_3 (adding a N-terminal double-tryptophan for UV quantitation), the sequences *Sau*GcvH-L, *Spy*GcvH-L and *Spy*LLM were introduced into pET21a and the sequences of *Sau*Macro, SpyMacro, *Sau*SirTM and *Spy*SirTM were introduced into pET28 via restriction based cloning. The coding sequences of *Sau*BPL and *Spy*BPL were introduced into pNH-TrxT (Savitsky et al., 2010) via ligation independent cloning.

All indicated mutations were introduced via PCR based site-directed mutagenesis.

***In silico* analysis of the sirtuin domain of SirTMs and GcvH-L**

Selection of representative sequences of formerly described sirtuin classes is based on the work of Greiss and Gartner (Greiss and Gartner, 2009). SirTM single domain sirtuins from bacterial operon are *Clostridium bolteae* (*Cbo*SirTM, gi|524018386), *Selenomonas sputigena* (*Ssp*SirTM, gi|330839837) and *Veillonella parvula* (*Vpa*SirTM, gi|269094432) and from the extended operon *Lactobacillus parafarraginis* (*Lpa*SirTM, gi|363716803), *Staphylococcus aureus* (*Sau*SirTM, gi|57634611) and *Streptococcus pyogenes* (*Spy*SirTM, gi|13622344). Sirtuin domain sequences from fungal fusion proteins *Candida albicans* (*Cal*Mfs1, gi|238883620), *Entamoeba dispar* (*Edi*Mfs1, gi|167540052) and *Fusarium oxysporum f. sp. cubense* RACE1 (*Foc1*Mfs1, gi|477523926) were extracted based on Clustal Omega alignment (Sievers et al., 2011) with bacterial single domain sirtuins. Sequences for tree construction were aligned using JalView 2.8.0b1 (Waterhouse et al., 2009) and the Clustal Omega program implemented therein. An unrooted phylogram was generated by neighbor-joining method using SplitTrees4 V4.13.1 (Huson and Bryant, 2006). Confidence level of the class clades was estimated to be ≥ 98.8% by bootstrapping using 10000 replicates.

Multiple sequence alignments of GcvH(-L) proteins and sirtuins were generated using JalView 2.8.0b1 and the MAFFT L-INS-I algorithm implemented therein (Katoh et al., 2005). Comparative

sirtuin domain alignment was generate by initially aligning full-length protein using MAFFT L-INS-I and extracting SirTM homology regions. The extracted sequences were re-aligned using Clustal Omega. Alignment representations were created with ALINE (Bond and Schuttelkopf, 2009). Sequences details and accession numbers are given in Table S2.

Bacteria strains and culture

For generation of *E. coli* cell extracts BL21(DE3)Δ*CobB* cells (Castano-Cerezo et al., 2015) were grown on LB agar plates supplemented with kanamycin (30 µg/ml) at 37°C. A single colony was pick and transferred into LB medium containing kanamycin. Cells were grown at 37°C and 200 rpm to an OD_{600} 0.6 and harvested by centrifugation at 4°C. The pallet was washed twice with extraction buffer [50 mM TrisHCl (pH 8), 200 mM NaCl, 1 mM DTT and 10% (v/v) glycerol] and finally resuspended in 1 mL extraction buffer. Cells were lysed by 6 cycles of sonication and insoluble cell debris removed by centrifugation at 20000 xg for 30 min at 4°C.

For generation of *S. aureus* cell extracts SH1000 cells were grown on BHI agar plates. BHI medium was inoculated with a single colony and grown overnight at 37°C and 150 rpm. The overnight culture was diluted 1:100 in fresh BHI medium and culture was allowed to grow for 2 hr. Cells were harvested by centrifugation and washed twice in extraction buffer before resuspention in 1 mL extraction buffer. Partial lysis was achieved by incubation with BugBuster protein extraction reagent (Novagen) and lysozyme for 2 hr at RT on a rotating wheel. Unlysed cell and cellular debris was removed by centrifugation at 20000 xg for 30min at 4°C.

***Candida* strains and culture**

The opening reading frame encoding amino acids 1-492 of the *Candida albicans* macrodomain-fused SirTM (*mfs1*), *orf19.2285* (C2_07280W), was deleted in the BWP17 strain background (*ura3*::imm434/*ura3*::imm434 *iro1/iro1*::imm434 *his1*::hisG/*his1*::hisG *arg4/arg4*). Selection markers *ARG4* and *URA3* were used to create a homozygous deletion mutant by homologous recombination. The marker-matched wild type strain (DAY286) was used as the control in all experiments. Confirmation of homozygous deletion by Southern blot analysis is shown in Figure S7. Strains were

maintained in 15% glycerol (v/v) at -80°C. Working stocks were streaked onto YPDUri agar plates (1% yeast extract, 2% peptone, 2% glucose, 2% agar, 80 µg/ml uridine), strains grown at 30°C for 2-3 days, and then plates maintained at room temperature. For experiments testing the sensitivity to hydrogen peroxide (H_2O_2), strains were inoculated into YPDUri media over night at 30°C, 200 rpm and grown to saturation. Cultures were then diluted to OD_{600} 0.5 and 2.5 µl dropped onto YPDUri control or H_2O_2-containing plates, followed by incubation at 30°C for 3 days. Alternatively, broth H_2O_2 susceptibility assays were performed in a 96-well microplate, following a method described by (Park et al., 2008), with modification. Overnight *C. albicans* cultures were harvested by centrifugation (5 min, 2500 xg), and re-suspended in YPD medium to a density of $1 \cdot 10^3$ cells/ml. One hundred microliters of fungal suspension were placed into a well of a 96-well microplate and mixed with H_2O_2 to reach concentrations of 0 mM, 5 mM, 5.5 mM, 6 mM, and 6.5 mM respectively. The microplates were incubated statically at 37°C for 24 hr, and fungal growth read with the Infinite 200 PRO Tecan spectrometer at OD_{600}. Percentage of fungal growth was calculated relative to no H_2O_2 controls.

Human 293T cell culture

293T cells were cultivated in high glucose DMEM (Dulbecco's modified Eagle's medium) supplemented with 10% FBS, 2 mM and L-glutamine. Cells were incubated at 37°C in humidified atmosphere with 5% CO_2. For extract preparation cells were grown on a 100 mm culture dish to confluence and washed twice with PBS. Cells were scraped off in 1 mL extraction buffer and lysed by 3 cycles of sonication. Insoluble cell debris was removed by centrifugation at 20000 xg for 30 min at 4°C.

Protein expression and purification

Recombinant proteins were expressed in Rosetta (DE3) cells grown in LB medium supplemented with 2 mM $MgSO_4$ and appropriate antibiotics at 37°C to OD_{600} 0.6. In addition, expression cultures of lipoamidase were supplemented with 2% glucose (w/v), 5 mM potassium acetate and 5 mM sodium succinate. Expression was induced with 0.4 mM IPTG and 5 µM zinc acetate in case of zinc-containing enzymes. Cells were grown at 30°C and harvested 4 hr post-induction by centrifugation.

Lipoamidase expression was carried out at 18°C overnight. SeMET *Spy*SirTM protein was produced as above but using minimal medium and SeMET growth medium (SelenoMet Medium Base plus SelenoMet Nutrient Mix, Molecular Dimensions) supplemented with 50 mg/mL of SeMET after 40 min of inoculation. The SeMET SpySirTM protein was expressed at 30 °C for 18 hr.

Recombinant His-tagged proteins were purified by Ni^{2+}-NTA chromatography (Qiagen) according to the manufacturer's protocol using the following buffers: all buffer contained 50 mM TrisHCl (pH 8) and 500 mM NaCl; additionally, the lysis/binding buffer contained 10 mM imidazole, the washing buffer contained 30 mM imidazole, and the elution buffer contained 500 mM imidazole.

Recombinant GST-tagged proteins were purified using Glutathione Sepharose 4B (GE Life Sciences) according to the manufacturer's protocol using the following buffers: lysis/binding/washing PBS containing 1 mM DTT and elution 50 mM TrisHCl (pH 8), 200 mM NaCl containing 10 mM reduced glutathione and 1 mM DTT.

All proteins were dialyzed over night against 50 mM TrisHCl (pH 8), 200 mM NaCl, 1 mM DTT.

Proteins intended for crystallization underwent affinity purification over a HisTrap HP column (GE Healthcare) followed by size exclusion chromatography using a HiLoad Superdex 75 column (GE Healthcare). Proteins were concentrated using Vivaspin 20 columns (GE Healthcare).

Lipoylation assays

Lipoylation reactions were carried out in lipoylation buffer [50 mM TrisHCl (pH 8), 200 mM NaCl, 5 mM ATP, 2.4 mM lipoic acid, 1 mM $MgCl_2$, 1 mM DTT] using 1 μM LplA and 2 μM target protein or in a 1:2 molar ratio if higher concentrations were required for subsequent reactions. Reactions were incubated for 30 min at 30°C before analysis by immunoblot or further processing.

Substrate specificity of *Sau*LplA2 was assessed by incubation of 1 µM of *Sau*GcvH-L with increasing concentrations of lipoic acid or lipoamide (0.24, 2.4, 24 or 240 µM) in the presence of 1 µM enzyme at 37°C for 30 min. Control reactions were carried out at highest substrate concentration in the absence of *Sau*LplA2. All samples were analyzed by immunoblot.

For *in vivo* lipoylation of GcvH-L, Rosetta (DE3) cells were grown as described above. Upon induction cultures were supplemented with 100 μM lipoic acid and grown for 3 hr at 30°C. Further

protein synthesis was inhibited by addition of 150 µg/ml kanamycin and cells were incubated for an additional 60 min. Control reactions were supplemented with solvent instead of lipoic acid. Samples were taken before induction and kanamycin addition and at the end of the incubation period to evaluate the success of *in vivo* lipoylation. All samples were analyzed by immunoblot.

Biotinylation assay

BCCP biotinylation was carried out in biotinylation buffer [50 mM TrisHCl (pH 8), 200 mM NaCl, 1 mM DTT, 1 mM MgCl2, 300 µM biotin and 5 mM ATP] at 30°C for 1 hr using 2 µM BCCP and 1 µM BPL. Reactions were stopped by twice purifying the protein over PD SpinTrap G25 (Ge Healthcare) columns. Purified proteins were used as substrates for debiotinylation or ADP-ribosylation reaction (see below).

Deacylation assays

Delipoylation of *in vitro* and *in vivo* lipoylated GcvH-L was carried out in deacylation buffer [50 mM TrisHCl (pH 8), 200 mM NaCl, 10 mM $MgCl_2$, 1 mM DTT, 1 mM NAD^+] using 1 µM recombinant sirtuin and 1 µM GcvH-L. Reactions were incubated at 30°C for 2 hr.

Debiotinylation was carried out analogously using purified, biotinylated BCCP as substrate (see above). All reactions were stopped by addition of LDS sample buffer.

Deacylation of bacterial and human cell extracts (40 µg) was carried out in deacylation buffer for 2 hr at 30°C. Reactions were supplemented with 2 µM recombinant sirtuin as indicated. Lpa control reaction were carried out in PBS using 1 µg crude Lpa purification. All reactions were stopped by addition of LDS sample buffer.

Delipoylation using Lpa was carried out by mixing the initial lipoylation reaction or *in vivo* lipoylated GcvH-L in lipoylation buffer (1/3 final vol.) with 10x PBS (1/10 final vol.) and 1µg crude Lpa purification. The volume was adjusted with dH_2O and the reaction incubated for 2 hr at 30°C. All reactions were stopped by addition of LDS sample buffer.

Deacetylation of synthetic penta-acetylated histone H3 (Active Motif) was carried out as described earlier (Rack et al., 2014). Briefly, deacetylation was carried out in deacylation buffer [50

mMTrisHCl (pH 8), 137 mM NaCl, 2.7 mM KCl and 10 mM $MgCl_2$, 0.5 mM DTT] using 2 µM purified sirtuin, 1 mM NAD^+ and 1 µM penta-acetylated histone H3. Reactions were incubated at 30°C for 2 hr and stopped by addition of LDS sample buffer.

Desuccinylation of a fluorogenic succinyl-peptide was tested with the Fluorogenic SIRT5 assay kit (BPS bioscience) according to the manufacturer's recommendations using 300 ng sirtuin per reaction.

For desuccinylation Xenopus-derived histone octamers were non-enzymatically succinylated as described earlier (Wagner and Payne, 2013). Briefly, succinylation reaction were carried out in succinylation buffer [50 mM TrisHCl (pH 8 at 37°C), 150 mM NaCl, 0.5 mM succinyl-CoA] using 3 µM histone octmaers as substrate. Reactions were incubated for 6 hr at 37°C at 400 rpm in an Eppendorf Thermomixer and subsequently stored on ice until further use. Desuccinylation was carried out as described for histone deacetylation using 1 µM succinylated histone octamers as substrate.

ADP-ribosylation assays

ADP-ribosylation reactions were carried out in MARylation buffer [50 mM TrisHCl (pH 8), 200 mM NaCl, 1 mM $MgCl_2$, 1 mM DTT] using 1 µM sirtuin, 1 µM target protein, 2 µCi $^{32}P\text{-}NAD^+$ and 5 µM unlabeled NAD^+. A 1:1 molar ratio was used if higher concentrations were required for subsequent reactions. Reactions were incubated for 60 min at 30°C before analysis by autoradiography and immunoblot or further processing. For kinetic studies the incubation time was varied as indicated.

ADP-ribosylation reactions using purified, biotinylated BCCP (see above) as substrate were carried out analogously.

For inhibitor studies 6 mM Tenovin-6 (Axon Medchem) stock solution was prepared in 18% DMSO (v/v) and 50 mM HCl. Further dilutions were prepared with 18% DMSO. Final DMSO concentration in the assays was 3%. Nicotinamide (Sigma) solutions were prepared in dH_2O. All reaction components (including inhibitors) were mix and incubated for 10 min at RT before starting the reaction by addition of 2 µCi $^{32}P\text{-}NAD^+$ and 10 µM unlabeled NAD^+.

For reaction containing free lipoic acid or lipoamide both compounds were dissolved in DMSO and diluted to 2% DMSO (v/v) in the final assay conditions. For reaction containing peptides (custom

synthesized by Alta Biosciences (now Abington Health); sequences are ±5 residues of the indicated modification site and two C-terminal tryptophan residues were added for UV quantitation, the N-termini are acetylated and the C-termini amide modified) peptides were dissolved in DMSO and diluted to 5 µM and 2% DMSO (v/v) in final assay conditions.

For ADP-ribosylation comparison reaction were carried out in MARylation buffer using 5 µg target protein (lipoylated *Spy*GcvH-L, glutamate dehydrogenase (Sigma) and histone H1 (New England Biolabs) respectively), 1.5 µM sirtuin, 20 µM unlabeled NAD^+ and 4 µCi ^{32}P-NAD^+. Reaction were carried out at 37°C for 2 hr. Reactions were stopped with LDS sample buffer and analyzed by autoradiography and immunoblot.

De-ADP-ribosylation assays

De-ADP-ribosylation was carried out in [50 mM TrisHCL (pH 8), 200 mM NaCl, 1 mM $MgCl_2$, 1mM DTT] out using 1 µM radiolabelled GcvH-L and 1 µM macrodomain protein. The reactions were incubated for 1 hr at 30°C.

For the concentration gradient experiment using *Sau*Macro and human MacroD1 reactions containing the desired final concentration of macrodomain protein were prepared and started by addition of the initial ADP-ribosylation reaction to achieve a final concentration of 1 µM *Sau*GcvH-L in the reaction.

For the de-MARylation assay using PAR1 EQ and PARP3 self-modification was carried out in PARP buffer [50 mM TrisHCl, 50 mM NaCl, 4 mM $MgCl_2$ and 0.2 mM DTT] using 1 µM enzyme for 30 min at 30°C. The reaction were terminated by addition of 2 µM olaparib.

For direct visualization of radiolabeled ADP-ribose Macro reaction were carried out as follows: 20 µM *in vivo* lipoylated *Sau*GcvH-L were incubated in MARylation buffer in presence of 2 µM *Sau*SirTM wt or N123A protein and 5 µCi ^{32}P-NAD^+ for 45 min at 30°C followed by addition of 20 µM of unlabeled-NAD^+ and incubation for 15 min at 30°C. The reactions were stopped by applying the samples over a PD SpinTrap G25 (GE Healthcare) column in order to remove unbound radiolabeled NAD^+. Subsequently, the reactions were re-supplemented with the MARylation buffer and incubated with 2 µM of S*au*Macro or human MacroD1 protein at 30°C for 1 hr. As control,

poly(ADP-ribose) was synthesized by the self-modification of PARP1 in a reaction mixture containing 6.4 units of PARP1 (Trevigen), 10 μM unlabeled–NAD^+ (Trevigen), 4.4 μCi ^{32}P-NAD^+, activated DNA (Trevigen), 50 mM TrisHCl (pH 7.5) and 50 mM NaCl at RT. Reactions were stopped after 45 min by the addition of the PARP inhibitor KU-0058948 and were purified twice over PD SpinTrap G25 (GE Healthcare) columns to remove unbound radiolabel. Subsequently, the reactions were re-supplemented with 50 mM Tris (pH 7.5) and 50 mM NaCl and 1 μM PARG were added to the reaction. The samples were incubated for 45 min at RT.

All samples were heat-inactivated by incubating the reactions at 80°C for 5 min, spotted onto polyethyleneimine (PEI)-cellulose plates (Macherey-Nagel, Polygram CEL 300 PEI/UV_{254}) and developed in 0.2 M LiCl and 0.2 M formic acid. Dried plates were exposed on X-ray film.

NUDT16 phosphordiesterase assay

To monitor the loss of incorporated radiolabel, ADP-ribosylated GcvH-L (see above) was supplemented with 15 mM $MgCl_2$ and 4 μM NUDT16 and incubated for 3 hr at 30°C. Reactions were stopped with LDS sample buffer and analyzed by autoradiography and immunoblot.

NAD hydrolase activity assay

1 μM *Sau*SirTM wt, *Sau*SirTM N123A, *Sau*Macro or MacroD1 was incubated with 2 μCi ^{32}P-NAD^+, 5 μM unlabeled-NAD^+ in MARylation buffer at 30°C for 1 hr. The reactions were heat-inactivated by incubation at 80°C for 5 min and spotted onto polyethyleneimine (PEI)-cellulose plates (Macherey-Nagel, Polygram CEL 300 PEI/UV_{254}) and developed in 0.2 M LiCl and 0.2 M formic acid. Dried plates were exposed on X-ray film.

GST pull-down assays

Pull-down assays were carried out on 0.2-μm filters (Ultrafree, Millipore), using either 50 μl or 100 μl volumes in all steps. All pull-down assays were carried out at room temperature. Filters were moistened with TZNK/D/T buffer [TZNK buffer (50 mM TrisHCl (pH 8), 150 mM KCl, 12 mM NaCl, 100 μM zinc acetate, 2 mM $MgCl_2$) containing 5 mM DTT and 0.1% (v/v) Triton X-100] before adding glutathione Sepharose 4B. The beads were drained after each step by centrifugation for

30 s. GST-fusion proteins (5 μM) were immobilized on beads by incubation for 30 min on a rotating wheel. Unbound proteins were removed by centrifugation. Beads were washed twice with TZNK/D/T buffer, before introducing 5 μM of the binding partner. Binding reactions were carried out for 30 min on a rotating wheel, before unbound proteins were removed by centrifugation. Beads were washed four times in TZNK/D/T buffer and twice in 50 mM TrisHCl (pH 8), 200 mM NaCl, and 1 mM DTT. Proteins were eluted from the beads by incubation with elution buffer for 30 min on a rotating wheel. Samples were analyzed by immunoblot.

Crystallization

Native and selenomethionine (SeMET) labeled *Spy*SirTM proteins for crystallization were expressed as described above and purified protein concentrated to 14 mg/ml in 25 mM TisHCl (pH 8), 100 mM NaCl and 1 mM DTT.

Initial *Spy*SirTM crystallization conditions were identified using the Morpheus HT-96 matrix screen (Molecular dimensions). Reproducible crystallization was ensured by seeding as follows: crystals were grown at 293 K by sitting-drop vapor diffusion in MRC 96 well plates (Molecular Dimensions) by mixing 120 nL purified protein with 30 nL of seed stock and 150 nL of precipitant solution consisting of 0.1 M amino acids, 0.1 M buffer system 1 (pH 6.5), 30.00% (v/v) EDO_P8K (Molecular Dimensions). Seed stock was prepared using a Seed Bead^{TM} (Hampton Research) with one or more seed crystals and mother liquor solution to 100 µL as the stabilizing solution. Crystals appeared over 48 hr and continued to grow for a further week. For the ADPr and NAD^+ complexes, native crystals were soaked with 1 mM ligand in the mother liquor for 20 hr and 10 min, respectively. The crystals were cryoprotected by dipping them into a solution of 20% ethylene glycol in mother liquor and vitrified by submersion in liquid nitrogen.

Native *Spy*GcvH-L protein for crystallization was expressed as described above and purified protein concentrated to 56 mg/ml in 25 mM TisHCl (pH 8), 100 mM NaCl and 1 mM DTT. A single crystal grew at 293 K by sitting-drop vapor diffusion in a MRC 96 well plate by mixing 100 nL purified protein with 100 nL of precipitant solution consisting of 2.0 M NaH_2PO_4/K_2HPO_4 (pH 7.0). The crystal was cryoprotected in a solution of precipitant plus 15% PEG400.

X-ray Data Collection, Processing, Structure Determination, Refinement, and Analysis

X-ray diffraction data were collected using synchrotron radiation at Diamond Light Source (Rutherford Appleton Laboratory, Harwell, UK) (Table 2) and integrated using iMOSFLM (Battye et al., 2011). Space group identity was checked with POINTLESS (Evans, 2006) before the data sets were scaled with AIMLESS (Evans, 2006). The program autoSHARP (Murshudov et al., 1997) was used for locating the Se atoms in the apo-*Spy*SirTM selenomethionine derivate as well as for refinement and initial phase calculation. *Spy*GcvH-L data was solved via molecular replacement using PHASER (Storoni et al., 2004) with a model produced from *Thermus thermophilus* GcvH protein (pdb 1ONL) after removing the N- and C-termini, flexible loops and changing the sequence to polyalanine. Density modification was implemented using PARROT (Cowtan, 2010) and initial models were build using the automated model building program BUCCANEER (Cowtan, 2006). Model building for all structures were carried out with COOT (Emsley and Cowtan, 2004) and real space refinement with REFMAC5 (Murshudov et al., 1997).

Supplemental References

Barkauskaite, E., Brassington, A., Tan, E.S., Warwicker, J., Dunstan, M.S., Banos, B., Lafite, P., Ahel, M., Mitchison, T.J., Ahel, I., *et al.* (2013). Visualization of poly(ADP-ribose) bound to PARG reveals inherent balance between exo- and endo-glycohydrolase activities. Nature communications *4*, 2164.

Battye, T.G., Kontogiannis, L., Johnson, O., Powell, H.R., and Leslie, A.G. (2011). iMOSFLM: a new graphical interface for diffraction-image processing with MOSFLM. Acta crystallographica. Section D, Biological crystallography *67*, 271-281.

Bond, C.S., and Schuttelkopf, A.W. (2009). ALINE: a WYSIWYG protein-sequence alignment editor for publication-quality alignments. Acta crystallographica. Section D, Biological crystallography *65*, 510-512.

Castano-Cerezo, S., Bernal, V., Rohrig, T., Termeer, S., and Canovas, M. (2015). Regulation of acetate metabolism in Escherichia coli BL21 by protein N(epsilon)-lysine acetylation. Applied microbiology and biotechnology *99*, 3533-3545.

Cowtan, K. (2006). The Buccaneer software for automated model building. 1. Tracing protein chains. Acta crystallographica. Section D, Biological crystallography *62*, 1002-1011.

Cowtan, K. (2010). Recent developments in classical density modification. Acta crystallographica. Section D, Biological crystallography *66*, 470-478.

Evans, P. (2006). Scaling and assessment of data quality. Acta crystallographica. Section D, Biological crystallography *62*, 72-82.

Huson, D.H., and Bryant, D. (2006). Application of phylogenetic networks in evolutionary studies. Molecular biology and evolution *23*, 254-267.

Katoh, K., Kuma, K., Toh, H., and Miyata, T. (2005). MAFFT version 5: improvement in accuracy of multiple sequence alignment. Nucleic acids research *33*, 511-518.

Murshudov, G.N., Vagin, A.A., and Dodson, E.J. (1997). Refinement of macromolecular structures by the maximum-likelihood method. Acta crystallographica. Section D, Biological crystallography *53*, 240-255.

Park, B., Nizet, V., and Liu, G.Y. (2008). Role of Staphylococcus aureus catalase in niche competition against Streptococcus pneumoniae. Journal of bacteriology *190*, 2275-2278.

Savitsky, P., Bray, J., Cooper, C.D., Marsden, B.D., Mahajan, P., Burgess-Brown, N.A., and Gileadi, O. (2010). High-throughput production of human proteins for crystallization: the SGC experience. Journal of structural biology *172*, 3-13.

Sievers, F., Wilm, A., Dineen, D., Gibson, T.J., Karplus, K., Li, W., Lopez, R., McWilliam, H., Remmert, M., Soding, J., *et al.* (2011). Fast, scalable generation of high-quality protein multiple sequence alignments using Clustal Omega. Molecular systems biology *7*, 539.

Storoni, L.C., McCoy, A.J., and Read, R.J. (2004). Likelihood-enhanced fast rotation functions. Acta crystallographica. Section D, Biological crystallography *60*, 432-438.

Wagner, G.R., and Payne, R.M. (2013). Widespread and enzyme-independent Nepsilon-acetylation and Nepsilon-succinylation of proteins in the chemical conditions of the mitochondrial matrix. The Journal of biological chemistry *288*, 29036-29045.

Waterhouse, A.M., Procter, J.B., Martin, D.M., Clamp, M., and Barton, G.J. (2009). Jalview Version 2--a multiple sequence alignment editor and analysis workbench. Bioinformatics *25*, 1189-1191.
