# Supplementary material for: Identification of a Class of Protein ADP-Ribosylating Sirtuins in Microbial Pathogens
Source: Mol Cell. 2015 Jul 16;59(2):309–20. doi: 10.1016/j.molcel.2015.06.013 (PMC4518038; doi:10.1016/j.molcel.2015.06.013)
Supplement: Document S2. Article plus Supplemental Information [file mmc2.pdf]

Article

Molecular Cell

Identification of a Class of Protein ADP-Ribosylating Sirtuins in Microbial Pathogens

Graphical Abstract

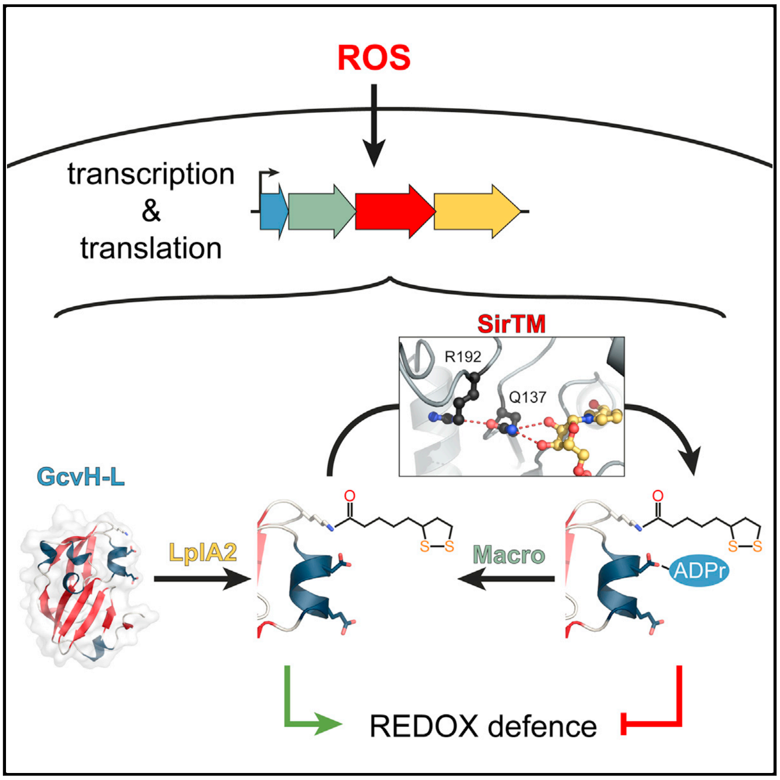

Authors

Johannes Gregor Matthias Rack, Rosa Morra, Eva Barkauskaite, ..., David Leys, Ana Traven, Ivan Ahel

Correspondence

ivan.ahel@path.ox.ac.uk

In Brief

Oxidative stress has been recognized as a critical factor in human disease, aging, and the immune system function. Rack et al. report a structural and biochemical analysis of a sirtuin/macrodomain system modulating the oxidative stress response in pathogenic microorganisms via reversible protein ADP-ribosylation.

Highlights

- A class of sirtuins (SirTMs) is identified in microbial pathogens
- SirTMs are linked to macrodomains and act as protein ADP-ribosyltransferases
- Protein ADP-ribosylation by SirTMs is strictly lipoylation dependent and reversible
- SirTMs modulate the response to oxidative stress

Accession Numbers

5A35
5A3A
5A3B
5A3C

Rack et al., 2015, Molecular Cell *59*, 309–320
July 16, 2015 ©2015 The Authors
http://dx.doi.org/10.1016/j.molcel.2015.06.013

Molecular Cell
Article

Identification of a Class of Protein ADP-Ribosylating Sirtuins in Microbial Pathogens

Johannes Gregor Matthias Rack,[1,8] Rosa Morra,[2,8] Eva Barkauskaite,[1,8] Rolf Kraehenbuehl,[7,9] Antonio Ariza,[1] Yue Qu,[3] Mary Ortmayer,[2] Orsolya Leidecker,[6] David R. Cameron,[4] Ivan Matic,[6] Anton Y. Peleg,[3,4] David Leys,[2] Ana Traven,[5] and Ivan Ahel[1,*]
[1]Sir William Dunn School of Pathology, University of Oxford, South Parks Road, Oxford OX1 3RE, UK
[2]Manchester Institute of Biotechnology, University of Manchester, 131 Princess Street, Manchester M1 7DN, UK
[3]Department of Microbiology, Monash University, Malvern East, VIC 3145, Australia
[4]Department of Infectious Diseases, The Alfred Hospital and Monash University, Malvern East, VIC 3145, Australia
[5]Department of Biochemistry and Molecular Biology, Monash University, Malvern East, VIC 3145, Australia
[6]Max Planck Institute for Biology of Ageing, Joseph-Stelzmann-Strasse 9b, Cologne 50931, Germany
[7]Cancer Research UK Manchester Institute, Wilmslow Road, Manchester M20 4BX, UK
[8]Co-first author
[9]Present address: School of Biological Sciences, Bangor University, Brambell Building, Deiniol Road, Bangor, Gwynedd LL57 2UW, UK
*Correspondence: ivan.ahel@path.ox.ac.uk
http://dx.doi.org/10.1016/j.molcel.2015.06.013
This is an open access article under the CC BY license (http://creativecommons.org/licenses/by/4.0/).

SUMMARY

Sirtuins are an ancient family of NAD$^+$-dependent deacylases connected with the regulation of fundamental cellular processes including metabolic homeostasis and genome integrity. We show the existence of a hitherto unrecognized class of sirtuins, found predominantly in microbial pathogens. In contrast to earlier described classes, these sirtuins exhibit robust protein ADP-ribosylation activity. In our model organisms, *Staphylococcus aureus* and *Streptococcus pyogenes*, the activity is dependent on prior lipoylation of the target protein and can be reversed by a sirtuin-associated macrodomain protein. Together, our data describe a sirtuin-dependent reversible protein ADP-ribosylation system and establish a crosstalk between lipoylation and mono-ADP-ribosylation. We propose that these posttranslational modifications modulate microbial virulence by regulating the response to host-derived reactive oxygen species.

INTRODUCTION

Sirtuins are a diverse enzyme family of NAD$^+$-dependent protein deacylases that control a variety of cellular processes including cell cycle progression, maintenance of genome integrity, and metabolic homeostasis (Asher and Schibler, 2011; Choi and Mostoslavsky, 2014). The overall structure of sirtuins is comprised of a highly conserved Rossmann fold and a more diverse zinc coordinating domain (Yuan and Marmorstein, 2012). The reaction mechanism is initialized by activation of NAD$^+$, followed by a nucleophilic attack and release of nicotinamide. In the case of deacylation, a reactive imidate intermediate is formed that can undergo base-exchange with nicotinamide, thereby inhibiting reaction progression (reviewed in Sauve, 2010). Phylogenetically, the sirtuin family can be divided into five classes (I–IV and U, see Figure 1A) (Frye, 2000; Greiss and Gartner, 2009), and a correlation between sirtuin class and substrate preference was recently suggested (Dölle et al., 2013; He et al., 2012). For example, human SIRT1, a class I sirtuin, is most efficient at deacetylation, whereas SIRT5, belonging to class III, has highest activity toward succinylation (Du et al., 2011; Feldman et al., 2013). Although sirtuins appear to be primarily deacylases, several studies have suggested that some also possess protein ADP-ribosyltransferase activity (Haigis et al., 2006; Kowieski et al., 2008). Posttranslational ADP-ribosylation influences various cellular processes, such as transcription, chromatin organization, nitrogen fixation, and DNA repair, via modification of different acceptor proteins (Barkauskaite et al., 2013; Feijs et al., 2013; Nordlund and Högbom, 2013).

Macrodomains are evolutionary widespread ADP-ribose-binding domains (Till and Ladurner, 2009) that have the potential to reverse sirtuin reactions either by hydrolysis of ADP-ribosylated protein substrates (Barkauskaite et al., 2013), or by deacylating *O*-acyl-ADP-ribose (Chen et al., 2011; Peterson et al., 2011).

In this study, we report on the identification of a distinct class of sirtuins (SirTMs) found primarily in pathogenic microorganisms and show that these function as protein ADP-ribosyl transferases. Members of this sirtuin class are genetically linked to a specific subclass of macrodomain proteins, which reverse the sirtuin catalyzed ADP-ribosylation. Our structural and biochemical analysis suggest that SirTMs possess class-specific features that may explain the preference for protein ADP-ribosylation. Moreover, we show that in *Staphylococcus aureus* and *Streptococcus pyogenes* the sirtuin-mediated ADP-ribosylation is dependent on another posttranslational modification—lipoylation. We propose that a crosstalk between these two types of protein modifications is important for the response of microbial pathogens to oxidative stress, a potent host defense mechanism.

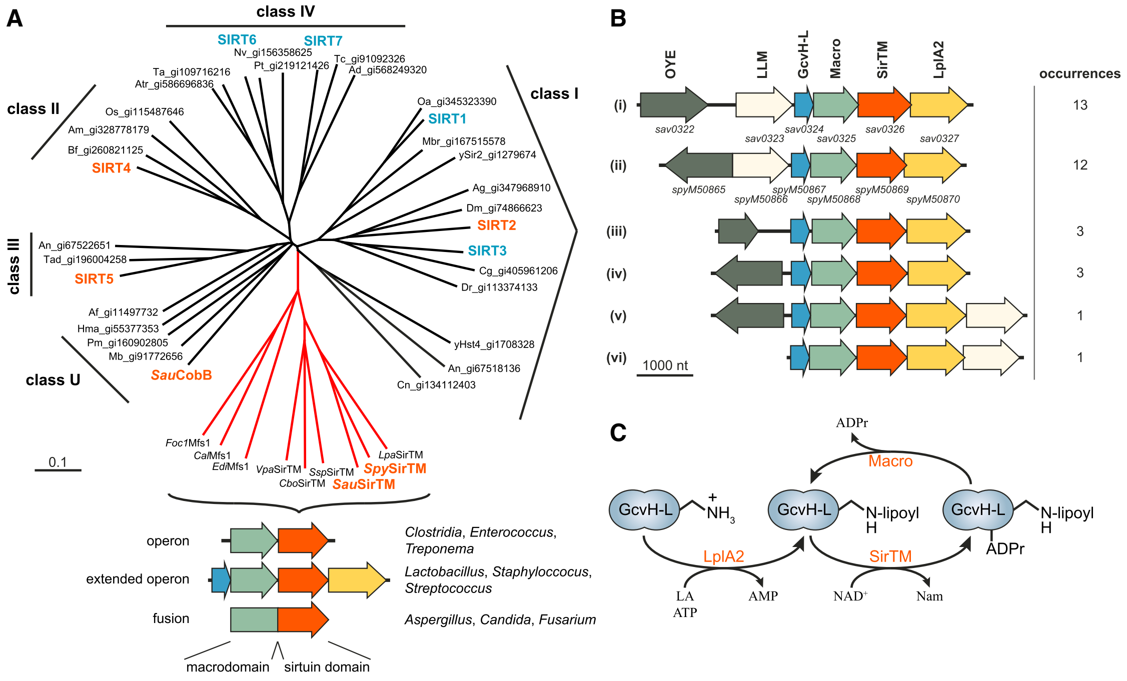

Figure 1. Macrodomain-Associated Sirtuins Form a Distinct Class within the Sirtuin Family

(A) Unrooted phylogram illustrating the relationship between the known sirtuin classes (I–IV and U) and sirtuins found associated with macrodomains. The latter form a distinct class among the sirtuins (highlighted in red). Human sirtuins (SIRT1–7) are highlighted in blue and sirtuins used in this study in orange. Schematic genome arrangements of the macrodomain-linked sirtuins are given underneath the tree. Details regarding class M-specific motifs can be found in Figure S1 and Table S1.

(B) Schematic overview and organization of the extended SirTM operons. In addition to the sirtuin/macrodomain, the operon encodes a glycine cleavage system H-like (GcvH-L) protein and lipoate protein ligase A (LplA2). The operon is commonly associated with the ORFs of homologs of the Old Yellow Enzyme (OYE) and bacterial luciferase-like monooxygenase (LLM) family (i, ii, and v). Less frequently, association with only one of the two ORFs can be observed (iii, iv, and vi). The encoded proteins are indicated on top of the schemes and the gene loci of *S. aureus* (*sav0322–sav0327*) and *S. pyogenes* (*spyM50865–spyM50870*) are indicated underneath the corresponding schemes.

(C) Scheme of the functional relationship between the extended operon components.

RESULTS

Identification of a Distinct Class of Sirtuins

By analyzing available genomic data, we have identified a phylogenetically distinct class of microbial sirtuins (Figure 1A) (Chen et al., 2011 and this study). At the primary sequence level, members of this clade are characterized by a set of short sequence motifs, which are only distantly related to the intra- and interclass sirtuin motifs described by Frye (for details see Figure S1 and Table S1) (Frye, 2000). The most surprising difference is the replacement of the absolutely conserved HG motif containing the catalytic H residue by QG (Figure S1C). These microbial sirtuins are further characterized by their genetic linkage to a subclass of macrodomain proteins. Hence, we designate this class of macrodomain-linked sirtuins as "class M" and their members as "SirTMs." In bacteria the genes for SirTMs and the associated macrodomains lie adjacent within the same operon. In fungi the two open reading frames (ORFs) are fused to encode a single macrodomain-SirTM polypeptide that we termed macrodomain-fused SirTM 1 (Mfs1). SirTMs are predominantly present in pathogenic organisms including strains in the bacterial *Clostridiaceae*, *Enterococcaceae*, *Lachnospiraceae*, *Spirochaetaceae*, and *Veillonellaceae* families and fungal *Aspergillus*, *Candida*, *Entamoeba*, *Fusarium*, and *Phytophthora* genera, among others (Figure 1A).

Composition of the Extended SirTM Operon in *Lactobacillales* and *Staphylococcaceae*

Bacterial SirTMs and their macrodomain partners are present as either independent operons or part of extended operons. One type of extended operon can be found in *Lactobacillales* and *Staphylococcaceae,* where the SirTMs are flanked by the ORFs of a glycine cleavage system H-like (GcvH-L) protein and a lipoate-protein ligase homolog (LplA2) (Figure 1B). LplAs are key enzymes for scavenging of the organosulfur cofactor lipoate (an alternative to the de novo synthesis of lipoate) and therefore are essential for virulence of microbial pathogens (Spalding and Prigge, 2010). Canonical GcvH is a component of the multienzyme glycine cleavage system (GCS) involved in glycine detoxification and one-carbon metabolism (Spalding and Prigge, 2010), where it serves as a reaction intermediate shuttle via a conserved lipoylated lysine. The genome of *Staphylococcus aureus* contains both a canonical GcvH associated with the GCS operon and a non-canonical form present in the extended pathogen-specific operon (Table 1). *Streptococcus pyogenes* lacks the GCS system (Spalding and Prigge, 2010 and this study) and its

Table 1. Comparison between the Lipoylation and Biotinylation Systems of *S. aureus* and *S. pyogenes*

		S. aureus		*S. pyogenes*	
		Protein	Accession[a]	Protein	Accession[a]
Lipoylation[b]	Lipoate scavenging	LplA1	CAG42736	LplA1	CAM30328
		LplA2[c]	CAG42075	LplA2[c]	CAM30198
		LplA3	CAG43266	–	–
		LplA4	CAG42323	–	–
	Lipoyl-carrier proteins	–	–	AcoC	CAM30333
		–	–	AcoL	CAM30332
		DBT	CAG43237	–	–
		DLAT	CAG42804	–	–
		DLST	CAG43130	–	–
		GcvH	CAG42548	–	–
		GcvH-L[c]	CAG42072	GcvH-L[c]	CAM30195
Biotinylation[d]	Biotin scavenging	BPL	BAB57618	BPL	CAM30079
	Biotin-carrier proteins	BCCP (ACC)	BAB57689	BCCP (ACC)	CAM29699
		–	–	BCCP (OAD)	CAM30229
		PC	BAB57276	–	–

ACC, acetyl-CoA carboxylase; AcoC, dihydrolipoamide acetyltransferase (AoDH-E2); AcoL, dihydrolipoamide dehydrogenase (AoDH-E3); BCCP, biotin carboxyl carrier protein; BPL, biotin protein ligase; DBT, dihydrolipoamide branched chain transacylase E2 (BCDH-E2); DLAT, dihydrolipoamide *S*-acetyltransferase (PDH-E2); DLST, dihydrolipoamide *S*-succinyltransferase (KDH-E2); OAD, oxaloacetate dehydrogenase; PC, pyruvate carboxylase.

[a]GenBank accession numbers.

[b]Based on Spalding and Prigge (2010).

[c]Operon-encoded proteins.

[d]Based on Fall (1979).

genome encodes only the SirTM-associated form of GcvH. This suggests that GcvH-L supports a GCS-independent function.

In addition, the SirTM operon is associated with two uncharacterized oxidoreductases, one of which exhibits similarities to a flavin-utilizing bacterial luciferase-like monooxygenases (LLM; Pfam: PF00296) and the other to an Old Yellow Enzyme (OYE; Pfam: PF00724) type NADH:flavin oxidoreductase (Figure 1B).

The LplA2/GcvH-L Pair: An Operon-Specific Lipoylation System

To explore the molecular and biological functions of the SirTMs, we performed a detailed biochemical characterization of the proteins from *S. pyogenes* (*Spy*SirTM) and *S. aureus* (*Sau*SirTM) as representatives of their respective families. Since these sirtuins are part of an operon encoding the LplA2/GcvH-L pair, we hypothesized that they catalyze delipoylation of GcvH-L. Therefore, we first assessed whether the operon-encoded LplA2 can lipoylate GcvH-L, as seen for the canonical LplA/GcvH pairs from *S. aureus* and *Escherichia coli* that served as positive controls. Indeed, both operonal LplA2s efficiently transferred lipoic acid (LA) onto GcvH-L as assessed by immunoblot (IB) using a specific anti-lipoylation antibody (Figures 2A, 2B, and S2B). Mutagenesis showed that the lipoyl-attachment site is Lys56 within the conserved Ex(2)Kx(10)G motif (Fujiwara et al., 1991; Spalding and Prigge, 2009). Since *Sau*GcvH-L appears to be weakly lipoylated in the absence of LplA2, we investigated whether the recombinant GcvH-L can be co-expressionally modified by the endogenous *E. coli* ligase. First, we performed lipoylation assays testing the ability of *Sau*LplA1, 2 or *Eco*LplA to modify the corresponding GcvH proteins (Figure S2C). While *Sau*LplA2 shows selectivity for GcvH-L, *Sau*LplA1 appears to be indiscriminative toward both *S. aureus* GcvHs. Both enzymes, however, exhibit negligible activity toward *Eco*GcvH. In contrast, *Eco*LplA readily modifies all three GcvHs. To test whether these results are transferable to the expression conditions, we supplemented cultures upon induction of protein expression with LA (Figure 2C). Indeed, this increased the co-expressional lipoylation of GcvH-L. The effect could further be enhanced via inhibition of protein synthesis by kanamycin (doubly treated GcvH-L is termed "in vivo lipoylated" in subsequent experiments).

Together these results suggest that LplA2 is a specific lipoate ligase for GcvH-L and that the LplA2/GcvH-L pair shares several characteristics with the LplA/GcvH pairs of the GCS.

SirTMs Lack Protein Deacylase Activity

Next, we tested whether *Sau*SirTM was able to reverse the lipoylation of *Sau*GcvH-L. We performed the assay with in vitro and in vivo lipoylated *Sau*GcvH-L and showed that *Sau*SirTM failed to delipoylate *Sau*GcvH-L (Figure 3A). On the other hand, incubation with lipoamidase (Lpa) from *Enterococcus faecalis* readily removed the lipoyl moiety (Figure S2D).

Since lipoate is a limiting factor for the growth of some pathogenic bacteria, we hypothesized that the SirTMs could act as scavenging enzymes by delipoylating other endogenous or host-derived proteins. To assess this, we performed delipoylation assays using total protein extracts from *S. aureus*, *E. coli*, and

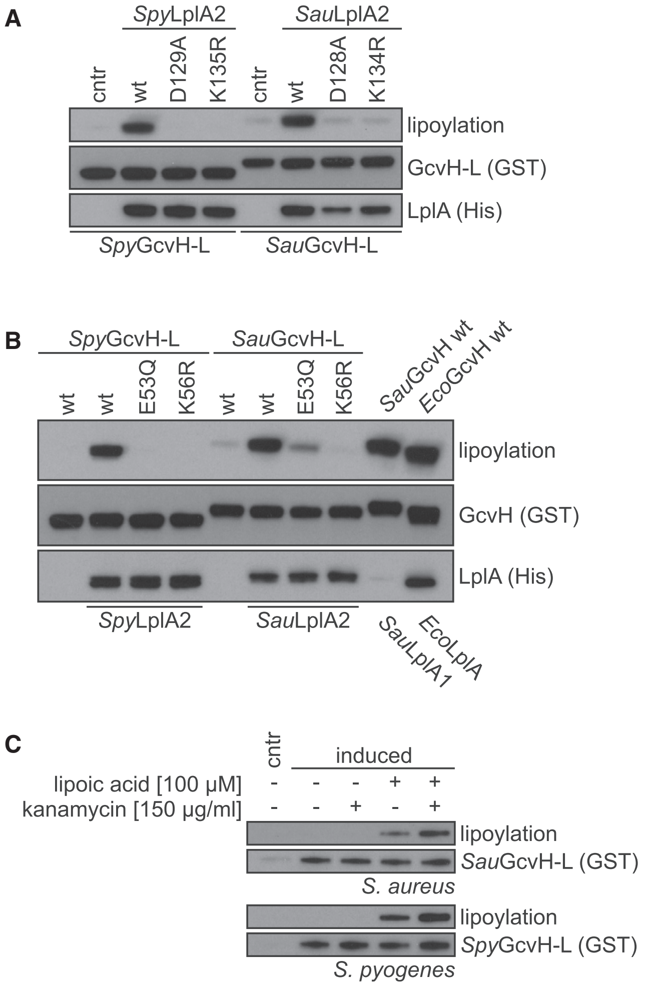

Figure 2. The LplA2/GcvH-L Pair Resembles Its Canonical Homologs

(A) Wild-type, but not mutant LplA2, can lipoylate the GcvH-L protein. The mutations were chosen in analogy to the canonical LplA/GcvH pair of *E. coli* where they interfere both with the lipoate adenylation and the subsequent lipoate transfer (Fujiwara et al., 2010).

(B) Mutations of lipoylation motif residues within GcvH-L impair the lipoate transfer reaction. Mutation of Lys56 interferes with lipoyl attachment, whereas Glu53 is important for recognition by LplA2 (Fujiwara et al., 1991, 2010). Control reactions were carried out using the canonical LplA/GcvH pairs of *S. aureus* (*Sau*GcvH, *Sau*LplA1) and *E. coli* (*Eco*GcvH, *Eco*LplA).

(C) *Sau*GcvH-L and *Spy*GcvH-L were expressed in the presence and absence of lipoic acid supplementation. In addition, protein synthesis of some samples was interrupted by supplementation with kanamycin 1 hr prior to culture harvesting. The effect of the additives on GcvH-L lipoylation was assessed by immunoblot. For further characterization of the LplA2/GcvH-L pair, see Figure S2.

human 293T cells as sources of lipoylated protein substrates (Figures 3B, 3C, and S3A). However, we were unable to detect any delipoylation. Similarly, by using HRP-conjugated streptavidin to detect protein biotinylation in the same protein extracts, we did not observe any deacylation activity toward this evolutionary related modification either (Figures 3B, 3C, and S3A). To eliminate the possibility that extract components interfere with the debiotinylation activity of SirTMs, we performed debiotinylation assays with in vitro modified biotin carboxyl carrier proteins (BCCPs), the only biotinylated proteins shared between *S. aureus* and *S. pyogenes* (Table 1; Figure 3D). Again, SirTMs showed no activity against biotinyl-BCCP. These observations suggest that SirTMs do not participate in lipoyl or biotinyl scavenging/removal. This is further supported by our observation that LplA2 can utilize lipoamide, which mimics protein bound LA, as substrate for GcvH-L modification and therefore release of the free LA by scavenging appears not to be required for LplA2 function (Figure S2E).

We extended our deacylation analyses to two other prominent lysine modifications in bacteria—acetylation and succinylation—using IBs with modification-specific pan-antibodies as detection tools. To exclude interference from the endogenous bacterial sirtuin, CobB, we performed the deacetylation assays on the cell extracts of CobB-deficient *E. coli* (BL21(DE3)Δ*CobB*) (Figure 3E). Our results show that SirTMs are unable to remove acetylation from endogenous proteins, whereas the control enzymes *S. aureus* CobB (*Sau*CobB) and human SIRT2 show activity against some of the acetylated cellular proteins. We also performed two additional in vitro deacetylation assays using acetylated histones and p53-derived peptides, both considered to be generic sirtuin targets. Under the assay conditions, neither SirTM displayed any deacetylation activity toward acetylated p53 (Figure 3F) or penta-acetylated histone H3 (Figure 3G). In contrast, both control enzymes, SIRT2 and *Sau*CobB, showed robust deacetylation activity toward these targets. Desuccinylation activity was tested using a fluorescence succinyl-peptide assay as well as non-enzymatically succinylated histone octamers as substrates. Again, we could not detect any activity for the SirTMs against these targets (Figures S3B and S3C).

Taken together, these results suggest that the pathogenic SirTMs lack deacylation activity.

Class M Sirtuins Are Lipoylation-Dependent ADP-Ribosyltransferases

While deacylation is the predominant activity of sirtuins (Du et al., 2009), several studies have reported that some sirtuins also exhibit ADP-ribosyltransferase activity (Haigis et al., 2006; Kowieski et al., 2008). Therefore, we decided to test whether *Sau*SirTM could ADP-ribosylate the purified operon proteins in the presence of ^{32}P-labeled NAD^{+}. We resolved the reaction products by SDS-PAGE and analyzed them by autoradiography and IB. Among the tested proteins, only *Sau*GcvH-L was modified by *Sau*SirTM (Figure 4A). Furthermore, we observed that prior lipoylation of *Sau*GcvH-L greatly stimulated ADP-ribosyl transfer (Figure 4A, right panel). We further confirmed the presence of ADP-ribosyl group on GcvH-L by the treatment with phosphodiesterase NUDT16 that can hydrolyze protein bound ADP-ribose (Palazzo et al., 2015) (Figure S4A).

Crosstalk between Lipoylation and ADP-Ribosylation

To confirm the dependence of GcvH-L mono-ADP-ribosylation (MARylation) on lipoylation, we performed lipoylation assays using wild-type (WT) and lipoylation-deficient GcvH-L mutants followed by MARylation reactions using *Spy*SirTM and *Sau*SirTM (Figure 4B). Since only the lipoylated WT GcvH-L was MARylated we concluded that the reaction is dependent

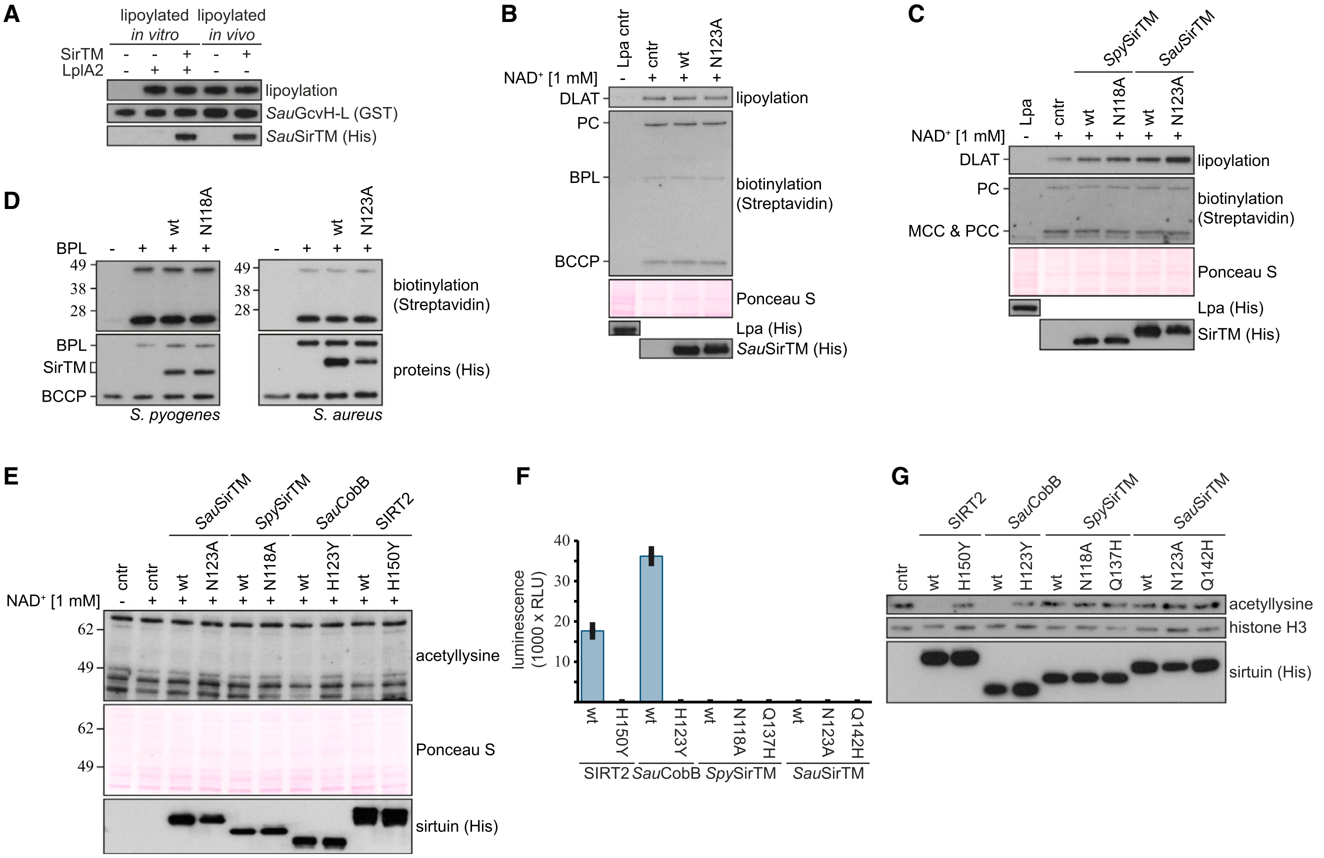

Figure 3. Class M Sirtuins Lack Deacylase Activity

(A) Delipoylation assay performed with *Sau*SirTM on in vitro and in vivo lipoylated *Sau*GcvH-L. The in vitro lipoylation was carried out for 30 min prior to addition of SirTM and NAD$^+$. Control samples with in vivo lipoylated *Sau*GcvH-L were treated as "in vitro" samples, however, without addition of *Sau*LplA2 and LA.

(B) Delipoylation and debiotinylation assay performed on *S. aureus* cell extracts. For lipoylation only, dihydrolipoamide *S*-acetyltransferase (DLAT) could be detected, whereas BCCP, biotin protein ligase (BPL), and pyruvate carboxylase (PC) could be identified as biotinylated. For assays on *E. coli* cell lysates, see Figure S3A.

(C) Delipoylation and debiotinylation assays were performed as in (B), but using human 293T cell extract. For lipoylation only DLAT could be detected, whereas PC, 3-methylcrotonyl-CoA carboxylase (MCC) and propionyl-CoA carboxylase (PCC) could be identified as biotinylated.

(D) Debiotinylation assay using recombinant, in vitro modified BCCP as substrate. Free biotin was removed from the initial biotinylation reaction by passing it twice over a desalting column.

(E) Deacetylation activity of SirTMs was tested on BL21(DE3)Δ*CobB* lysates. Human SIRT2 (isoform 2) and *Sau*CobB were used as positive controls.

(F) Deacetylase activities of *Sau*SirTM and *Spy*SirTM against a p53-derived peptide were assessed using the SIRT-Glo assay (Promega). Data are background corrected means ± SD of triplicate measurements.

(G) Deacetylase activity of sirtuins (compare to F) was tested against penta-acetylated histone H3 (modified residues: K4, K9, K14, K18, and K23).

on prior lipoylation. To rule out that free LA stimulates the reaction, we performed a MARylation assay following incubation with LA and lipoamide (Figure S4B). MARylation was only observed following covalent linkage of lipoic acid to GcvH-L, thus strengthening the notion that the protein modification rather than free LA is the determining factor. To distinguish between an allosteric stimulation of ADP-ribosylation or a more direct involvement in the reaction, we supplemented MARylation reactions of unmodified GcvH-L with lipoylated peptides (Figure S4C). While lipoylated GcvH-L was readily modified, neither of the lipoylated peptides stimulated ADP-ribosylation activity toward the unmodified GcvH-L, thus suggesting that ADP-ribosylation can only occur as consequence of a prior lipoylation of the same GcvH-L molecule. Analysis of the peptides incubated in the presence of SirTM and NAD$^+$ by thin layer chromatography (TLC) showed no incorporation of ADP-ribose (ADPr) into the peptides, hence excluding a competition reaction between peptide and unmodified GcvH-L (Figure S4D). To assess whether the SirTM-mediated ADP-ribosylation is specific for GcvH-Ls, we performed MARylation assays using the lipoylated canonical GcvHs from *S. aureus* and *E. coli* (Figure 4C) as well as biotinylated BCCP as substrates (Figure S4E). Strikingly, SirTMs show absolute selectivity for the lipoylated GcvH-L. Altogether, these data show that lipoylated, full-length GcvH-L is required as substrate in the ADP-ribosylation reaction.

To rule out that the observed ADP-ribosylation is attributed to an indirect non-enzymatic process or represents a weak side

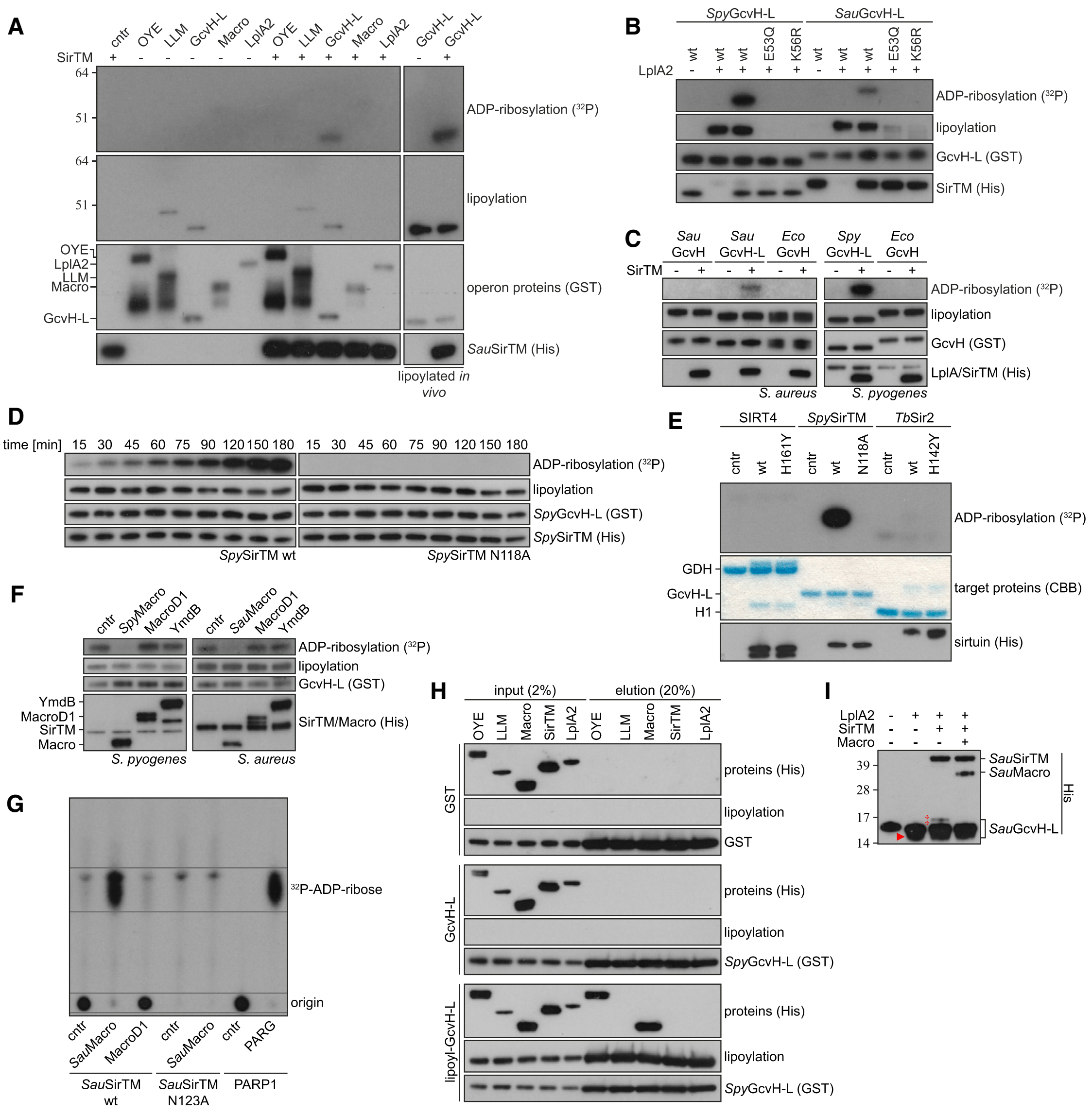

Figure 4. Crosstalk between Lipoylation and ADP-Ribosylation: The ADP-Ribosylation of GcvH-L by SirTM Is Lipoylation-Dependent

(A) Mono-ADP-ribosylation assays of operon and associated proteins were performed with GST-tagged target proteins derived from *S. aureus* (indicated on top). Proteins were incubated in the presence and absence of *Sau*SirTM. In addition to the Apo-proteins, in vivo lipoylated GcvH-L was tested (right). To control for self-modification of *Sau*SirTM the enzyme was incubated in the absence of target proteins (cntr). The lipoylated protein observed in the LLM samples corresponds by apparent molecular mass to co-purified *E. coli* DLST.

(B) MARylation assays were performed with WT and lipoylation-deficient mutants of GcvH-L. The lipoylation reaction was carried out for 30 min prior to addition of SirTM and NAD$^+$.

(C) Cross-MARylation assays performed using canonical GcvHs from *S. aureus* and *E. coli* as putative target proteins.

(D) Wild-type, but not the catalytic mutant N118A, of *Spy*SirTM modifies lipoylated GcvH-L in a time-dependent manner.

(E) Comparison of the ADP-ribosyl transferase activities of human SIRT4ΔMTS and *T. brucei TmSir2* with *Spy*SirTM. The activities were assessed on previously described and herein identified substrates: glutamate dehydrogenase (GDH), histone H1 (H1), and GcvH-L for SIRT4, *Tm*Sir2 and *Spy*SirTM, respectively. Deacetylase activity of *Tm*Sir2 was controlled using a p53-derived peptide as substrate (Figure S3D).

(F) ADP-ribosyl hydrolase assays were performed with the operon macrodomains (*Spy*Macro and *Sau*Macro). Control reactions were carried out with the closely related macrodomain proteins MacroD1 (human) and YmdB (*E. coli*) or in the absence of a macrodomain protein (cntr).

(G) Identification of the Macro reaction product by TCL. Protein bound ^{32}P-ADPr is immobile under the TLC condition (origin), whereas released ^{32}P-ADPr co-migrates with the PARP1/PARG (human) control.

(H) GST-pulldown assay using GST-fused *Sau*GcvH-L as bait. Prior to pull-down *Sau*GcvH-L was either lipoylated or Lpa treated to define the modification status. The molar ratio of proteins (GcvH-L/GST:operonal) was 1:1. Recombinant GST was used as negative control and binding was monitored by IB.

(legend continued on next page)

reaction, we performed a number of validation assays. First, we confirmed that ^{32}P-NAD^{+} is stable under the assay condition, thus ruling out that hydrolyzed NAD products could cause non-enzymatic ADP-ribosylation (Figure S4F). We also performed a time course experiment comparing WT *Spy*SirTM with a catalytically inactive mutant (N118A) (Yuan and Marmorstein, 2012). While the WT enzyme transferred the radiolabel in a time-dependent manner, no transfer was observed for the catalytic mutant (Figure 4D). We could further confirm that a genuine catalytic activity was required for the ADPr transfer by performing the assays in presence of the general sirtuin inhibitors nicotinamide and Tenovin-6 (Figure S4G). While Tenovin-6 inhibited the GcvH-L MARylation, nicotinamide did not significantly affect the reaction. The latter finding is surprising and might indicate that the active site is protected from base-exchange and thus from inhibition by nicotinamide (Sauve, 2010).

Next, we compared the activity of *Spy*SirTM with human SIRT4 and *Trypanosoma brucei Tb*Sir2 that both have described activity as ADP-ribosyl transferases (Fahie et al., 2009; Haigis et al., 2006). While *Spy*SirTM robustly ADP-ribosylates GcvH-L, neither SIRT4 nor *Tb*Sir2 exhibits comparable activity on their described targets (glutamate dehydrogenase and histone H1, respectively) using the same enzyme and substrate concentrations (Figure 4E).

Together, these results provide evidence for a highly specific ADP-ribose transferase activity of SirTMs that depends on the prior lipoylation of the target protein GcvH-L.

Operon Macrodomains Specifically Reverse SirTM-Mediated ADP-Ribosylation

As some macrodomain-containing proteins are known to hydrolyze protein ADP-ribosylation (Jankevicius et al., 2013; Slade et al., 2011; Sharifi et al., 2013), we assessed the ability of the operon-encoded macrodomains to remove the SirTM-mediated MARylation of the GcvH-L. Strikingly, the operon macrodomains (*Spy*Macro and *Sau*Macro) catalyzed efficiently the modification reversal, as indicated by the loss of radiolabel from GcvH-L, whereas the homologous protein de-MARylating macrodomains from human and *E. coli* (MacroD1 and YmdB, respectively) did not exhibit this activity (Figures 4F and S4H). We further characterized the reaction product of the Macro reaction by TLC. While protein bound ADPr is immobile, the Macro cleavage product co-migrates with ADPr released in the human PARP1/PARG control reaction (Figure 4G) (Slade et al., 2011). The activity of the operonal macrodomains is not absolutely dependent on the lipoylation present on GcvH-L, as revealed by testing the de-MARylation reactions using Lpa-delipoylated GcvH-L as a substrate (Figure S4I). However, GST-pull down assays showed that the macrodomain interacts with GcvH-L in a strictly lipoylation-dependent manner (Figure 4H), thus suggesting lipoylation-dependence in vivo.

Since GcvH-L ADP-ribosylation is part of a coupled reaction, we tested the labeling efficiency within the assays. To this end, all operon reactions were performed consecutively with samples taken after completion of each reaction and subsequently analyzed by electrophoretic gel shift. While lipoylation increased the electrophoretic mobility (Ali et al., 1990), MARylation led to a distinct retention (Figure 4I). We estimate that under our assay conditions, ~50% of the GcvH-L protein is lipoylated and 5% of the initial substrate becomes ADP-ribosylated. Importantly, MARylation can be fully reversed by addition of *Sau*Macro.

Overall, we conclude that the operon-encoded Macro protein specifically reverses SirTM-dependent ADP-ribosylation and its reaction product is ADP-ribose as seen for other macrodomain proteins (Jankevicius et al., 2013; Sharifi et al., 2013). We propose that the pathogenic SirTM/Macro pair coevolved as an ON/OFF switch for the GcvH-L ADP-ribosylation.

*Spy*SirTM Structure Reveals an Unexpected Catalytic Residue

To gain further insight into the SirTM catalyzed ADP-ribosylation, we determined the crystal structure of *Spy*SirTM, both in the ligand-free form as well as bound to ADPr or NAD^{+}. The overall fold for these three structures is identical with a low root-mean-square deviation (RMSD) (0.29–0.31 Å). The models contain residues 3–292 (residues 7–10 are not visible in the electron density maps) and follow a typical sirtuin fold comprised of a large Rossmann fold and a small bipartite zinc coordinating domain (Figure 5A; Table 2). *Spy*SirTM is closest in structure to sirtuins from *Thermotoga maritima* (*Tm*Sir2, PDB: 2H4F) and *Saccharomyces cerevisiae* (HST2, PDB: 1SZC) (Figures S5A and S5B).

The ligand-*Spy*SirTM complexes reveal that the general mode of NAD^{+} binding to *Spy*SirTM is similar to that observed in other sirtuins (Yuan and Marmorstein, 2012) (Figures S5A and S5B). No major conformational changes are observed upon binding of either ADPr or NAD^{+}, with the exception of slight readjustment of the loop region between $\beta 6$ and $\alpha 14$ as well as following $\beta 7$ (Figure 5B). This movement allows the highly conserved Asn258 to interact with the 2′- and 3′-OH groups of the adenine ribose, while the imidazole group of the relatively poorly conserved His259 rotates by ~110° to allow stacking against the adenine moiety. In addition, the pyrophosphate is coordinated by the class-specific GVGx[NT]TP motif (residues 229–235), found in the $\beta 6$–$\alpha 14$ loop.

The amide moiety of nicotinamide is bound by direct and water-mediated polar contacts with Ala34, Phe42, Ala119, and Asp120, thus positioning the nicotinamide and the distal ribose in a conformation similar to that observed for *Tm*Sir2 (Figure S5B). The conserved Asn118 coordinates a highly ordered water molecule, which was proposed to facilitate the initial hydrolysis of nicotinamide from NAD^{+} (Zhao et al., 2004). On the α-face of the ribose the 3′-OH group interacts with the absolutely conserved residue Gln137, which replaces the catalytic histidine found in all other sirtuin classes. Gln137 makes further contact with the N^{δ} atom of the similarly conserved residue Arg192

(I) ADP-ribosylation band-shift assay performed with (de)modified *Sau*GcvH-L. *Sau*GcvH-L was incubated consecutively with LplA2, SirTM, and Macro under standard assay conditions (lipoylation 30 min, MARylation 1 hr, and de-MARylation 1 hr). Samples were taken after each reaction step and untreated *Sau*GcvH-L served as control. Under the electrophoretic condition, lipoylation leads to an increased migration (►), while MARylation results in a discrete retention (‡). MARylation was confirmed by its reversibility by Macro treatment.
See also Figure S4.

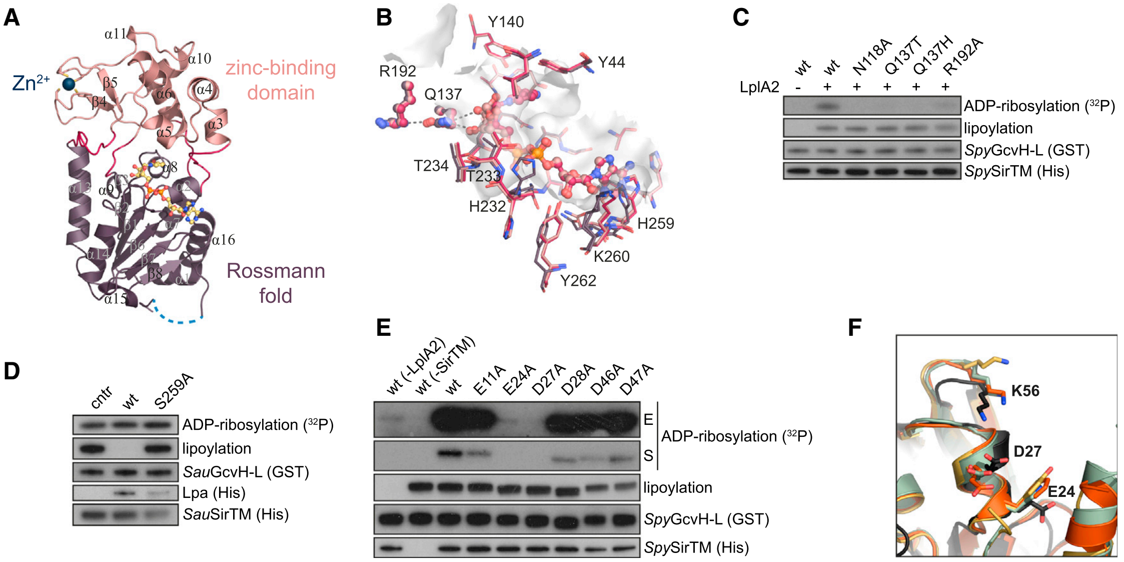

Figure 5. Structural Insights into ADP-Ribosylation by *Spy*SirTM

(A) Ribbon representation of the overall structure of *Spy*SirTM in complex with NAD$^+$ (yellow). The structure follows a typical sirtuin fold comprised of a Rossmann fold domain (purple) and a small bipartite zinc-coordinating domain (light red) tethered together by four loop regions (red). The zinc ion is indicated in blue and the unresolved loop region as a dotted line (light blue).

(B) Comparison of the binding sites of SirTM Apo (purple) in complex with ADPr (light red) or NAD$^+$ (red). The view corresponds to (A).

(C) The activity of selected *Spy*SirTM mutants was tested in a MARylation assay. *Spy*SirTM WT in the presence and absence of GcvH-L lipoylation were used as controls. For detailed information about the residues see Figure S1 and Table S1.

(D) Delipoylation assays were performed with in vivo lipoylated *Sau*GcvH-L (see also Figure 2C). The protein was MARylated with *Sau*SirTM and subsequently delipoylation was performed using WT or catalytically impaired (S259A) lipoamidase.

(E) MARylation assays were performed using selected GcvH-L mutants. To distinguish the effects of E24A and D27A short (S) and extended (E) autoradiographic exposures are shown.

(F) Comparison of residues involved in lipoyl attachment (K56) and MARylation (E24 and D27). *Spy*GcvH-L structure is shown in black and canonical GcvH of cattle (PDB: 3KLR), pea (PDB: 1DMX), and *M. tuberculosis* (PDB: 3HGB) in orange, green, and yellow, respectively. Residue numbers for GcvH-L are given. See also Figures S5 and S6.

(Figure 5B). Together, these interactions appear to be necessary for the correct positioning of the nicotinamide ribose, and by extension, the GcvH-L substrate. To assess the importance of these residues, we performed MARylation assays with WT and mutant *Spy*SirTM. While WT *Spy*SirTM modified *Spy*GcvH-L, a mutation of Asn118, Gln137, or Arg192 dramatically decreased the catalytic activity (Figure 5C). Interestingly, substitution of Gln137 with the general base histidine, found in other sirtuins, also leads to a complete loss of activity, suggestive of a distinct catalytic mechanism for class M sirtuins.

Relationship between MARylation and Lipoylation Sites on GcvH-L

To gain further insight into the requirements for the SirTM-mediated ADP-ribosylation, we characterized the ADPr attachment side on the GcvH-L. Since the lipoyl group contains a reactive 1,2-dithiolan moiety that, hypothetically, could be modified in a manner similar to cysteines, we incubated doubly modified *Sau*GcvH-L with WT and catalytically inactive Lpa to remove the lipoyl moiety (Figure 5D). While WT Lpa removed the lipoylation, *Sau*GcvH-L remained radiolabeled, thus indicating that the lipoyl moiety is not the ADPr attachment site.

Next, we tested whether Macro could remove ADP-ribosylation from acidic residues as shown for many of the characterized macrodomain enzymes using MARylated human PARP1 and PARP3 proteins as model substrates (Sharifi et al., 2013; Slade et al., 2011). In these assays, both operonal macrodomains showed comparable activity to human MacroD1 suggesting that they act on acidic residues (Figure S5C). Accordingly, we performed a mutagenesis analysis of highly conserved aspartates and glutamates within GcvH-L (Figures 5E, S6A, and S6B). All tested mutations showed robust lipoylation, but importantly E24A and D27A exhibited near and total loss of modification. Only extended exposure revealed slight ADP-ribosylation of E24A, whereas no ADPr incorporation could be detected for D27A. These findings suggest that Asp27 is the likely ADPr attachment side, but further indicates involvement of Glu24 in the ADP-ribosylation mechanism. It is worth noting, that the presence of an acidic residue in position of Asp27 is absolutely conserved in pathogenic GcvH-L proteins, but it is also common among canonical GcvH proteins. On the other hand, Glu24 is only present in GcvH-L homologs (Figures 5F, S5D, and S6B).

To better understand the spatial relationship between ADP-ribosylation and lipoylation attachment sites, we determined the crystal structure of *Spy*GcvH-L. The structure contains residues 1–110 and is comprised of a barrel-sandwich hybrid formed by two β sheets flanked by short α helices (Table 2). In comparison with previously solved H-proteins (RMSDs for all C$^\alpha$ atoms

Table 2. Crystallographic Data Collection and Refinement

	Value(s)[a] for			
	*Spy*SirTM			*Spy*GcvH-L
	Apo	ADPr Complex	NAD^+ Complex	Apo
Data Collection Statistics				
Wavelength (Å)/beamline	0.92000/I04-1	0.97625/I03	0.97625/I03	0.92000/I04-1
Detector	Pilatus 2M	Pilatus3 6M	Pilatus3 6M	Pilatus 2M
Space group	*P*1	*P*1	*P*1	$P3_1$ 2 1
Unit Cell				
a (Å)	34.16	33.94	33.88	64.12
b (Å)	41.51	41.52	41.48	64.12
c (Å)	51.23	51.25	51.26	73.04
α (°)	99.87	99.76	99.55	90.00
β (°)	94.63	93.40	93.61	90.00
γ (°)	90.77	92.66	92.98	120.00
Content of asymmetric unit	1	1	1	1
Resolution (Å)	34.81–1.54	29.35–1.90	40.82–2.03	55.53–1.50
	(1.58–1.54)	(1.94–1.90)	(2.08–2.03)	(1.53–1.50)
R_{sym} (%)[b]	5.9 (54.8)	7.4 (32.6)	14.6 (60.1)	3.9 (57.0)
I/σ(I)	10.6 (2.0)	8.0 (4.5)	4.8 (1.9)	27.1 (2.9)
Redundancy	3.8 (3.8)	2.4 (2.2)	2.3 (2.3)	8.5 (6.2)
Completeness (%)	88.3 (82.8)	92.1 (88.9)	97.2 (95.2)	99.3 (93.0)
Number of unique reflections	36,095 (2,501)	19,940 (1,296)	17,229 (1,263)	28,124 (1,293)
Refinement Statistics				
R_{cryst} (%)[c]	15.0	16.3	17.6	15.4
R_{free} (%)[d]	20.0	21.6	23.3	17.1
RMSD bond length (Å)	0.016	0.011	0.008	0.021
RMSD bond angle (°)	1.42	1.45	1.18	1.55
Average B Factor ($Å^2$)				
Protein	17.9	13.8	24.6	22.0
Water	32.1	24.6	32.6	39.6
Zn^{2+}	18.9	16.9	25.5	
ADPr	–	14.8	–	
NAD^+	–	–	24.5	
EDO/GLY/ALA/1PE	38.1/50.7/–/–	35.2/39.8/43.8/–	45.4/51.9/–/–	–/–/–/46.8

[a]Data for the highest resolution shell are given in parentheses.
[b]R_{sym} = Σ|/− </> |/Σ/, where / is measured density for reflections with indices *hkl*.
[c]R_{cryst} = Σ||Fobs| − |Fcalc||/Σ|Fobs|.
[d]R_{free} has the same formula as R_{cryst}, except that calculation was made with the structure factors from the test set.

ranging from 0.575–1.14 Å), the most striking differences observed in *Spy*GcvH-L are (1) the position of the β4/β5 loop (residues 38–40), which diverges from the consensus path by ~6 Å as a consequence of the presence of Arg72 in the β8 strand (a position usually occupied by Val or Ile); and (2) the absence of a C-terminal α helix (Figure S5D). Intriguingly, the missing α helix, which is held in place by electrostatic contacts between four conserved residues in the canonical GcvHs, would occlude residues implicated in ADP-ribose attachment in *Spy*GcvH-L (Glu24 and Asp27) (Figures S5E and S6B). The absence of the steric hindrance imposed by this α helix may be a prerequisite for the GcvH-L/SirTM interaction and thus could account for the high substrate specificity of SirTMs.

SirTM Function Is Linked to Redox Response

We observed that the operonal oxidoreductase *Spy*OYE shows robust, strictly lipoylation-dependent interaction with GcvH-L, as judged by in vitro pull-down assays (Figure 4H). The observation of a direct interaction with an oxidoreductase prompted us to further investigate whether SirTM activity could be linked to the oxidative stress response in vivo. For this, we utilized the pathogenic fungus *Candida albicans* as a model system and created a homozygous deletion strain of the gene encoding the SirTM homolog (*mfs1*, *orf19.2285*) (Figure S7). Upon inactivation of Mfs1 activity, fungal growth improved in the presence of high doses of hydrogen peroxide, as assayed on agar plates (Figure 6A). Improved growth was observed in four independent *mfs1Δ*

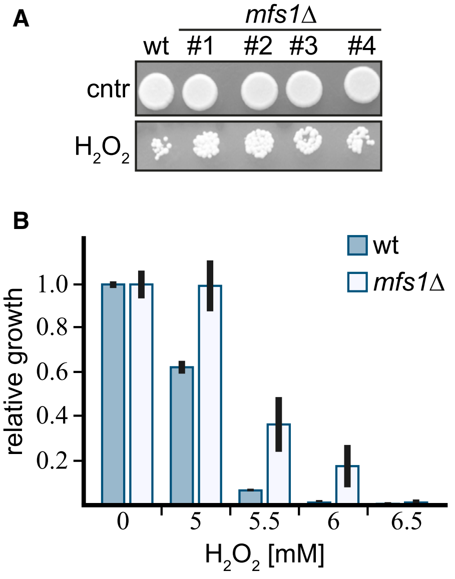

Figure 6. SirTM Function Is Linked to Oxidative Stress Responses

(A) Wild-type *C. albicans* and *mfs1Δ* mutants were inoculated onto plates with or without 5 mM H_2O_2 and growth assayed after 2 days of incubation at 30°C. Four independent *mfs1* homozygous deletion clones (labeled #1–#4) were tested. Moderate protection from oxidative stress was observed for all four mutant clones.

(B) The extent of growth inhibition by H_2O_2 was quantified in 96-well plate-based broth assays (see Supplemental Experimental Procedures). Following a 24 hr incubation at 37°C, percentage of growth was calculated relative to control (no H_2O_2). The experiment was performed independently twice, in biological duplicates (two independent colonies per strain). A similar trend was observed in both experiments. Shown are averages of growth inhibition ± SD for one of the experiments.

See also Figure S7.

clones (Figure 6A), and the same trend was observed when we analyzed growth in response to oxidative stress in suspension cultures (Figure 6B). This finding suggests that Mfs1 modulates the oxidative stress response in *C. albicans*.

DISCUSSION

In the present study, we have identified a distinct class of sirtuins (SirTMs). This class is highly conserved and occurs either as part of a bacterial operon or, in fungi, as a macrodomain-SirTM fusion enzyme.

SirTMs: Not Deacylases, but ADP-Ribosyl Transferases

Our results show that SirTMs do not possess any appreciable deacylation activity against a number of different endogenous and host-derived substrates (Figures 3 and S3). In accordance with the functional analysis, our sequence and structural analysis revealed the absence of a catalytic histidine residue, crucial for the proposed deacylase mechanism and found in all other hitherto described sirtuins (Sauve, 2010; Yuan and Marmorstein, 2012) and that may explain the apparent absence of deacylation activity in SirTMs. On the other hand, our data show a robust MARylation activity by SirTMs that is highly specific for the GcvH-L, a protein encoded by the same operon. Furthermore, the SirTM-dependent ADP-ribosylation is specifically and efficiently reversed by the pathogenic macrodomain proteins. Collectively, our data argue for protein ADP-ribosyl transferase as the primary activity of SirTMs.

Operon Proteins in the Response to Oxidative Stress

The data presented here strongly support the notion that ADP-ribosylation of GcvH-L is dependent on its prior lipoylation. The operon-encoded LplA2/GcvH-L pair is only distantly related to the canonical GCS, but nonetheless, the catalytic and the lipoate attachment residues are conserved in the proteins from pathogenic species. While *S. pyogenes* naturally lacks a canonical GCS, *S. aureus* possesses a complete GCS as well as the operon-encoded LplA2/GcvH-L pair (Spalding and Prigge, 2010 and this study). Interestingly, our analysis showed that the canonical *Sau*LplA1 can modify both GcvH proteins, while *Sau*LplA2 appears to have selectivity toward GcvH-L (Figure S2C). This may indicate that lipoylation of GcvH-L is the preferred target for LA attachment under conditions of operon activation.

Transcriptome and proteome analysis revealed that activation of this operon occurs under conditions of oxidative stress (Nobre and Saraiva, 2013; Palazzolo-Ballance et al., 2008; Surmann et al., 2014), consistent with an involvement in the oxidative stress response. This is further supported by several observations: (1) LA was shown to possess antioxidant properties in models of oxidative stress (Packer et al., 1995), (2) it was reported that *Mycobacterium tuberculosis* utilizes two components of the α-ketoacid dehydrogenase complex in a lipoyl-dependent defense against the oxidative immune response of the host (Bryk et al., 2002), and (3) the operon is genetically associated with the putative oxidoreductases LLM and OYE. Both enzymes are as yet uncharacterized. However, their families have been implicated in detoxification of ROS (Chaiyen et al., 2012; Odat et al., 2007), and we show that the oxidoreductase OYE directly interacts with lipoylated GcvH-L. These findings suggest that GcvH-L could act as carrier protein for the ROS scavenging lipoyl moiety and/or as a substrate for oxidoreductases. Evidence obtained from the fungal pathogen *C. albicans* in this study points toward an involvement of SirTMs in the oxidative stress response. Accordingly, in *C. albicans* the expression of *mfs1* is very low under standard growth conditions, and the gene is only expressed to a considerable degree upon oxidative stress either chemically induced (Enjalbert et al., 2009) or by exposure to human blood (Fradin et al., 2003). Collectively, these results are consistent with a primary cellular function of Mfs1 when cells encounter oxidative stress, such as upon host interactions. In line with a specific function in host-pathogen interactions, engulfment of *S. aureus* by host (human) cells led to an induction of the macrodomain encoded in the SirTM operon (Surmann et al., 2014).

Combining these observations together with the high prevalence of the SirTM operon in known pathogenic microorganisms and the importance of lipoate scavenging for microbial pathogenesis (Spalding and Prigge, 2010), we propose that the SirTM operon or SirTM-macrodomain fusion proteins in eukaryotic microbes modulate the response of microbial pathogens to oxidative stress and host-pathogen interactions.

Crosstalk between Lipoylation and ADP-Ribosylation

Here, we present the direct evidence for a connection between protein lipoylation and ADP-ribosylation. Collectively our data suggest that lipoylation of GcvH-L is a prerequisite for its MARylation. Since ADP-ribosylation is a known modification influencing protein function and interactions (Barkauskaite et al., 2013), it is interesting to speculate that the MARylation state of GcvH-L might regulate the availability of the lipoyl moiety for redox reactions. This idea is supported by our finding that the ADPr attachment site is in close structural proximity to the lipoylation site. Moreover, the specific upregulation of the operonal macrodomain in *S. aureus* after internalization into human cells (Surmann et al., 2014) indicates that under these conditions de-MARylation of GcvH-L prevails. Collectively, these data suggest that the ADP-ribosylation inhibits the interaction of the oxidoreductase with GcvH-L when it is not required. In other words, ADP-ribosylation of GcvH-L might be acting to keep the response "off" under non-stress conditions.

EXPERIMENTAL PROCEDURES

The experimental procedures are described in detail in the Supplemental Information. These procedures include plasmid construction, protein expression and purification, structure determination, interaction and enzymatic activity assays, cell culture condition, as well as phylogenetic analysis. Below is a simplified description of the major experimental procedures. The amino acid sequences of the operon proteins can be accessed through UniProtKB: under Q99WQ2, P67343, Q99WQ0, and Q99WP9 (*S. aureus*) as well as A2REC0, A2REC1, A2REC2, and A2REC3 (*S. pygones*).

Protein Expression and Purification

Recombinant proteins were expressed in Rosetta (DE3) cells and affinity purified either by Ni^{2+}-NTA chromatography (QIAGEN) or using glutathione Sepharose 4B (GE Life Sciences). All proteins were dialyzed over night against protein buffer (50 mM TrisHCl [pH 8], 200 mM NaCl, 1 mM DTT) (see Supplemental Information for further details). Protein purity was assessed by SDS-PAGE (Figure S2A).

Enzymatic Assays

Lipoylation reactions were carried out using 1 μM LplA and 2 μM target protein. Reactions were incubated for 30 min at 30°C before analysis by immunoblot or further processing. Control reactions were carried out in the absence of LplA.

Deacetylation of p53-derived peptide was tested with the SIRT-Glo assay kit (Promega) according to the manufacturer's recommendations using 6 nM sirtuin per reaction. Deacetylation of synthetic penta-acetylated histone H3 (Active Motif) was carried out as described earlier (Rack et al., 2014).

Delipoylation of in vitro and in vivo lipoylated GcvH-L was carried out in delipoylation buffer (50 mM TrisHCl [pH 8], 200 mM NaCl, 10 mM $MgCl_2$, 1 mM DTT) using 1 μM recombinant sirtuin, 1 μM GcvH1, and 1 mM NAD^+. Reactions were incubated at 30°C for 2 hr.

ADP-ribosylation reactions were carried out using 1 μM sirtuin, 1 μM target protein, 2 μCi ^{32}P-NAD^+, and 5 μM unlabeled NAD^+. Reactions were incubated for 60 min at 30°C before analysis by immunoblot or further processing. De-ADP-ribosylation assay was carried out using 1 μM radiolabeled GcvH-L and 1 μM macrodomain protein. The reactions were incubated for 1 hr at 30°C.

For further details regarding enzymatic assays, see Supplemental Information.

Crystallization

Recombinant proteins were concentrated to a concentration of ~14 mg/ml. Crystals were obtained by hanging-drop vapor diffusion at 293 K by mixing 120 nl protein solution with 30 nl seed stock and 150 nl precipitant. For the ADPr and NAD^+ complexes, native crystals were soaked with 1 mM ligand in mother liquor for 20 hr and 10 min, respectively. The crystals were cryoprotected in a solution of mother liquor plus 20% ethylene glycol and vitrified by submersion in liquid nitrogen for data collection.

A single *Spy*GcvH-L crystal grew at 293K using the sitting-drop vapor diffusion. The crystal was cryoprotected in a solution of precipitant plus 15% PEG400 (see Supplemental Information for further details).

X-Ray Data Collection, Processing, Structure Determination, Refinement, and Analysis

X-ray diffraction data were collected using synchrotron radiation (Table 2). The CCP4 software suite (Winn et al., 2011) was used to process all datasets, including integration, scaling, molecular replacement, density modification, automated model building, and structure refinement. COOT (Emsley and Cowtan, 2004) was used for manual model building and autoSHARP (Vonrhein et al., 2007) for locating the Se atoms in the Apo selenomethionine derivate as well as for refinement and initial phase calculation. The processing, phasing, and final refinement statistics are presented in Table 2 (see Supplemental Information for details).

All structural alignment were generated in COOT and figures prepared with PyMOL (Molecular Graphics System, Version 1.3 Schrödinger, LLC) using DSSP (Joosten et al., 2011) for secondary structure assignment.

ACCESSION NUMBERS

Atomic coordinates and structure factors have been deposited in the Protein Data Bank under ID codes PDB: 5A35, 5A3A, 5A3B, 5A3C.

SUPPLEMENTAL INFORMATION

Supplemental Information includes Supplemental Experimental Procedures, seven figures, and two tables and can be found with this article online at http://dx.doi.org/10.1016/j.molcel.2015.06.013.

AUTHOR CONTRIBUTIONS

J.G.M.R., R.M., R.K., and E.B. purified proteins and performed biochemical studies. E.B. and A.A. performed crystallography studies. Y.Q., D.C., R.M., A.Y.P., and A.T. performed in vivo studies. M.O., O.L., and I.M. performed supporting studies. J.G.M.R., D.L., and I.A. analyzed data. J.G.M.R. and I.A. wrote the manuscript.

ACKNOWLEDGMENTS

We thank Sean Prigge for providing us with the Lpa expression constructs, Cynthia Wolberger for the *Tb*Sir2 expression constructs, Manuel Cánovas for the BL21(DE3)Δ*CobB* strain, Tricia Lo for assistance with *C. albicans* experiments, Christoph Tang for the genomic DNA of *S. pyogenes* and help with the preparation of *S. aureus* cell extracts, and the beamline scientists at Diamond Light Source for support with data collection. We thank Susan Lea and Scott Williams for their advice as well as Karla Fejis and Ian Gibbs-Seymour for comments on the manuscript. The work in the A.T. laboratory was supported by Project Grant APP1023973 from the Australian National Health and Medical Research Council (NHMRC). Y.Q. was supported by a SuperScience Fellowship from the Australian Research Council. A.Y.P. is an NHMRC R.D. Wright Biomedical Fellow. The work in the I.A. laboratory is funded by the Wellcome Trust and the European Research Council.

Received: December 15, 2014
Revised: April 16, 2015
Accepted: June 4, 2015
Published: July 9, 2015

REFERENCES

Ali, S.T., Moir, A.J., Ashton, P.R., Engel, P.C., and Guest, J.R. (1990). Octanoylation of the lipoyl domains of the pyruvate dehydrogenase complex in a lipoyl-deficient strain of Escherichia coli. Mol. Microbiol. *4*, 943–950.

Asher, G., and Schibler, U. (2011). Crosstalk between components of circadian and metabolic cycles in mammals. Cell Metab. *13*, 125–137.

Barkauskaite, E., Jankevicius, G., Ladurner, A.G., Ahel, I., and Timinszky, G. (2013). The recognition and removal of cellular poly(ADP-ribose) signals. FEBS J. *280*, 3491–3507.

Bryk, R., Lima, C.D., Erdjument-Bromage, H., Tempst, P., and Nathan, C. (2002). Metabolic enzymes of mycobacteria linked to antioxidant defense by a thioredoxin-like protein. Science *295*, 1073–1077.

Chaiyen, P., Fraaije, M.W., and Mattevi, A. (2012). The enigmatic reaction of flavins with oxygen. Trends Biochem. Sci. *37*, 373–380.

Chen, D., Vollmar, M., Rossi, M.N., Phillips, C., Kraehenbuehl, R., Slade, D., Mehrotra, P.V., von Delft, F., Crosthwaite, S.K., Gileadi, O., et al. (2011). Identification of macrodomain proteins as novel O-acetyl-ADP-ribose deacetylases. J. Biol. Chem. *286*, 13261–13271.

Choi, J.E., and Mostoslavsky, R. (2014). Sirtuins, metabolism, and DNA repair. Curr. Opin. Genet. Dev. *26*, 24–32.

Dölle, C., Rack, J.G., and Ziegler, M. (2013). NAD and ADP-ribose metabolism in mitochondria. FEBS J. *280*, 3530–3541.

Du, J., Jiang, H., and Lin, H. (2009). Investigating the ADP-ribosyltransferase activity of sirtuins with NAD analogues and 32P-NAD. Biochemistry *48*, 2878–2890.

Du, J., Zhou, Y., Su, X., Yu, J.J., Khan, S., Jiang, H., Kim, J., Woo, J., Kim, J.H., Choi, B.H., et al. (2011). Sirt5 is a NAD-dependent protein lysine demalonylase and desuccinylase. Science *334*, 806–809.

Emsley, P., and Cowtan, K. (2004). Coot: model-building tools for molecular graphics. Acta Crystallogr. D Biol. Crystallogr. *60*, 2126–2132.

Enjalbert, B., Rachini, A., Vediyappan, G., Pietrella, D., Spaccapelo, R., Vecchiarelli, A., Brown, A.J., and d'Enfert, C. (2009). A multifunctional, synthetic Gaussia princeps luciferase reporter for live imaging of Candida albicans infections. Infect. Immun. *77*, 4847–4858.

Fahie, K., Hu, P., Swatkoski, S., Cotter, R.J., Zhang, Y., and Wolberger, C. (2009). Side chain specificity of ADP-ribosylation by a sirtuin. FEBS J. *276*, 7159–7176.

Fall, R.R. (1979). Analysis of microbial biotin proteins. Methods Enzymol. *62*, 390–398.

Feijs, K.L., Forst, A.H., Verheugd, P., and Lüscher, B. (2013). Macrodomain-containing proteins: regulating new intracellular functions of mono(ADP-ribosyl)ation. Nat. Rev. Mol. Cell Biol. *14*, 443–451.

Feldman, J.L., Baeza, J., and Denu, J.M. (2013). Activation of the protein deacetylase SIRT6 by long-chain fatty acids and widespread deacylation by mammalian sirtuins. J. Biol. Chem. *288*, 31350–31356.

Fradin, C., Kretschmar, M., Nichterlein, T., Gaillardin, C., d'Enfert, C., and Hube, B. (2003). Stage-specific gene expression of Candida albicans in human blood. Mol. Microbiol. *47*, 1523–1543.

Frye, R.A. (2000). Phylogenetic classification of prokaryotic and eukaryotic Sir2-like proteins. Biochem. Biophys. Res. Commun. *273*, 793–798.

Fujiwara, K., Okamura-Ikeda, K., and Motokawa, Y. (1991). Lipoylation of H-protein of the glycine cleavage system. The effect of site-directed mutagenesis of amino acid residues around the lipoyllysine residue on the lipoate attachment. FEBS Lett. *293*, 115–118.

Fujiwara, K., Maita, N., Hosaka, H., Okamura-Ikeda, K., Nakagawa, A., and Taniguchi, H. (2010). Global conformational change associated with the two-step reaction catalyzed by Escherichia coli lipoate-protein ligase A. J. Biol. Chem. *285*, 9971–9980.

Greiss, S., and Gartner, A. (2009). Sirtuin/Sir2 phylogeny, evolutionary considerations and structural conservation. Mol. Cells *28*, 407–415.

Haigis, M.C., Mostoslavsky, R., Haigis, K.M., Fahie, K., Christodoulou, D.C., Murphy, A.J., Valenzuela, D.M., Yancopoulos, G.D., Karow, M., Blander, G., et al. (2006). SIRT4 inhibits glutamate dehydrogenase and opposes the effects of calorie restriction in pancreatic beta cells. Cell *126*, 941–954.

He, W., Newman, J.C., Wang, M.Z., Ho, L., and Verdin, E. (2012). Mitochondrial sirtuins: regulators of protein acylation and metabolism. Trends Endocrinol. Metab. *23*, 467–476.

Jankevicius, G., Hassler, M., Golia, B., Rybin, V., Zacharias, M., Timinszky, G., and Ladurner, A.G. (2013). A family of macrodomain proteins reverses cellular mono-ADP-ribosylation. Nat. Struct. Mol. Biol. *20*, 508–514.

Joosten, R.P., te Beek, T.A., Krieger, E., Hekkelman, M.L., Hooft, R.W., Schneider, R., Sander, C., and Vriend, G. (2011). A series of PDB related databases for everyday needs. Nucleic Acids Res. *39*, D411–D419.

Kowieski, T.M., Lee, S., and Denu, J.M. (2008). Acetylation-dependent ADP-ribosylation by Trypanosoma brucei Sir2. J. Biol. Chem. *283*, 5317–5326.

Nobre, L.S., and Saraiva, L.M. (2013). Effect of combined oxidative and nitrosative stresses on Staphylococcus aureus transcriptome. Appl. Microbiol. Biotechnol. *97*, 2563–2573.

Nordlund, S., and Högbom, M. (2013). ADP-ribosylation, a mechanism regulating nitrogenase activity. FEBS J. *280*, 3484–3490.

Odat, O., Matta, S., Khalil, H., Kampranis, S.C., Pfau, R., Tsichlis, P.N., and Makris, A.M. (2007). Old yellow enzymes, highly homologous FMN oxidoreductases with modulating roles in oxidative stress and programmed cell death in yeast. J. Biol. Chem. *282*, 36010–36023.

Packer, L., Witt, E.H., and Tritschler, H.J. (1995). alpha-Lipoic acid as a biological antioxidant. Free Radic. Biol. Med. *19*, 227–250.

Palazzo, L., Thomas, B., Jemth, A.S., Colby, T., Leidecker, O., Feijs, K.L., Zaja, R., Loseva, O., Puigvert, J.C., Matic, I., et al. (2015). Processing of protein ADP-ribosylation by Nudix hydrolases. Biochem. J. *468*, 293–301.

Palazzolo-Ballance, A.M., Reniere, M.L., Braughton, K.R., Sturdevant, D.E., Otto, M., Kreiswirth, B.N., Skaar, E.P., and DeLeo, F.R. (2008). Neutrophil microbicides induce a pathogen survival response in community-associated methicillin-resistant Staphylococcus aureus. J. Immunol. *180*, 500–509.

Peterson, F.C., Chen, D., Lytle, B.L., Rossi, M.N., Ahel, I., Denu, J.M., and Volkman, B.F. (2011). Orphan macrodomain protein (human C6orf130) is an O-acyl-ADP-ribose deacylase: solution structure and catalytic properties. J. Biol. Chem. *286*, 35955–35965.

Rack, J.G., VanLinden, M.R., Lutter, T., Aasland, R., and Ziegler, M. (2014). Constitutive nuclear localization of an alternatively spliced sirtuin-2 isoform. J. Mol. Biol. *426*, 1677–1691.

Sauve, A.A. (2010). Sirtuin chemical mechanisms. Biochim. Biophys. Acta *1804*, 1591–1603.

Sharifi, R., Morra, R., Appel, C.D., Tallis, M., Chioza, B., Jankevicius, G., Simpson, M.A., Matic, I., Ozkan, E., Golia, B., et al. (2013). Deficiency of terminal ADP-ribose protein glycohydrolase TARG1/C6orf130 in neurodegenerative disease. EMBO J. *32*, 1225–1237.

Slade, D., Dunstan, M.S., Barkauskaite, E., Weston, R., Lafite, P., Dixon, N., Ahel, M., Leys, D., and Ahel, I. (2011). The structure and catalytic mechanism of a poly(ADP-ribose) glycohydrolase. Nature *477*, 616–620.

Spalding, M.D., and Prigge, S.T. (2009). The amidase domain of lipoamidase specifically inactivates lipoylated proteins in vivo. PLoS ONE *4*, e7392.

Spalding, M.D., and Prigge, S.T. (2010). Lipoic acid metabolism in microbial pathogens. Microbiol. Mol. Biol. Rev. *74*, 200–228.

Surmann, K., Michalik, S., Hildebrandt, P., Gierok, P., Depke, M., Brinkmann, L., Bernhardt, J., Salazar, M.G., Sun, Z., Shteynberg, D., et al. (2014). Comparative proteome analysis reveals conserved and specific adaptation patterns of Staphylococcus aureus after internalization by different types of human non-professional phagocytic host cells. Front Microbiol *5*, 392.

Till, S., and Ladurner, A.G. (2009). Sensing NAD metabolites through macro domains. Front Biosci (Landmark Ed) *14*, 3246–3258.

Vonrhein, C., Blanc, E., Roversi, P., and Bricogne, G. (2007). Automated structure solution with autoSHARP. Methods Mol. Biol. *364*, 215–230.

Winn, M.D., Ballard, C.C., Cowtan, K.D., Dodson, E.J., Emsley, P., Evans, P.R., Keegan, R.M., Krissinel, E.B., Leslie, A.G., McCoy, A., et al. (2011). Overview of the CCP4 suite and current developments. Acta Crystallogr. D Biol. Crystallogr. *67*, 235–242.

Yuan, H., and Marmorstein, R. (2012). Structural basis for sirtuin activity and inhibition. J. Biol. Chem. *287*, 42428–42435.

Zhao, K., Harshaw, R., Chai, X., and Marmorstein, R. (2004). Structural basis for nicotinamide cleavage and ADP-ribose transfer by NAD(+)-dependent Sir2 histone/protein deacetylases. Proc. Natl. Acad. Sci. USA *101*, 8563–8568.

Molecular Cell, Volume *59*

Supplemental Information

Identification of a Class of Protein ADP-Ribosylating Sirtuins in Microbial Pathogens

Johannes Gregor Matthias Rack, Rosa Morra, Eva Barkauskaite, Rolf Kraehenbuehl, Antonio Ariza, Yue Qu, Mary Ortmayer, Orsolya Leidecker, David R. Cameron, Ivan Matic, Anton Y. Peleg, David Leys, Ana Traven, and Ivan Ahel

Supplemental Figures and Figure Legends

Figure S1. Distinctive features of class M sirtuins (related to Figure 1).

(A) Multiple sequence alignment generated with representative sequences for class M sirtuins. Conservation of sequences is given in grey scale with a cut-off of 30%. The secondary structure elements of *Spy*SirTM are given on top of the alignment. Note that helices α5 and α15 are broken due to gaps in the alignment (dotted lines). Sequence motifs characterizing the novel sirtuin class are given underneath the alignment indicated as follows: Cofactor binding motif GAGxSAx(2)Gx(2)Y, purple triangles; nicotinamide ribose loop T[ST]N[VA]D, blue circles; catalytic residues QG and R, red asterisks; first part of zinc finger Cx(3)C, grey-blue diamonds and β6-α14 loop GVGx[NT]TP, light-red squares (Table S1).

(B) Ribbon-surface representation of *Spy*SirTM bound to NAD^+ (yellow). The sequence motifs representative for the novel sirtuin class are highlighted in different colors (see (A) for coloring description). The position of the unresolved residues is indicated by a dashed line.

(C) Multiple sequence alignment comparing the novel class M sirtuins with the other sirtuin classes: class I (HST2 and SIRT1), class II (SIRT4), class III (SIRT5), class IV (*Atr*Sir2L1 and SIRT7) and class U (*Sau*CobB, *Tb*Sir2 and *Tm*Sir2) (compare also Figure 1A). Secondary structure elements of *Spy*SirTM (yellow) and HST2 (green) are given on top of the alignment and broken elements due to alignment gaps are indicated by dotted lines. Sequence motifs for class M sirtuins are indicated underneath the alignment as in (A). For sequence accession numbers see Table S2.

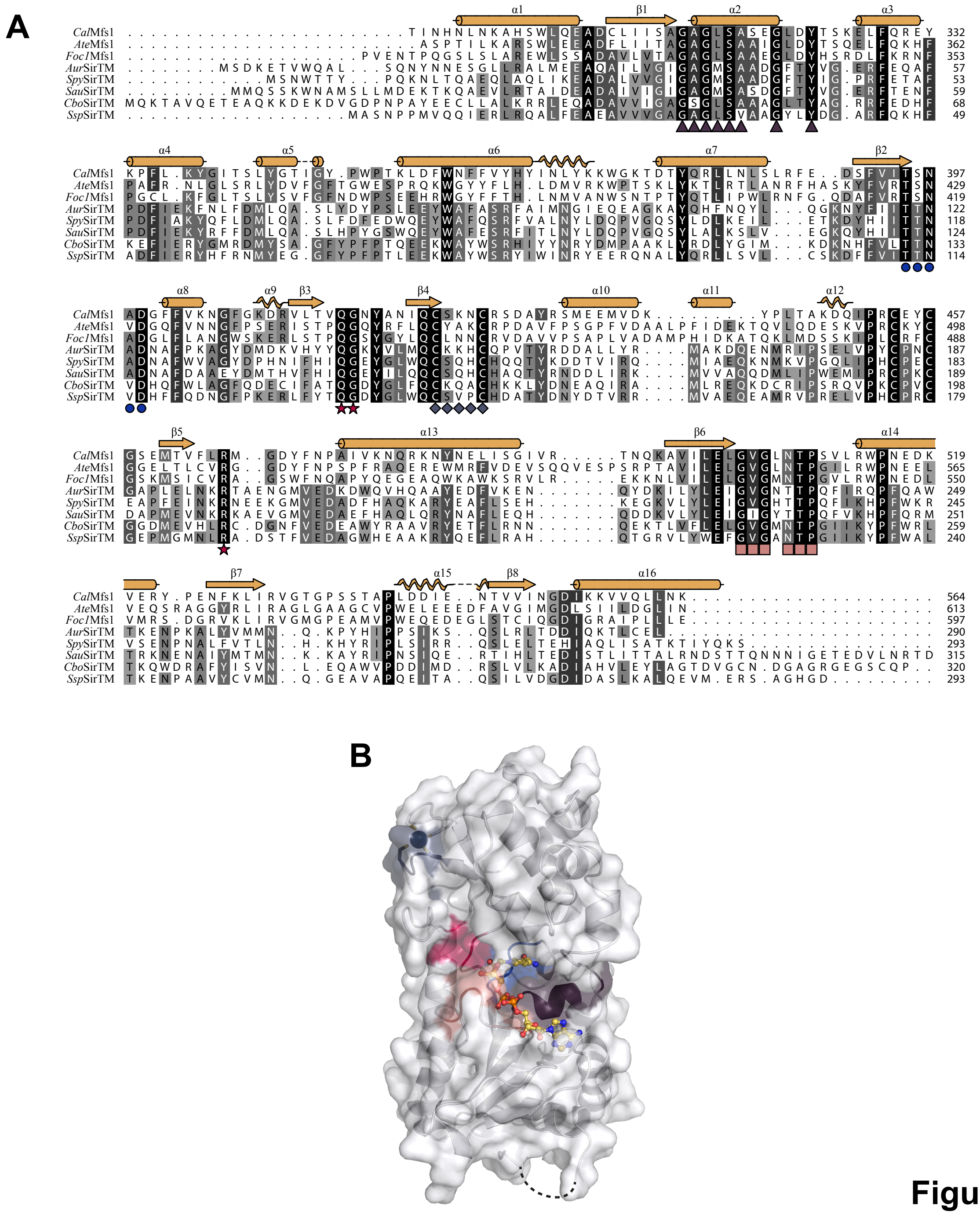
A
α1 β1 α2 α3
CalMfs1 TINHNLNKAHSWLQEADCLIISAGAGLSASEGLDYTSKELFQREY 332
AteMfs1 ASPTILKARSWLEEADFLIITAGAGLSAAIGLDYTSQELFQKHF 362
Foc1Mfs1 PVENTPQGSLSLAREWLSSADAVLVTAGAGLSAAEGLDYHSRDLFKRNF 353
AurSirTM MSDKETVWQAL...SQNYNNESGLLRALMEEAQAILVGIGAGMSAADGFTYVG.ERFEQAF 57
SpySirTM MSNWTTY...PQKNLTQAEQLAQLIKEADALVVGIGAGMSAADGFTYIG.PRFETAF 53
SauSirTM MMQSSKWNAMSLLMDEKTKQAEVLRTAIDEADAIVIGIGAGMSASDGFTYVG.ERFTENF 59
CboSirTM MQKTAVQETEAQKKDEKDVGDPNPAYEECLLALKRRLEQADAVVIGAGSGLSVSAAAGLTYAG.RRFEDHF 68
SspSirTM MASNPPMVQQIERLRQALFEAEAVVVGAGAGLSVAAGYLYDG.ARFQKHF 49
α4 α5 α6 α7 β2
CalMfs1 KPFL.KYGITSLYGTIGY.PWPTKLDFWNFFVYHYINLYKKWGKTDTYQRLLNLSLRFE..DSFVITSN 397
AteMfs1 PAFR.NLGLSRLYDVFGFTGWESPRQKWGYYFLH.LDMVRKWPTSKLYKTLRTLANRFHASKYFVRTSN 429
Foc1Mfs1 PGCL.KFGLTSLYSVFGFNDWPSEEHRWGYFFTH.LNMVANWSNTPTYQTLIPWLRNFG.QDAFVRTSN 419
AurSirTM PDFIEKFNLFDMLQA.SLYDYPSLEEYWAFASRFAIMNGIEQEAGKAYQHFNQYL...QGKNYFIITTN 122
SpySirTM PDFIAKYQFLDMLQA.SLFDFEDWQEYWAFQSRFVALNYLDQPVGQSYLDLKEIL...ETKDYHIITTN 118
SauSirTM PDFIEKYRFFDMLQA.SLHPYGSWQEYWAFESRFITLNYLDQPVGQSYLALKSLV...EGKQYHIITTN 124
CboSirTM KEFIERYGMRDMYSA.GFYPFPTQEEKWAYWSRHIYYNRYDMPAAKLYRDLYGIM...KDKNHFVLTTN 133
SspSirTM ADFIERYHFRNMYEG.GFYPFPTLEEKWAYWSRYIWINRYEERQNALYQRLLSVL...CSKDFFVITTN 114
α8 α9 β3 β4 α10 α11 α12
CalMfs1 ADGFFVKNGFGKDRVLTVQGNYANIQCSKNCRSDAYRSMEEMVDK.........YPLTAKDQIPRCEYC 457
AteMfs1 VDGQFVNNGFPSERISTPQGQYRFLQCYAKCRPDAVFPSGPFVDAALPFIDEKTQVLQDESKVPRCKYC 498
Foc1Mfs1 ADGLFLANGWSKERLSTPQGSYGYLQCLNNCRVDAVVPSAPLVADAMPHIDKATQKLMDSSKIPLCRFC 488
AurSirTM ADNAFPKAGYDMDKVHYYQGKYVLMQCCKKHCQPVTYRDDALLYR....MAKDQENMRIPSELVPYCPNC 187
SpySirTM ADNAFWVAGYDPHNIFHIQGEYGLWQCSQHCHQQTYKDDTVIRQ....MIAEQKNMKVPGQLIPHCPEC 183
SauSirTM ADNAFDAAEYDMTHVFHIQGEYILQQCSQHCHAQTYRNDDLIRK....MVVAQQDMLIPWEMIPRCPKC 189
CboSirTM VDHQFWLAGFQDECIFATQGDYGLFQCCKQACHKKLYDNEAQIRA....MLREQKDCRIPSRQVPKCPVC 198
SspSirTM VDHFFQDNGFPKERLFYTQGDYGLWQCSVPCHDATYDNYDTVRR....MVAEQEAMRVPRELVPHCPRC 179
β5 α13 β6 α14
CalMfs1 GSEMTVFLRM..GDYFNPAIVKNQRKNYNELISGIVR.....TNQKAVILELGVGLNTPSVLRWPNEDK 519
AteMfs1 GGELTLCVRG..GDYFNPSPFRAQEREWMRFVDEVSQQVESPSRPTAVILELGVGLNTPGILRWPNEEL 565
Foc1Mfs1 GSKMSICVRA..GSWFNQAPYQEGEAQWKAWKSRVLR.....EKKNLVILELGVGMNTPGVLRWPNEDL 550
AurSirTM GAPLELNKRTAENGMVEDKDWQVHQAAYEDFVKEN.......QYDKILYLEIGVGNTTPQFIRQPFQAW 249
SpySirTM EAPFEINKRNEEKGMVEDADFHAQKARYEAFLSEH.......KEGKVLYLEIGVGHTTPQFIKHPFWKR 245
SauSirTM DAPMEVNKRKAEVGMVEDAEFHAQLQRYNAFLEQH.......QDDKVLYLEIGIGYTTPQFVKHPFQRM 251
CboSirTM GGDMEVHLRC.DGNFVEDEAWYRAAVRYETFLRNN.......QEKTLVFLELGVGMNTPGIIKYPFWRL 259
SspSirTM GEPMGMNLRA.DSTFVEDAGWHEAAKRYQEFLRAH.......QTGRVLYWEFGVGANTPGIIKYPFWAL 240
β7 α15 β8 α16
CalMfs1 VERY.PENFKLIRVGTGPSSTAPLDDIE..NTVVINGDIKKVVQLLNK 564
AteMfs1 VEQSRAGGYRLIRAGLGAAGCVPWELEEEDFAVGIMGDLSIILDGLLIN 613
Foc1Mfs1 VMRS.DGRVKLIRVGMGPEAMVPWEQEDEGLSTCIQGDIGRAIPLLLE 597
AurSirTM TKENPKALYVMMN..Q.KPYHIPPSIKS..QSLRLTDDIQKTLCELL 290
SpySirTM VSENPNALFVTLN..H.KHYRIPLSIRR..QSLELTEHIAQLISATKTIYQKS 293
SauSirTM TRKNENAIYMTMN..K.KAYRIPNSIQE..RTIHLTEDISTLITTALRNDSTTQNNNIGETEDVLNRTD 315
CboSirTM TKQWDRAFYISVN..L.EQAWVPDDIMD..RSLVLKADIAHVLEYLAGTDVGCN.DGAGRGEGSCQP. 320
SspSirTM TKENPAAVYCVMN..Q.GEAVAPQEITA..QSILVDGGDIDASLKALQEVM.ERS.AGHGD 293
B

Figure S1

C

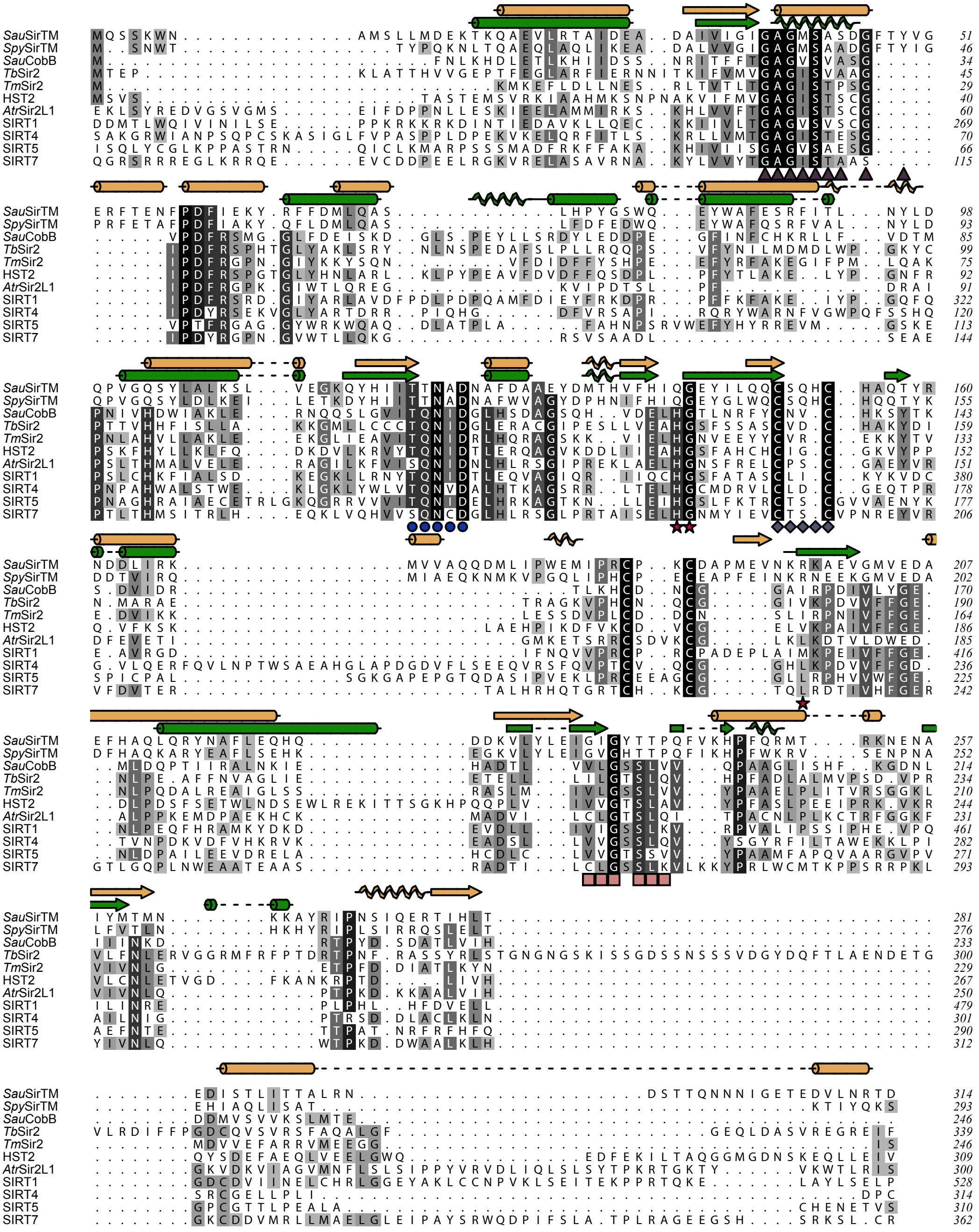
SauSirTM
SpySirTM
SauCobB
TbSir2
TmSir2
HST2
AtrSir2L1
SIRT1
SIRT4
SIRT5
SIRT7

Figure S1C

Figure S2. Specificity of GcvH-L lipoylation (related to Figure 2).

(A) Coomassie stained SDS-PAGE visualizing the purity of the operonal wild type proteins (4 µg protein per lane) after single affinity purification as used in biochemical assays. Mutant proteins were co-purified and of comparable purity.

(B) The lipoylation assay was performed with GST-tagged operon and associated proteins (indicated on top) derived from *S. aureus*. Proteins were incubated in the presence or absence of lipoic acid and the lipoylation status analyzed by IB. Note, lipoylated protein band in the LLM samples correspond in size to co-purified *E. coli* dihydrolipoamide *S*-succinyltransferase (DLST).

(C) Specificity of the lipoylation reactions of *S. aureus* LplA1, 2 and *Eco*LplA was assets using the canonical GcvHs and GcvH-L as substrates. Control reactions were carried out in the absence of LplA.

(D) *In vivo* lipoylated *Sau*GcvH-L (see Figure 2C) was incubated in the absence or presence of Lpa and the lipoylation status analyzed by IB.

(E) The ability of *S. aureus* LplA2 to utilize lipoic acid and lipoamide as substrates was tested by performing lipoylation reactions with increasing amounts of substrates (0.24, 2.4, 24 and 240 µM, respectively). Control reaction were carried out in the absence of LplA2 and substrate incubation was analyzed by IB.

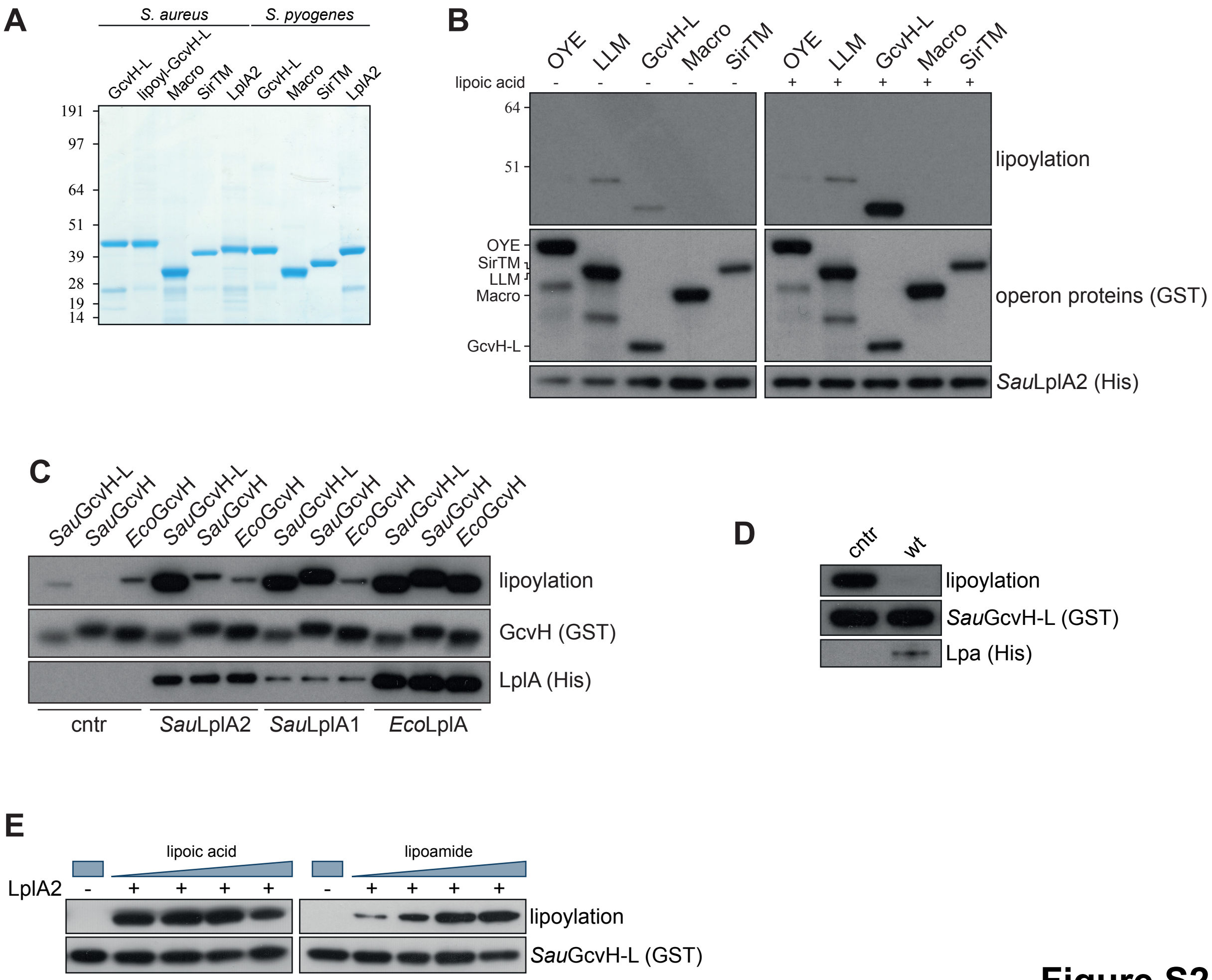
A
S. aureus
S. pyogenes
GcvH-L
lipoyl-GcvH-L
Macro
SirTM
LplA2
191
97
64
51
39
28
19
14
B
OYE
LLM
GcvH-L
Macro
SirTM
lipoic acid
64
51
lipoylation
OYE
SirTM
LLM
Macro
GcvH-L
operon proteins (GST)
SauLplA2 (His)
C
SauGcvH-L
SauGcvH
EcoGcvH
lipoylation
GcvH (GST)
LplA (His)
cntr
SauLplA2
SauLplA1
EcoLplA
D
cntr
wt
lipoylation
SauGcvH-L (GST)
Lpa (His)
E
lipoic acid
lipoamide
LplA2
lipoylation
SauGcvH-L (GST)

Figure S2

Figure S3. SirTMs lack deacylase activity (related to Figure 3 and 4).

(A) De-lipoylation and -biotinylation assay performed on *E. coli* BL21(DE3)Δ*CobB* cell extracts. For lipoylation modified DLAT and DLST could be detected, whereas BCCP could be identified as biotinylated.

(B) Desuccinylating activities of *Sau*SirTM and *Spy*SirTM against a succinyl-peptide were assessed using a fluorogenic SIRT5 assay (BPS Bioscience). Nicotinamide inhibition of SIRT5 and catalytic mutants were used as controls as indicated. Data are background corrected means ± SD of triplicate measurements.

(C) Desuccinylation assay performed with non-enzymatically succinylated histone octamers.

(D) Deacetylase activity of *Tb*Sir2 against p53 derived peptide was assets using the SIRT-Glo assay (Promega). A catalytic inactive mutant (H142Y) and nicotinamide inhibition were used as controls as indicated. Data are background corrected means ± SD of triplicate measurements.

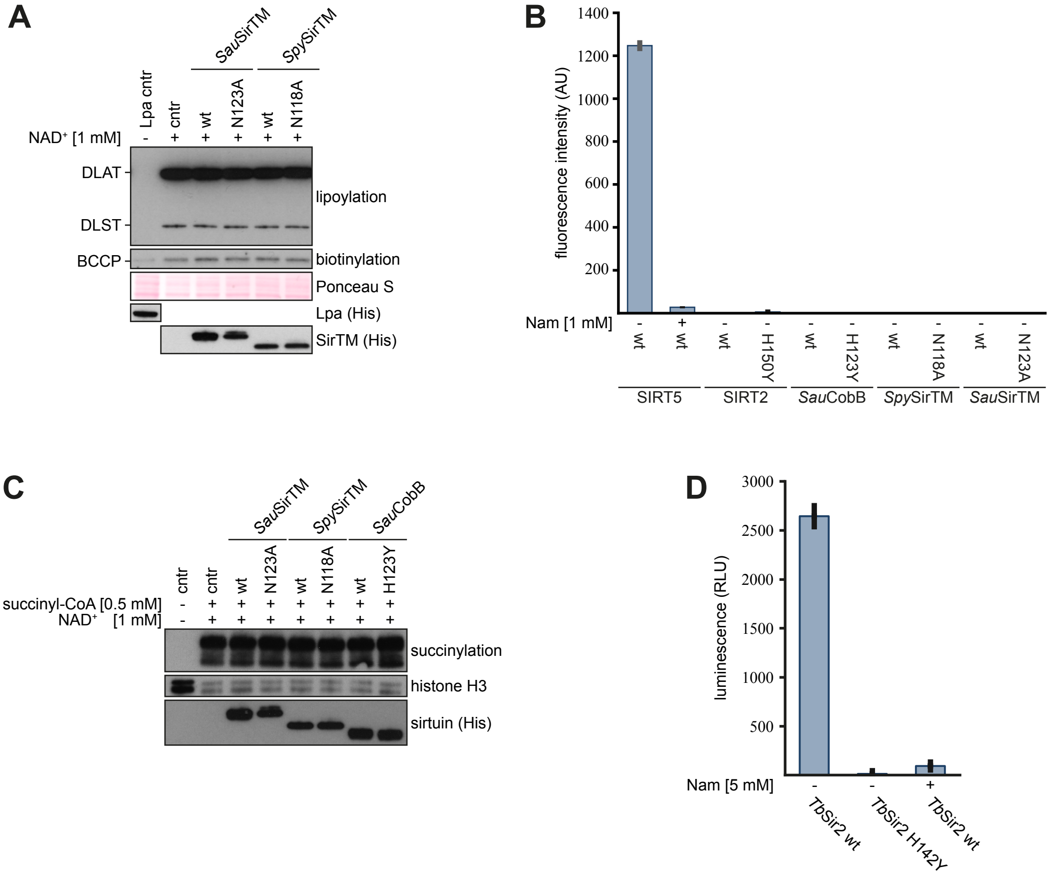
A
SauSirTM
SpySirTM
Lpa cntr
cntr
wt
N123A
wt
N118A
NAD⁺ [1 mM]
- + + + + +
DLAT
DLST
lipoylation
BCCP
biotinylation
Ponceau S
Lpa (His)
SirTM (His)
B
1400
1200
1000
800
600
400
200
fluorescence intensity (AU)
Nam [1 mM]
- + - - - - - - - -
wt
wt
wt
H150Y
wt
H123Y
wt
N118A
wt
N123A
SIRT5
SIRT2
SauCobB
SpySirTM
SauSirTM
C
SauSirTM
SpySirTM
SauCobB
cntr
cntr
wt
N123A
wt
N118A
wt
H123Y
succinyl-CoA [0.5 mM]
- + + + + + + +
NAD⁺ [1 mM]
- + + + + + + +
succinylation
histone H3
sirtuin (His)
D
3000
2500
2000
1500
1000
500
luminescence (RLU)
Nam [5 mM]
- - +
TbSir2 wt
TbSir2 H142Y
TbSir2 wt

Figure S3

Figure S4. The specific ADP-ribosylation of GcvH-L (related to Figure 4).

(A) ADP-ribosylation of GcvH-L can be reversed by treatment with the human phosphodiesterase NUDT16.

(B) MARylation assay performed in the presence of free lipoic acid or lipoamide. Control reactions were carried out in the absence of substrate as well as in the presence of lipoic acid and LplA2.

(C) MARylation assay performed with *Spy*SirTM and unmodified *Spy*GcvH-L in the presence of unmodified, lipoylated and acetylated peptides with sequences derived from *Spy*GcvH-L and histone H3. Control reaction was carried out in the presence of lipoylated *Spy*GcvH-L and *Spy*LplA2.

(D) MARylation of the peptides utilized in (C) was assessed by TLC. No retention of radiolabel at the origin was observed, which would be expected in case the peptide substrate incorporated the ADPr (compare also Figure 4G). The enzyme present in the assay is indicated on top and the peptide underneath the panel.

(E) MARylation assay performed with unmodified and biotinylated BCCP.

(F) *S. aureus* SirTM and Macro were tested for NAD^+ hydrolase activity by incubation of ^{32}P-NAD^+ with the enzymes under standard MARylation assay conditions (compare Materials and Methods). Human MacroD1 was used as unrelated control. Samples were resolved by TLC and analyzed by autoradiography.

(G) Inhibition susceptibility assay was carried out using the general sirtuin inhibitors nicotinamide and Tenovin-6. *In vivo* lipoylated *Sau*GcvH-L, *Sau*SirTM and inhibitor in indicated concentration were incubated for 10 min prior to the addition of NAD^+. Control reactions were carried out in the absence of the inhibitor (cntr).

(H) *In vivo* lipoylated *Sau*GcvH-L was MARylated using *Sau*SirTM. 1 µM of labeled *Sau*GcvH-L was incubated with increasing amounts (0.001, 0.01, 0.05, 0.25, 1, 25 and 10 µM) of either *Sau*Macro (upper panel) or human MacroD1 (lower panel). Control reactions were carried out in the absence of a macrodomain protein (cntr).

(I) The GcvH-L lipoylation-dependence of the operonal macrodomain was tested by removing the lipoylation from lipooylated and MARylated GcvH-L via Lpa prior to carrying out the de-MARylation reaction with Macro, MacroD1 or YmdB. Reactions not treated with Lpa in the absence or presence of Macro were used as controls.

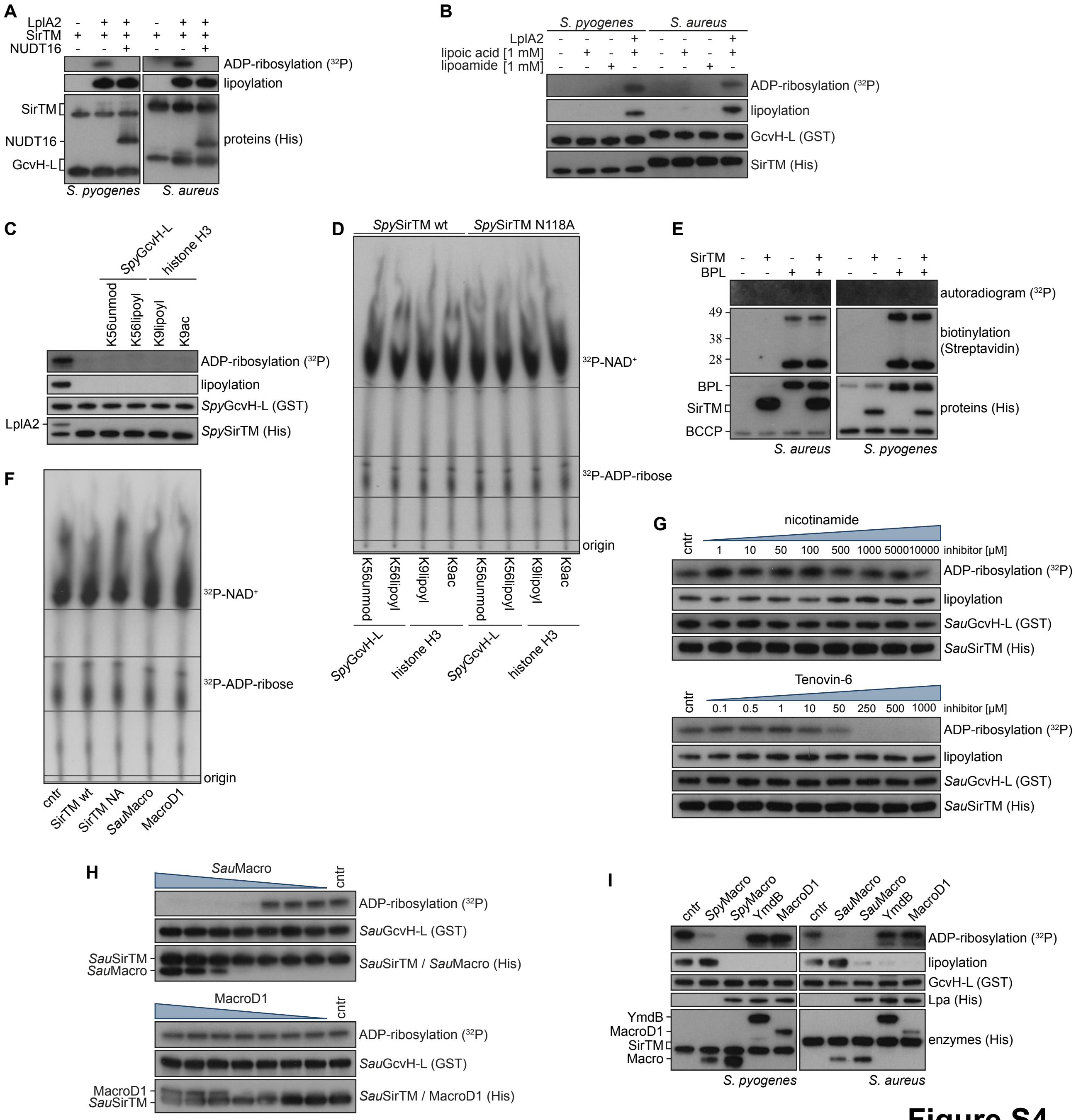
A
LplA2 - + + - + +
SirTM + + + + + +
NUDT16 - - + - - +
ADP-ribosylation (32P)
lipoylation
SirTM
NUDT16
GcvH-L
proteins (His)
S. pyogenes
S. aureus
B
S. pyogenes
S. aureus
LplA2
lipoic acid [1 mM]
lipoamide [1 mM]
ADP-ribosylation (32P)
lipoylation
GcvH-L (GST)
SirTM (His)
C
SpyGcvH-L
histone H3
K56unmod
K56lipoyl
K9lipoyl
K9ac
ADP-ribosylation (32P)
lipoylation
SpyGcvH-L (GST)
LplA2
SpySirTM (His)
D
SpySirTM wt
SpySirTM N118A
32P-NAD+
32P-ADP-ribose
origin
K56unmod
K56lipoyl
K9lipoyl
K9ac
SpyGcvH-L
histone H3
E
SirTM
BPL
autoradiogram (32P)
49
38
28
biotinylation (Streptavidin)
BPL
SirTM
BCCP
proteins (His)
S. aureus
S. pyogenes
F
32P-NAD+
32P-ADP-ribose
origin
cntr
SirTM wt
SirTM NA
SauMacro
MacroD1
G
nicotinamide
cntr
1 10 50 100 500 1000 5000 10000
inhibitor [µM]
ADP-ribosylation (32P)
lipoylation
SauGcvH-L (GST)
SauSirTM (His)
Tenovin-6
cntr
0.1 0.5 1 10 50 250 500 1000
inhibitor [µM]
ADP-ribosylation (32P)
lipoylation
SauGcvH-L (GST)
SauSirTM (His)
H
SauMacro
cntr
ADP-ribosylation (32P)
SauGcvH-L (GST)
SauSirTM
SauMacro
SauSirTM / SauMacro (His)
MacroD1
cntr
ADP-ribosylation (32P)
SauGcvH-L (GST)
MacroD1
SauSirTM
SauSirTM / MacroD1 (His)
I
cntr
SpyMacro
SpyMacro
YmdB
MacroD1
cntr
SauMacro
SauMacro
YmdB
MacroD1
ADP-ribosylation (32P)
lipoylation
GcvH-L (GST)
Lpa (His)
YmdB
MacroD1
SirTM
Macro
enzymes (His)
S. pyogenes
S. aureus

Figure S4

Figure S5. Structural comparison of *Spy*SirTM and *Spy*GcvH-L with close structural homologues (related to Figure 5).

(A) Ribbon representation of a structural overlay of *Spy*SirTM (orange) with yeast HST2 (green, PDB: 1SZC) and *T. maritima Tm*Sir2 (blue, PDB: 2H4F). The structures of *Spy*SirTM and *Tm*Sir2 contain NAD^+ (yellow and dark blue, respectively) and HST2 carba-NAD^+ (dark green).

(B) Comparison of the NAD^+ coordination of *Spy*SirTM and *Tm*Sir2. Side chain of residues involved in the coordination or catalysis are indicated as sticks. The coloration is as in (A).

(C) De-MARylation assays performed with the operon encoded macrodomains using human MacroD1 as control and MARylated human PARP1 E988Q and PARP3 as substrates.

(D) Ribbon representation of a structural overlay of *Spy*GcvH-L (black) with canonical GcvHs from bovine (*Bta*GcvH, orange, PDB: 3KLR), pea (*Psa*GcvH, green, PDB: 1DMX) and *M. tuberculosis* (*Mtu*GcvH, yellow, PDB: 3HGB). The side chains of the lipoyl attachment site (K56) and residues involved in ADP-ribosylation (E24 and D27) are indicated in the left panel, whereas the right panel shows the displacement of the β4/β5 loop (arrow heads) due to the presence of Arg72 in GcvH-L.

(E) Comparison of residues involved in tethering the C-terminal α-helix, which is absent in GcvH-L, to the core structure. The coloration is as in (D) and helix α1, which contains the residues involved in ADP-ribose attachment (GcvH-L, highlighted), is shown in dark blue. Electrostatic interaction are given as dotted lines. Note, the α-helix/core structure interaction minimally consists of a structural motif composed of four conserved residues ([YF], H, [ST] and Y) that is extended by non-conserved interactions in the cases of the bovine and pea GcvHs. Residue numbers correspond to endogenous full-length proteins as shown in the alignment in Figure S6B.

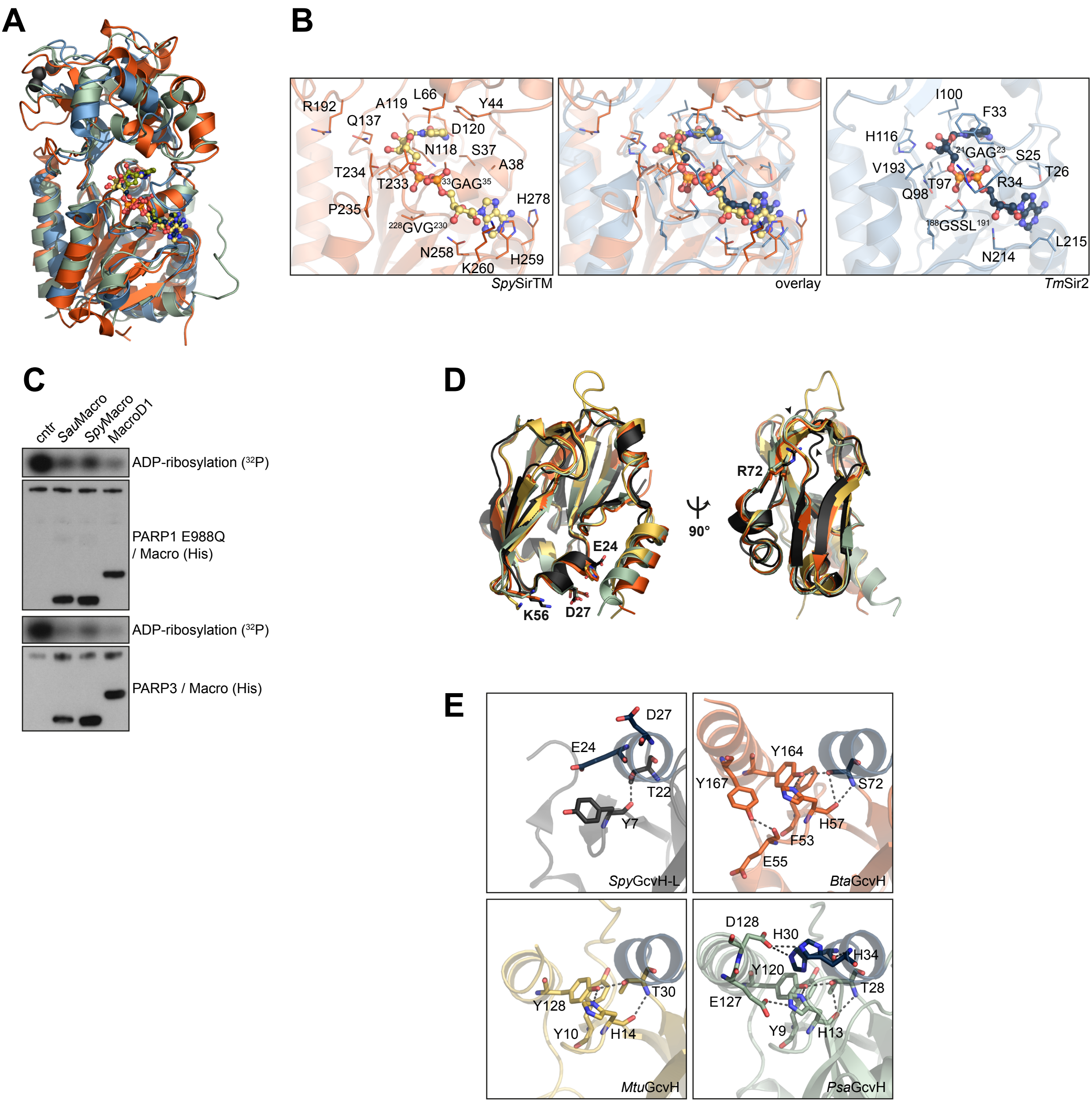
A
B
R192
A119
L66
Y44
Q137
D120
N118
S37
A38
T234
T233
33GAG35
H278
P235
228GVG230
N258
H259
K260
SpySirTM
overlay
I100
F33
H116
21GAG23
S25
V193
T26
T97
R34
Q98
188GSSL191
L215
N214
TmSir2
C
cntr
SauMacro
SpyMacro
MacroD1
ADP-ribosylation (32P)
PARP1 E988Q
/ Macro (His)
ADP-ribosylation (32P)
PARP3 / Macro (His)
D
R72
E24
90°
K56
D27
E
D27
E24
T22
Y7
SpyGcvH-L
Y164
Y167
S72
H57
F53
E55
BtaGcvH
T30
Y128
Y10
H14
MtuGcvH
D128
H30
H34
Y120
T28
E127
Y9
H13
PsaGcvH

Figure S5

Figure S6. Comparison of GcvH-Ls amongst each other and in comparison to canonical GcvHs (related to Figure 5).

(A) Multiple sequence alignment of representative GcvH-L proteins. The secondary structure elements of *Spy*GcvH-L are indicated on top of the alignment and the lipoyl attachment site underneath (yellow diamond).

(B) Multiple sequence alignment comparing *Sau*GcvH-L and *Spy*GcvH-L with canonical GcvH proteins (including bacterial, archaeal, fungal, plant and animal representatives). Secondary structure elements of *Spy*GcvH-L (orange) and *Mtu*GcvH (blue) are indicated on top of the alignment and the lipoyl attachment side underneath (yellow diamond). Note, sequences from higher organism contain an N-terminal mitochondrial targeting sequence (*Psa*-, *Cga*-, *Foc1*- and *Bta*GcvH). For sequence accession numbers see Table S2.

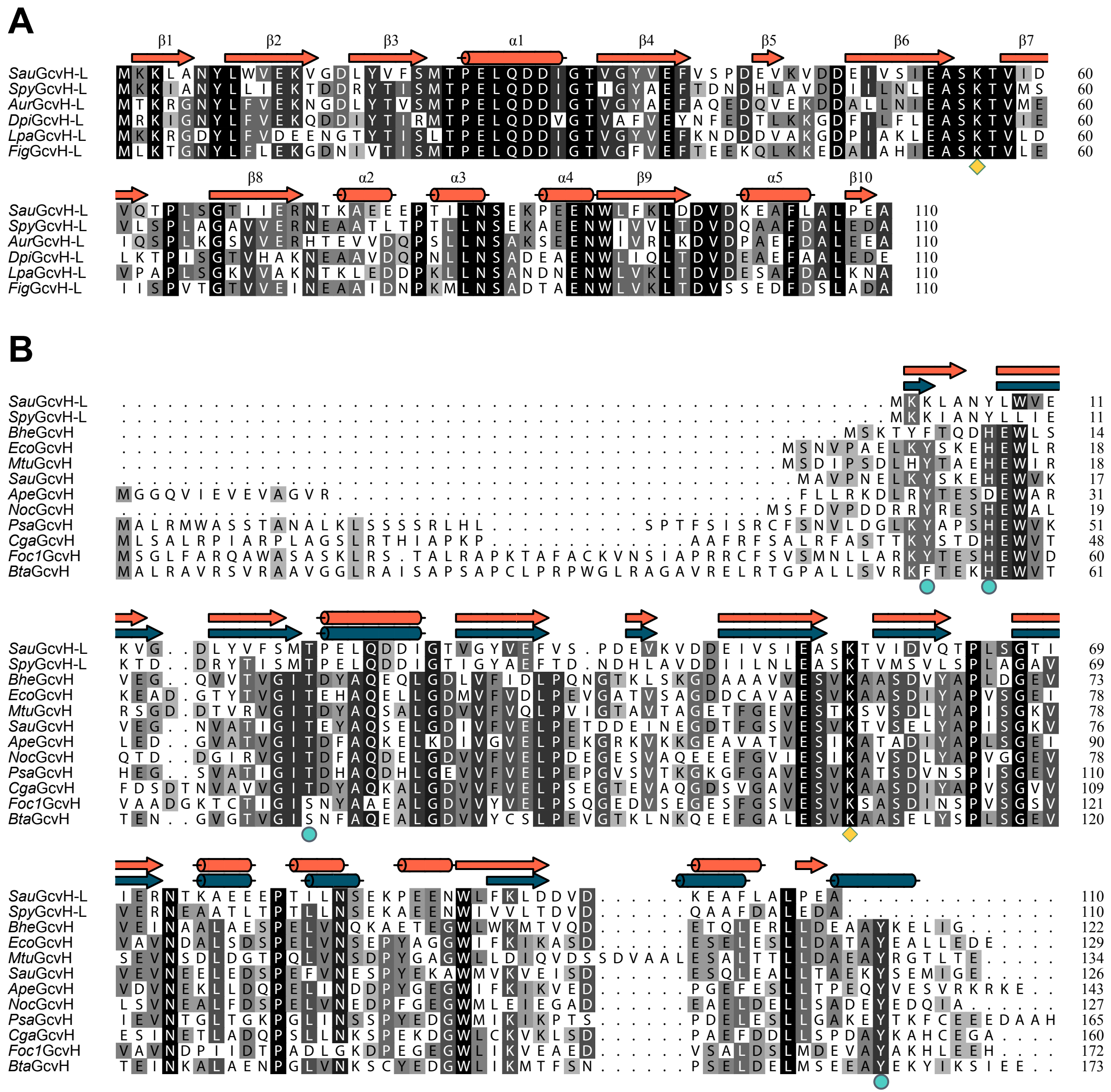
A
β1
β2
β3
α1
β4
β5
β6
β7
β8
α2
α3
α4
β9
α5
β10
SauGcvH-L
SpyGcvH-L
AurGcvH-L
DpiGcvH-L
LpaGcvH-L
FigGcvH-L
B
SauGcvH-L
SpyGcvH-L
BheGcvH
EcoGcvH
MtuGcvH
SauGcvH
ApeGcvH
NocGcvH
PsaGcvH
CgaGcvH
Foc1GcvH
BtaGcvH

Figure S6

Figure S7. *C. albicans mfs1Δ* mutant construction and confirmation (related to Figure 6).

(A) Schematic representation of the genomic locus of wild type *C. albicans* or *mfs1Δ* (*orf19.2285*) mutant clones. The position of the Southern blot probe and restriction sites used for clone verification are shown.

(B) Genomic DNA from wild type *C. albicans* or *mfs1Δ* mutant clones was isolated and digested with ClaI and NcoI (see (A) for positions). The Southern blot assay was performed using the NEBlot Phototope Kit and the Phototope-Star Detection Kit, as per manufacturer's instructions.

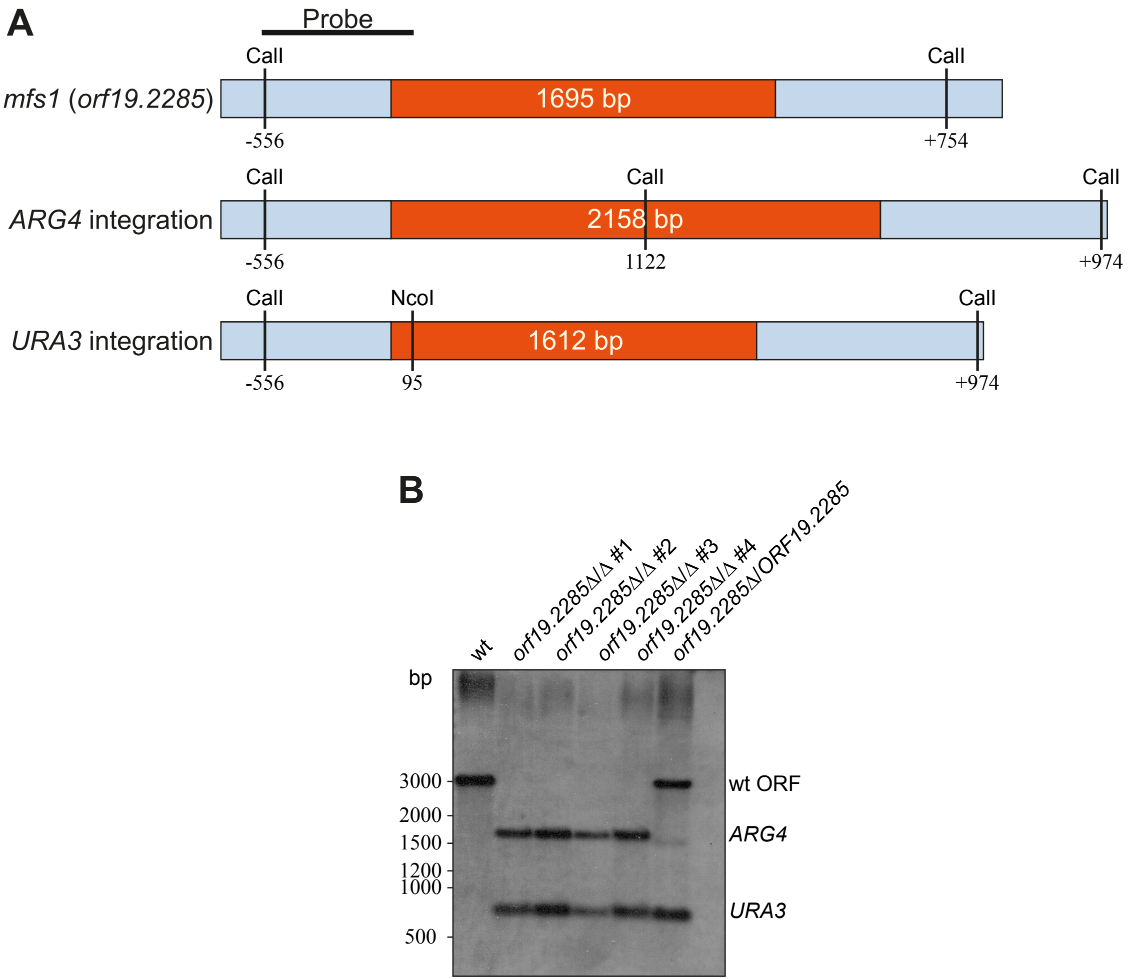
A
Probe
Call
Call
mfs1 (orf19.2285)
1695 bp
-556
+754
Call
Call
Call
ARG4 integration
2158 bp
-556
1122
+974
Call
NcoI
Call
URA3 integration
1612 bp
-556
95
+974
B
wt
orf19.2285Δ/Δ #1
orf19.2285Δ/Δ #2
orf19.2285Δ/Δ #3
orf19.2285Δ/Δ #4
orf19.2285Δ/ORF19.2285
bp
3000
2000
1500
1200
1000
500
wt ORF
ARG4
URA3

Figure S7

Supplemental Tables

Table S1. Proposed interactions for class M defining residues of *Spy*SirTM (related to Figure 1 and 5).

Motif	Residues in *Spy*SirTM	Role
Cx(3)C[a]	Cys145 – Cys149	Part of Zn^{2+} coordinating motif present in the small variant domain.
GVGx[NT]TP	Gl229 – Pro235	The GVGx[NT]TP motif is located on the loop connecting the Rossmann fold β6 sheet with α14 helix and forms H-bonds between the backbone nitrogen and α- and β-phosphate groups of the NAD^+ substrate.
GAGxSAx(2)Gx(2)Y	Gly33 – Tyr44	Part of the GAGxSAx(2)Gx(2)Y motif are located on the loop connecting β1 sheet with α2 helix. The side chains form both hydrogen bonds with the pyrophosphates and VdW interactions with the nicotinamide moiety of NAD^+.
T[ST]N[VA]D	Thr116-Asp120	Present on the loop between the β2 strand and the α8 helix. Forms VdW interactions with the nicotinamide ribose moiety and water mediated H-bond with the β-phosphate group.
QG	Gln137 + Gly138	The glutamine is involved in the catalytic mechanism.
R	Arg192	Presumed catalytic. Forms hydrogen bonds with the Gln137 residue either polarizing it for catalysis or involved in target recognition.
NH	Asn258 + His259	Present on the loop connecting the Rossmann fold β7 and β8. Asn258 forms H-bonds with the 3'-OH and 2'-OH groups of the adenine ribose and the His259 imidazole ring forms stacking interactions against the adenine moiety upon binding of NAD^+.

(a) The spacing between the first two cysteine residues coordinating the zinc ion is specific for class M sirtuins. All other classes contain a Cx(2)C motif.

Table S2. Accession numbers of sequences used in the generation of multiple sequence alignments (related to Figure S1 and S6).

**Abbreviation**	**Organism**	**accession**[a]
Comparison of SirTMs		
*Cal*Mfs1	*Candida albicans*	238883620
*Ate*Mfs1	*Aspergillus terreus*	115388759
*Foc1*Mfs1	*Fusarium oxysporum f. sp. cubense* RACE 1	477523926
*Aur*SirTM	*Aerococcus urinae*	326651405
*Spy*SirTM	*Streptococcus pyogenes*	14246094
*Sau*SirTM	*Staphylococcus aureus*	134271959
*Cbo*SirTM	*Clostridium bolteae*	524018386
*Ssp*SirTM	*Selenomonas sputigena*	330839837
Comparison of sirtuin classes		
*Sau*SirTM	*Staphylococcus aureus*	134271959
*Spy*SirTM	*Streptococcus pyogenes*	14246094
*Sau*CobB	*Staphylococcus aureus*	88196109
*Tb*Sir2	*Trypanosoma brucei*	74830204
*Tm*Sir2	*Thermotoga maritima*	38257895
HST2	*Saccharomyces cerevisiae*	1708326
*Atr*Sir2L1	*Amborella trichopoda*	586696836
SIRT1	*Homo sapiens*	7657575
SIRT4	*Homo sapiens*	38258657
SIRT5	*Homo sapiens*	38258652
SIRT7	*Homo sapiens*	38258650
Comparison of GcvH-Ls		
*Sau*GcvH-L	*Staphylococcus aureus*	14246092
*Spy*GcvH-L	*Streptococcus aureus*	134271957
*Aur*GcvH-L	*Aerococcus urinae*	326650593
*Dpi*GcvH-L	*Dolosigranulum pigrum*	374563578
*Lpa*GcvH-l	*Lactobacillus parafarraginis*	363716801
*Fig*GcvH-L	*Facklamia ignava*	405579715
Comparison of GcvH-Ls and canonical GcvHs		
*Sau*GcvH-L	*Staphylococcus aureus*	14246092
*Spy*GcvH-L	*Streptococcus pyogenes*	134271957
*Bhe*GcvH	*Bartonella henselae*	654326147
*Eco*GcvH	*Escherichia coli*	253978883
*Mtu*GcvH	*Mycobacterium tuberculosis*	395138596
*Sau*GcvH	*Staphylococcus aureus*	14246602
*Ape*GcvH	*Aeropyrum pernix*	499164536
*Noc*GcvH	*Natronococcus occultus*	505134954
*Psa*GcvH	*Pisum sativum*	121080
*Cga*GcvH	*Cryptococcus gattii*	757372505
*Foc1*GcvH	*Fusarium oxysporum f. sp. cubense* RACE 1	477512885
*Bta*GcvH	*Bos taurus*	27807309

(a) GenBank accession numbers

Supplemental Experimental Procedures

Sample analysis and antibodies

Reactions for analysis were stopped by adding LDS sample buffer and incubation for 5 min at 90°C. Subsequently the samples were resolved by SDS-PAGE and either vacuum dried for autoradiography or transferred onto a PVDF membrane. Immunoblot analyses were carried out using primary and secondary antibodies as indicated. Proteins were detected by enhanced chemiluminescence (Pierce).

Antibodies used in this study: Mouse monoclonal hexahistindine antibody (Clonetech), rabbit polyclonal lipoic acid antibody (Calbiochem), rabbit polyclonal histone H3 antibody (Millipore), rabbit acetylated lysine antibody (Cell Signaling Technology) and rabbit polyclonal succinyllysine antibody (PTM Biolabs), horseradish peroxidase-conjugated goat polyclonal GST antibody (Abcam), streptavidin (Pierce), secondary swine anti-rabbit (Dako) and secondary goat anti-mouse antibodies (Dako).

Plasmid construction

The construction of the human MacroD1, PARG, PARP1 E988Q and SIRT2, *E. coli* YmdB, *E. faecalis* Lpa and *T. brucei Tb*Sir2 expression vectors were described previously (Barkauskaite et al., 2013, Chen et al., 2011, Fahie et al., 2009, Rack et al., 2014, Slade et al., 2011, Spalding and Prigge, 2009). The coding sequences of all constructs from *S. aureus* and *S. pyogenes* were amplified from genomic DNA (*S. aureus*, ATCC 700699D-5; *S. pyogenes*, kind gift of Christoph Tang). The pET9H_3 expression vector was constructed by adaptor based cloning of 5' – GGATCCACGC GTTAACCGGT ACCAACCAAC CATGGGTCCC TGTGGTAACA CTTCCAGGCC ACTACCGTGA TGATGGTGGT GATGGTGCAT into the parental pET9d (Novagen) vector via the NcoI/BamHI restriction sides and thereby introducing a hepta-histidine tag, a HRV3C cleavage site and a small multiple cloning site into the vector. The underlined sequences mark the restored BamHI and altered NcoI side respectively. For GST-affinity purification the coding sequences of *Eco*GcvH, *Sau*GcvH and -L, *Spy*GcvH-L, *Sau*SirTM and *Sau*LplA2 were transferred into pDEST15 and the sequences of *Sau*OYE, *Sau*LLM and *Sau*Macro into pDEST24 via gateway cloning (Life Technology) using pDONR221 as donor vector. GST was expressed from the empty pGEX-4T1

vector (GE Healthcare). For nickel-affinity purification the coding sequences of *Eco*LplA, *Sau*LplA1 and 2, *Spy*LplA2 and *Spy*OYE were transferred into pDEST17, and the sequence of *Sau*CobB was transferred into pDEST42 via gateway cloning. The coding sequences of human SIRT4ΔMTS (removing residues 1 – 28 which encode the MTS (Haigis et al., 2006) and adding a C-terminal GSG linker and hepta-histidine tag) was introduced into pET9d, the sequences of *Sau*BCCP and *Spy*BCCP were introduced into pET9H_3 (adding a N-terminal double-tryptophan for UV quantitation), the sequences *Sau*GcvH-L, *Spy*GcvH-L and *Spy*LLM were introduced into pET21a and the sequences of *Sau*Macro, SpyMacro, *Sau*SirTM and *Spy*SirTM were introduced into pET28 via restriction based cloning. The coding sequences of *Sau*BPL and *Spy*BPL were introduced into pNH-TrxT (Savitsky et al., 2010) via ligation independent cloning.

All indicated mutations were introduced via PCR based site-directed mutagenesis.

***In silico* analysis of the sirtuin domain of SirTMs and GcvH-L**

Selection of representative sequences of formerly described sirtuin classes is based on the work of Greiss and Gartner (Greiss and Gartner, 2009). SirTM single domain sirtuins from bacterial operon are *Clostridium bolteae* (*Cbo*SirTM, gi|524018386), *Selenomonas sputigena* (*Ssp*SirTM, gi|330839837) and *Veillonella parvula* (*Vpa*SirTM, gi|269094432) and from the extended operon *Lactobacillus parafarraginis* (*Lpa*SirTM, gi|363716803), *Staphylococcus aureus* (*Sau*SirTM, gi|57634611) and *Streptococcus pyogenes* (*Spy*SirTM, gi|13622344). Sirtuin domain sequences from fungal fusion proteins *Candida albicans* (*Cal*Mfs1, gi|238883620), *Entamoeba dispar* (*Edi*Mfs1, gi|167540052) and *Fusarium oxysporum f. sp. cubense* RACE1 (*Foc1*Mfs1, gi|477523926) were extracted based on Clustal Omega alignment (Sievers et al., 2011) with bacterial single domain sirtuins. Sequences for tree construction were aligned using JalView 2.8.0b1 (Waterhouse et al., 2009) and the Clustal Omega program implemented therein. An unrooted phylogram was generated by neighbor-joining method using SplitTrees4 V4.13.1 (Huson and Bryant, 2006). Confidence level of the class clades was estimated to be ≥ 98.8% by bootstrapping using 10000 replicates.

Multiple sequence alignments of GcvH(-L) proteins and sirtuins were generated using JalView 2.8.0b1 and the MAFFT L-INS-I algorithm implemented therein (Katoh et al., 2005). Comparative

sirtuin domain alignment was generate by initially aligning full-length protein using MAFFT L-INS-I and extracting SirTM homology regions. The extracted sequences were re-aligned using Clustal Omega. Alignment representations were created with ALINE (Bond and Schuttelkopf, 2009). Sequences details and accession numbers are given in Table S2.

Bacteria strains and culture

For generation of *E. coli* cell extracts BL21(DE3)Δ*CobB* cells (Castano-Cerezo et al., 2015) were grown on LB agar plates supplemented with kanamycin (30 µg/ml) at 37°C. A single colony was pick and transferred into LB medium containing kanamycin. Cells were grown at 37°C and 200 rpm to an OD_{600} 0.6 and harvested by centrifugation at 4°C. The pallet was washed twice with extraction buffer [50 mM TrisHCl (pH 8), 200 mM NaCl, 1 mM DTT and 10% (v/v) glycerol] and finally resuspended in 1 mL extraction buffer. Cells were lysed by 6 cycles of sonication and insoluble cell debris removed by centrifugation at 20000 xg for 30 min at 4°C.

For generation of *S. aureus* cell extracts SH1000 cells were grown on BHI agar plates. BHI medium was inoculated with a single colony and grown overnight at 37°C and 150 rpm. The overnight culture was diluted 1:100 in fresh BHI medium and culture was allowed to grow for 2 hr. Cells were harvested by centrifugation and washed twice in extraction buffer before resuspention in 1 mL extraction buffer. Partial lysis was achieved by incubation with BugBuster protein extraction reagent (Novagen) and lysozyme for 2 hr at RT on a rotating wheel. Unlysed cell and cellular debris was removed by centrifugation at 20000 xg for 30min at 4°C.

***Candida* strains and culture**

The opening reading frame encoding amino acids 1-492 of the *Candida albicans* macrodomain-fused SirTM (*mfs1*), *orf19.2285* (C2_07280W), was deleted in the BWP17 strain background (*ura3*::imm434/*ura3*::imm434 *iro1/iro1*::imm434 *his1*::hisG/*his1*::hisG *arg4/arg4*). Selection markers *ARG4* and *URA3* were used to create a homozygous deletion mutant by homologous recombination. The marker-matched wild type strain (DAY286) was used as the control in all experiments. Confirmation of homozygous deletion by Southern blot analysis is shown in Figure S7. Strains were

maintained in 15% glycerol (v/v) at -80°C. Working stocks were streaked onto YPDUri agar plates (1% yeast extract, 2% peptone, 2% glucose, 2% agar, 80 µg/ml uridine), strains grown at 30°C for 2-3 days, and then plates maintained at room temperature. For experiments testing the sensitivity to hydrogen peroxide (H_2O_2), strains were inoculated into YPDUri media over night at 30°C, 200 rpm and grown to saturation. Cultures were then diluted to OD_{600} 0.5 and 2.5 µl dropped onto YPDUri control or H_2O_2-containing plates, followed by incubation at 30°C for 3 days. Alternatively, broth H_2O_2 susceptibility assays were performed in a 96-well microplate, following a method described by (Park et al., 2008), with modification. Overnight *C. albicans* cultures were harvested by centrifugation (5 min, 2500 xg), and re-suspended in YPD medium to a density of $1 \cdot 10^3$ cells/ml. One hundred microliters of fungal suspension were placed into a well of a 96-well microplate and mixed with H_2O_2 to reach concentrations of 0 mM, 5 mM, 5.5 mM, 6 mM, and 6.5 mM respectively. The microplates were incubated statically at 37°C for 24 hr, and fungal growth read with the Infinite 200 PRO Tecan spectrometer at OD_{600}. Percentage of fungal growth was calculated relative to no H_2O_2 controls.

Human 293T cell culture

293T cells were cultivated in high glucose DMEM (Dulbecco's modified Eagle's medium) supplemented with 10% FBS, 2 mM and L-glutamine. Cells were incubated at 37°C in humidified atmosphere with 5% CO_2. For extract preparation cells were grown on a 100 mm culture dish to confluence and washed twice with PBS. Cells were scraped off in 1 mL extraction buffer and lysed by 3 cycles of sonication. Insoluble cell debris was removed by centrifugation at 20000 xg for 30 min at 4°C.

Protein expression and purification

Recombinant proteins were expressed in Rosetta (DE3) cells grown in LB medium supplemented with 2 mM $MgSO_4$ and appropriate antibiotics at 37°C to OD_{600} 0.6. In addition, expression cultures of lipoamidase were supplemented with 2% glucose (w/v), 5 mM potassium acetate and 5 mM sodium succinate. Expression was induced with 0.4 mM IPTG and 5 µM zinc acetate in case of zinc-containing enzymes. Cells were grown at 30°C and harvested 4 hr post-induction by centrifugation.

Lipoamidase expression was carried out at 18°C overnight. SeMET *Spy*SirTM protein was produced as above but using minimal medium and SeMET growth medium (SelenoMet Medium Base plus SelenoMet Nutrient Mix, Molecular Dimensions) supplemented with 50 mg/mL of SeMET after 40 min of inoculation. The SeMET SpySirTM protein was expressed at 30 °C for 18 hr.

Recombinant His-tagged proteins were purified by Ni^{2+}-NTA chromatography (Qiagen) according to the manufacturer's protocol using the following buffers: all buffer contained 50 mM TrisHCl (pH 8) and 500 mM NaCl; additionally, the lysis/binding buffer contained 10 mM imidazole, the washing buffer contained 30 mM imidazole, and the elution buffer contained 500 mM imidazole.

Recombinant GST-tagged proteins were purified using Glutathione Sepharose 4B (GE Life Sciences) according to the manufacturer’s protocol using the following buffers: lysis/binding/washing PBS containing 1 mM DTT and elution 50 mM TrisHCl (pH 8), 200 mM NaCl containing 10 mM reduced glutathione and 1 mM DTT.

All proteins were dialyzed over night against 50 mM TrisHCl (pH 8), 200 mM NaCl, 1 mM DTT.

Proteins intended for crystallization underwent affinity purification over a HisTrap HP column (GE Healthcare) followed by size exclusion chromatography using a HiLoad Superdex 75 column (GE Healthcare). Proteins were concentrated using Vivaspin 20 columns (GE Healthcare).

Lipoylation assays

Lipoylation reactions were carried out in lipoylation buffer [50 mM TrisHCl (pH 8), 200 mM NaCl, 5 mM ATP, 2.4 mM lipoic acid, 1 mM $MgCl_2$, 1 mM DTT] using 1 μM LplA and 2 μM target protein or in a 1:2 molar ratio if higher concentrations were required for subsequent reactions. Reactions were incubated for 30 min at 30°C before analysis by immunoblot or further processing.

Substrate specificity of *Sau*LplA2 was assessed by incubation of 1 µM of *Sau*GcvH-L with increasing concentrations of lipoic acid or lipoamide (0.24, 2.4, 24 or 240 µM) in the presence of 1 µM enzyme at 37°C for 30 min. Control reactions were carried out at highest substrate concentration in the absence of *Sau*LplA2. All samples were analyzed by immunoblot.

For *in vivo* lipoylation of GcvH-L, Rosetta (DE3) cells were grown as described above. Upon induction cultures were supplemented with 100 μM lipoic acid and grown for 3 hr at 30°C. Further

protein synthesis was inhibited by addition of 150 µg/ml kanamycin and cells were incubated for an additional 60 min. Control reactions were supplemented with solvent instead of lipoic acid. Samples were taken before induction and kanamycin addition and at the end of the incubation period to evaluate the success of *in vivo* lipoylation. All samples were analyzed by immunoblot.

Biotinylation assay

BCCP biotinylation was carried out in biotinylation buffer [50 mM TrisHCl (pH 8), 200 mM NaCl, 1 mM DTT, 1 mM MgCl2, 300 µM biotin and 5 mM ATP] at 30°C for 1 hr using 2 µM BCCP and 1 µM BPL. Reactions were stopped by twice purifying the protein over PD SpinTrap G25 (Ge Healthcare) columns. Purified proteins were used as substrates for debiotinylation or ADP-ribosylation reaction (see below).

Deacylation assays

Delipoylation of *in vitro* and *in vivo* lipoylated GcvH-L was carried out in deacylation buffer [50 mM TrisHCl (pH 8), 200 mM NaCl, 10 mM $MgCl_2$, 1 mM DTT, 1 mM NAD^+] using 1 µM recombinant sirtuin and 1 µM GcvH-L. Reactions were incubated at 30°C for 2 hr.

Debiotinylation was carried out analogously using purified, biotinylated BCCP as substrate (see above). All reactions were stopped by addition of LDS sample buffer.

Deacylation of bacterial and human cell extracts (40 µg) was carried out in deacylation buffer for 2 hr at 30°C. Reactions were supplemented with 2 µM recombinant sirtuin as indicated. Lpa control reaction were carried out in PBS using 1 µg crude Lpa purification. All reactions were stopped by addition of LDS sample buffer.

Delipoylation using Lpa was carried out by mixing the initial lipoylation reaction or *in vivo* lipoylated GcvH-L in lipoylation buffer (1/3 final vol.) with 10x PBS (1/10 final vol.) and 1µg crude Lpa purification. The volume was adjusted with dH_2O and the reaction incubated for 2 hr at 30°C. All reactions were stopped by addition of LDS sample buffer.

Deacetylation of synthetic penta-acetylated histone H3 (Active Motif) was carried out as described earlier (Rack et al., 2014). Briefly, deacetylation was carried out in deacylation buffer [50

mMTrisHCl (pH 8), 137 mM NaCl, 2.7 mM KCl and 10 mM $MgCl_2$, 0.5 mM DTT] using 2 µM purified sirtuin, 1 mM NAD^+ and 1 µM penta-acetylated histone H3. Reactions were incubated at 30°C for 2 hr and stopped by addition of LDS sample buffer.

Desuccinylation of a fluorogenic succinyl-peptide was tested with the Fluorogenic SIRT5 assay kit (BPS bioscience) according to the manufacturer's recommendations using 300 ng sirtuin per reaction.

For desuccinylation Xenopus-derived histone octamers were non-enzymatically succinylated as described earlier (Wagner and Payne, 2013). Briefly, succinylation reaction were carried out in succinylation buffer [50 mM TrisHCl (pH 8 at 37°C), 150 mM NaCl, 0.5 mM succinyl-CoA] using 3 µM histone octmaers as substrate. Reactions were incubated for 6 hr at 37°C at 400 rpm in an Eppendorf Thermomixer and subsequently stored on ice until further use. Desuccinylation was carried out as described for histone deacetylation using 1 µM succinylated histone octamers as substrate.

ADP-ribosylation assays

ADP-ribosylation reactions were carried out in MARylation buffer [50 mM TrisHCl (pH 8), 200 mM NaCl, 1 mM $MgCl_2$, 1 mM DTT] using 1 µM sirtuin, 1 µM target protein, 2 µCi $^{32}P\text{-}NAD^+$ and 5 µM unlabeled NAD^+. A 1:1 molar ratio was used if higher concentrations were required for subsequent reactions. Reactions were incubated for 60 min at 30°C before analysis by autoradiography and immunoblot or further processing. For kinetic studies the incubation time was varied as indicated.

ADP-ribosylation reactions using purified, biotinylated BCCP (see above) as substrate were carried out analogously.

For inhibitor studies 6 mM Tenovin-6 (Axon Medchem) stock solution was prepared in 18% DMSO (v/v) and 50 mM HCl. Further dilutions were prepared with 18% DMSO. Final DMSO concentration in the assays was 3%. Nicotinamide (Sigma) solutions were prepared in dH_2O. All reaction components (including inhibitors) were mix and incubated for 10 min at RT before starting the reaction by addition of 2 µCi $^{32}P\text{-}NAD^+$ and 10 µM unlabeled NAD^+.

For reaction containing free lipoic acid or lipoamide both compounds were dissolved in DMSO and diluted to 2% DMSO (v/v) in the final assay conditions. For reaction containing peptides (custom

synthesized by Alta Biosciences (now Abington Health); sequences are ±5 residues of the indicated modification site and two C-terminal tryptophan residues were added for UV quantitation, the N-termini are acetylated and the C-termini amide modified) peptides were dissolved in DMSO and diluted to 5 µM and 2% DMSO (v/v) in final assay conditions.

For ADP-ribosylation comparison reaction were carried out in MARylation buffer using 5 µg target protein (lipoylated *Spy*GcvH-L, glutamate dehydrogenase (Sigma) and histone H1 (New England Biolabs) respectively), 1.5 µM sirtuin, 20 µM unlabeled NAD^+ and 4 µCi ^{32}P-NAD^+. Reaction were carried out at 37°C for 2 hr. Reactions were stopped with LDS sample buffer and analyzed by autoradiography and immunoblot.

De-ADP-ribosylation assays

De-ADP-ribosylation was carried out in [50 mM TrisHCL (pH 8), 200 mM NaCl, 1 mM $MgCl_2$, 1mM DTT] out using 1 µM radiolabelled GcvH-L and 1 µM macrodomain protein. The reactions were incubated for 1 hr at 30°C.

For the concentration gradient experiment using *Sau*Macro and human MacroD1 reactions containing the desired final concentration of macrodomain protein were prepared and started by addition of the initial ADP-ribosylation reaction to achieve a final concentration of 1 µM *Sau*GcvH-L in the reaction.

For the de-MARylation assay using PAR1 EQ and PARP3 self-modification was carried out in PARP buffer [50 mM TrisHCl, 50 mM NaCl, 4 mM $MgCl_2$ and 0.2 mM DTT] using 1 µM enzyme for 30 min at 30°C. The reaction were terminated by addition of 2 µM olaparib.

For direct visualization of radiolabeled ADP-ribose Macro reaction were carried out as follows: 20 µM *in vivo* lipoylated *Sau*GcvH-L were incubated in MARylation buffer in presence of 2 µM *Sau*SirTM wt or N123A protein and 5 µCi ^{32}P-NAD^+ for 45 min at 30°C followed by addition of 20 µM of unlabeled-NAD^+ and incubation for 15 min at 30°C. The reactions were stopped by applying the samples over a PD SpinTrap G25 (GE Healthcare) column in order to remove unbound radiolabeled NAD^+. Subsequently, the reactions were re-supplemented with the MARylation buffer and incubated with 2 µM of S*au*Macro or human MacroD1 protein at 30°C for 1 hr. As control,

poly(ADP-ribose) was synthesized by the self-modification of PARP1 in a reaction mixture containing 6.4 units of PARP1 (Trevigen), 10 µM unlabeled–NAD^+ (Trevigen), 4.4 µCi ^{32}P-NAD^+, activated DNA (Trevigen), 50 mM TrisHCl (pH 7.5) and 50 mM NaCl at RT. Reactions were stopped after 45 min by the addition of the PARP inhibitor KU-0058948 and were purified twice over PD SpinTrap G25 (GE Healthcare) columns to remove unbound radiolabel. Subsequently, the reactions were re-supplemented with 50 mM Tris (pH 7.5) and 50 mM NaCl and 1 µM PARG were added to the reaction. The samples were incubated for 45 min at RT.

All samples were heat-inactivated by incubating the reactions at 80°C for 5 min, spotted onto polyethyleneimine (PEI)-cellulose plates (Macherey-Nagel, Polygram CEL 300 PEI/UV_{254}) and developed in 0.2 M LiCl and 0.2 M formic acid. Dried plates were exposed on X-ray film.

NUDT16 phosphordiesterase assay

To monitor the loss of incorporated radiolabel, ADP-ribosylated GcvH-L (see above) was supplemented with 15 mM $MgCl_2$ and 4 µM NUDT16 and incubated for 3 hr at 30°C. Reactions were stopped with LDS sample buffer and analyzed by autoradiography and immunoblot.

NAD hydrolase activity assay

1 µM *Sau*SirTM wt, *Sau*SirTM N123A, *Sau*Macro or MacroD1 was incubated with 2 µCi ^{32}P-NAD^+, 5 µM unlabeled-NAD^+ in MARylation buffer at 30°C for 1 hr. The reactions were heat-inactivated by incubation at 80°C for 5 min and spotted onto polyethyleneimine (PEI)-cellulose plates (Macherey-Nagel, Polygram CEL 300 PEI/UV_{254}) and developed in 0.2 M LiCl and 0.2 M formic acid. Dried plates were exposed on X-ray film.

GST pull-down assays

Pull-down assays were carried out on 0.2-µm filters (Ultrafree, Millipore), using either 50 µl or 100 µl volumes in all steps. All pull-down assays were carried out at room temperature. Filters were moistened with TZNK/D/T buffer [TZNK buffer (50 mM TrisHCl (pH 8), 150 mM KCl, 12 mM NaCl, 100 µM zinc acetate, 2 mM $MgCl_2$) containing 5 mM DTT and 0.1% (v/v) Triton X-100] before adding glutathione Sepharose 4B. The beads were drained after each step by centrifugation for

30 s. GST-fusion proteins (5 μM) were immobilized on beads by incubation for 30 min on a rotating wheel. Unbound proteins were removed by centrifugation. Beads were washed twice with TZNK/D/T buffer, before introducing 5 μM of the binding partner. Binding reactions were carried out for 30 min on a rotating wheel, before unbound proteins were removed by centrifugation. Beads were washed four times in TZNK/D/T buffer and twice in 50 mM TrisHCl (pH 8), 200 mM NaCl, and 1 mM DTT. Proteins were eluted from the beads by incubation with elution buffer for 30 min on a rotating wheel. Samples were analyzed by immunoblot.

Crystallization

Native and selenomethionine (SeMET) labeled *Spy*SirTM proteins for crystallization were expressed as described above and purified protein concentrated to 14 mg/ml in 25 mM TisHCl (pH 8), 100 mM NaCl and 1 mM DTT.

Initial *Spy*SirTM crystallization conditions were identified using the Morpheus HT-96 matrix screen (Molecular dimensions). Reproducible crystallization was ensured by seeding as follows: crystals were grown at 293 K by sitting-drop vapor diffusion in MRC 96 well plates (Molecular Dimensions) by mixing 120 nL purified protein with 30 nL of seed stock and 150 nL of precipitant solution consisting of 0.1 M amino acids, 0.1 M buffer system 1 (pH 6.5), 30.00% (v/v) EDO_P8K (Molecular Dimensions). Seed stock was prepared using a Seed Bead™ (Hampton Research) with one or more seed crystals and mother liquor solution to 100 µL as the stabilizing solution. Crystals appeared over 48 hr and continued to grow for a further week. For the ADPr and NAD^+ complexes, native crystals were soaked with 1 mM ligand in the mother liquor for 20 hr and 10 min, respectively. The crystals were cryoprotected by dipping them into a solution of 20% ethylene glycol in mother liquor and vitrified by submersion in liquid nitrogen.

Native *Spy*GcvH-L protein for crystallization was expressed as described above and purified protein concentrated to 56 mg/ml in 25 mM TisHCl (pH 8), 100 mM NaCl and 1 mM DTT. A single crystal grew at 293 K by sitting-drop vapor diffusion in a MRC 96 well plate by mixing 100 nL purified protein with 100 nL of precipitant solution consisting of 2.0 M NaH_2PO_4/K_2HPO_4 (pH 7.0). The crystal was cryoprotected in a solution of precipitant plus 15% PEG400.

X-ray Data Collection, Processing, Structure Determination, Refinement, and Analysis

X-ray diffraction data were collected using synchrotron radiation at Diamond Light Source (Rutherford Appleton Laboratory, Harwell, UK) (Table 2) and integrated using iMOSFLM (Battye et al., 2011). Space group identity was checked with POINTLESS (Evans, 2006) before the data sets were scaled with AIMLESS (Evans, 2006). The program autoSHARP (Murshudov et al., 1997) was used for locating the Se atoms in the apo-*Spy*SirTM selenomethionine derivate as well as for refinement and initial phase calculation. *Spy*GcvH-L data was solved via molecular replacement using PHASER (Storoni et al., 2004) with a model produced from *Thermus thermophilus* GcvH protein (pdb 1ONL) after removing the N- and C-termini, flexible loops and changing the sequence to polyalanine. Density modification was implemented using PARROT (Cowtan, 2010) and initial models were build using the automated model building program BUCCANEER (Cowtan, 2006). Model building for all structures were carried out with COOT (Emsley and Cowtan, 2004) and real space refinement with REFMAC5 (Murshudov et al., 1997).

Supplemental References

Barkauskaite, E., Brassington, A., Tan, E.S., Warwicker, J., Dunstan, M.S., Banos, B., Lafite, P., Ahel, M., Mitchison, T.J., Ahel, I., *et al.* (2013). Visualization of poly(ADP-ribose) bound to PARG reveals inherent balance between exo- and endo-glycohydrolase activities. Nature communications *4*, 2164.

Battye, T.G., Kontogiannis, L., Johnson, O., Powell, H.R., and Leslie, A.G. (2011). iMOSFLM: a new graphical interface for diffraction-image processing with MOSFLM. Acta crystallographica. Section D, Biological crystallography *67*, 271-281.

Bond, C.S., and Schuttelkopf, A.W. (2009). ALINE: a WYSIWYG protein-sequence alignment editor for publication-quality alignments. Acta crystallographica. Section D, Biological crystallography *65*, 510-512.

Castano-Cerezo, S., Bernal, V., Rohrig, T., Termeer, S., and Canovas, M. (2015). Regulation of acetate metabolism in Escherichia coli BL21 by protein N(epsilon)-lysine acetylation. Applied microbiology and biotechnology *99*, 3533-3545.

Cowtan, K. (2006). The Buccaneer software for automated model building. 1. Tracing protein chains. Acta crystallographica. Section D, Biological crystallography *62*, 1002-1011.

Cowtan, K. (2010). Recent developments in classical density modification. Acta crystallographica. Section D, Biological crystallography *66*, 470-478.

Evans, P. (2006). Scaling and assessment of data quality. Acta crystallographica. Section D, Biological crystallography *62*, 72-82.

Huson, D.H., and Bryant, D. (2006). Application of phylogenetic networks in evolutionary studies. Molecular biology and evolution *23*, 254-267.

Katoh, K., Kuma, K., Toh, H., and Miyata, T. (2005). MAFFT version 5: improvement in accuracy of multiple sequence alignment. Nucleic acids research *33*, 511-518.

Murshudov, G.N., Vagin, A.A., and Dodson, E.J. (1997). Refinement of macromolecular structures by the maximum-likelihood method. Acta crystallographica. Section D, Biological crystallography *53*, 240-255.

Park, B., Nizet, V., and Liu, G.Y. (2008). Role of Staphylococcus aureus catalase in niche competition against Streptococcus pneumoniae. Journal of bacteriology *190*, 2275-2278.

Savitsky, P., Bray, J., Cooper, C.D., Marsden, B.D., Mahajan, P., Burgess-Brown, N.A., and Gileadi, O. (2010). High-throughput production of human proteins for crystallization: the SGC experience. Journal of structural biology *172*, 3-13.

Sievers, F., Wilm, A., Dineen, D., Gibson, T.J., Karplus, K., Li, W., Lopez, R., McWilliam, H., Remmert, M., Soding, J., *et al.* (2011). Fast, scalable generation of high-quality protein multiple sequence alignments using Clustal Omega. Molecular systems biology *7*, 539.

Storoni, L.C., McCoy, A.J., and Read, R.J. (2004). Likelihood-enhanced fast rotation functions. Acta crystallographica. Section D, Biological crystallography *60*, 432-438.

Wagner, G.R., and Payne, R.M. (2013). Widespread and enzyme-independent Nepsilon-acetylation and Nepsilon-succinylation of proteins in the chemical conditions of the mitochondrial matrix. The Journal of biological chemistry *288*, 29036-29045.

Waterhouse, A.M., Procter, J.B., Martin, D.M., Clamp, M., and Barton, G.J. (2009). Jalview Version 2--a multiple sequence alignment editor and analysis workbench. Bioinformatics *25*, 1189-1191.
